# Gene complementation analysis indicates that parasitic dodder plants do not depend on the host FT protein for flowering

**DOI:** 10.1016/j.xplc.2024.100826

**Published:** 2024-01-29

**Authors:** Sina Mäckelmann, Andrea Känel, Lara M. Kösters, Peter Lyko, Dirk Prüfer, Gundula A. Noll, Susann Wicke

**Affiliations:** 1 Institute of Plant Biology and Biotechnology, University of Muenster, Schlossplatz 8, 48143 Muenster, Germany; 2 Institute for Biology, Humboldt-University of Berlin, Haus 22, Philippstr. 13, 10115 Berlin, Germany; 3 Fraunhofer Institute for Molecular Biology and Applied Ecology IME, Schlossplatz 8, 48143 Muenster, Germany

Dear Editor,

*Cuscuta,* a genus of parasitic plants, poses a significant threat to global agriculture due to its broad host range that encompasses numerous cultivated crops. The plants form specialized feeding organs (haustoria) that connect to the host vascular tissue and import water, nutrients, secondary metabolites, small RNAs, mRNAs, and proteins. One such protein is FT, a peptide hormone and florigen that was recently proposed to cause the synchronization of flowering in the parasite–host system (Shen et al., 2020). Flowering is tightly controlled by endogenous and environmental cues, ensuring that floral development occurs only under favorable conditions. FT is a major network hub that integrates signals from several floral pathways and is the mobile signal that moves from leaves to the shoot apical meristem, where it interacts with its cofactor FD and 14-3-3 proteins to promote flowering (Taoka et al., 2011; Blümel et al., 2015). Genome analysis in *C. australis* and *C. campestris* indicated the loss of multiple floral regulator genes (Sun et al., 2018; Vogel et al., 2018), suggesting that the translocated host FT protein may complement the incomplete regulatory network in the parasite by forming a complex with endogenous FD to enable floral transition (Shen et al., 2020). However, *Cuscuta* species flower independently *in vitro* (Bernal Galeano et al., 2022). Furthermore, host flowering status has never affected the flowering time of *C. campestris* in our laboratories.

To investigate this in more detail, we used tobacco (*Nicotiana tabacum*) as the host plant, which, like *Cuscuta*, belongs to the order of Solanales. Tobacco is a day-neutral plant that produces two FT floral inducers (NtFT4 and NtFT5) and two FT floral repressors (NtFT1 and NtFT2). NtFT5 is predominantly responsible for floral induction under long-day (LD) conditions, whereas both NtFT4 and NtFT5 induce flowering under short-day (SD) conditions. NtFT1 is responsible for floral repression under LD conditions, whereas both NtFT1 and NtFT2 repress flowering under SD conditions (Harig et al., 2012; Beinecke et al., 2018). First, we generated non-flowering tobacco (*N. tabacum*) host plants by mutating the two tobacco floral activators NtFT4 and NtFT5 (Schmidt et al., 2020; Supplemental Figure 1). We allowed *C. campestris* to parasitize wild-type tobacco and two independent *Ntft4*^*–*^*Ntft5*^*–*^ T_2_ lines. The wild-type plants flowered after 66 days, whereas onset of flowering had not occurred in *Ntft4*^*–*^*Ntft5*^*–*^ plants by the end of the experiment (83 days). *C. campestris* plants parasitizing wild-type tobacco flowered after 42 days, and those parasitizing the two double-mutant hosts flowered after 39 and 42 days, respectively (Figure 1A). Conversely, we tested whether FT overexpression in the host influences *C. campestris* flowering time. As NtFT5 overexpression in the wild-type tobacco background causes extremely early flowering, making infestation with *Cuscuta* impossible, we used tobacco hosts with a knockout mutation in NtFT5 and simultaneous overexpression of NtFT5 using the constitutive 35S promoter (35S:*NtFT5*_L4_//SR1*ΔNtFT5*), whose flowering time is shortened by approximately 20% compared with that of the controls (Zimmermann et al., 2022). We found that flowering time of *Cuscuta* did not change significantly when feeding on the 35S:*NtFT5*_L4_//SR1*ΔNtFT5* plants compared with control hosts (Supplemental Figure 2). These data suggest that *C. campestris* does not rely on the host’s FT signal.

**Figure 1:**
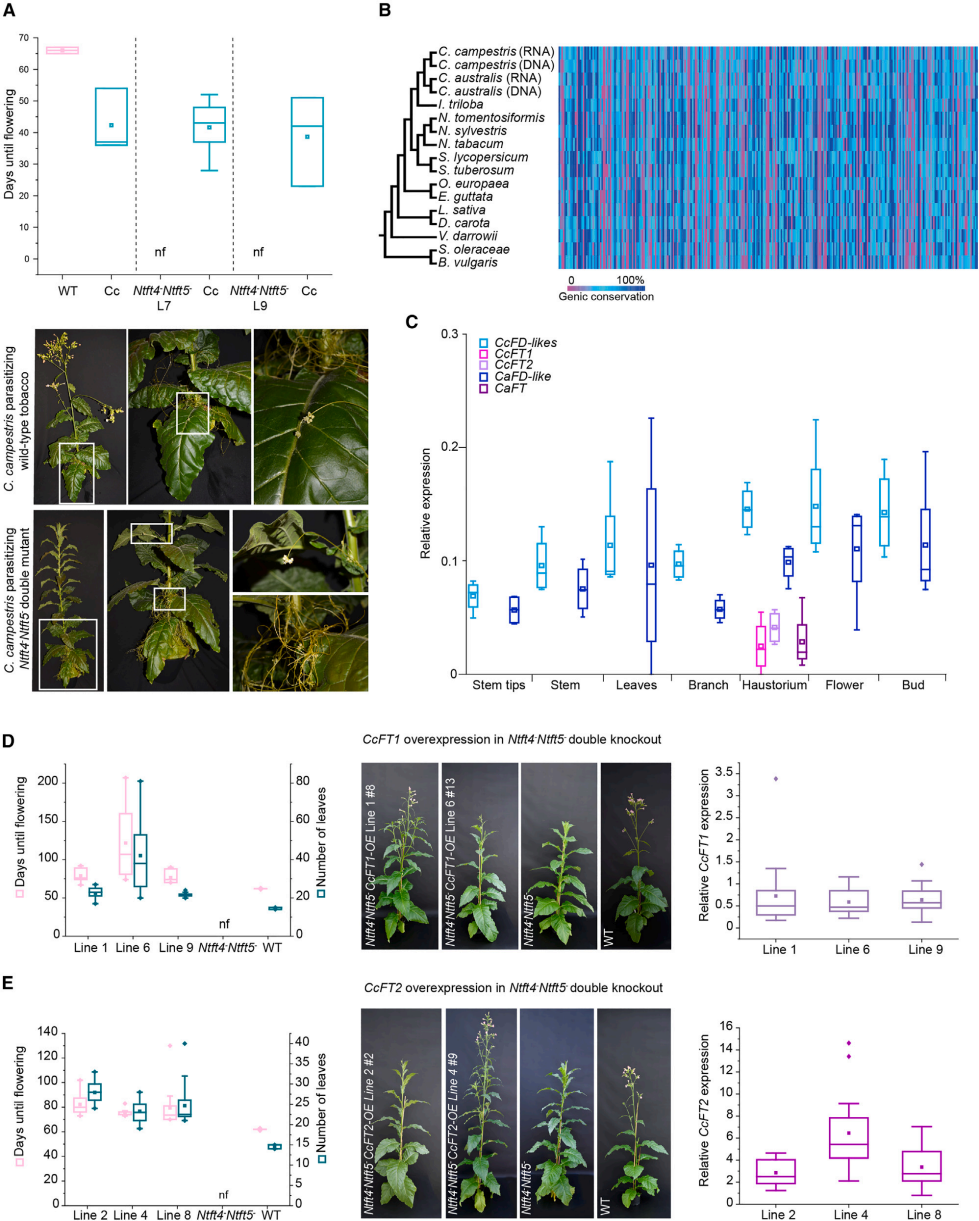
Analysis of flowering, conservation of flowering genes (heatmap), expression analysis, and overexpression experiments in *Cuscuta* plants to investigate the role of host FT. **(A)** *Cuscuta campestris* was allowed to parasitize tobacco SR1 wild-type (WT) plants or two independent *Ntft4*^*–*^*Ntft5*^*–*^ double-knockout mutants (lines 7 and 9). The presence or absence of floral activating FTs in the host did not influence *C. campestris* flowering time, as determined by analysis of variance and Tukey’s post hoc test (n ≥ 3 biological replicates; nf, non-flowering until the end of experiment, 83 days after sowing). Photos were taken 83 days after sowing (magnified areas are indicated by white squares). **(B)** Heatmap showing the conservation of flowering-time genes (and corresponding coding regions) in the genomes of *Cuscuta* spp. and 13 closely related eudicots. Small blocks in columns represent each of the 295 protein-coding genes associated with flowering time (detailed in Supplemental Data 1). Regarding gene conservation, blue indicates a conserved length and amino acid sequence, cyan shows genic drift, and magenta indicates the absence of a coding region. Each line corresponds to one of the analyzed 13 eudicots and *Cuscuta* spp., whose vertical order is displayed in a phylogenetic context as illustrated on the left-hand side. For the two *Cuscuta* species, *C. australis* and *C. campestris*, gene conservation was analyzed at both the genome data level and the RNA (transcriptome) level, as indicated by “DNA” or “RNA,” respectively. Further details of the analysis are available in the supplemental materials and methods and Supplemental Data 1. **(C)** Expression analysis of *FD* and *FT* homologs in different *Cuscuta* tissues. *C. campestris* tissue was harvested from flowering plants parasitizing WT tobacco. *C. australis* tissue was harvested from flowering plants parasitizing soybean. *CcFD-like1* and *CcFD-like2* expression levels were determined using non-discriminating primers. Relative expression levels of *CcFT1*, *CcFT2*, *CcFD*-*like* genes, *CaFT1*, and *CaFD*-*like* were determined by RT–qPCR using *Actin* and *EF1α* as reference genes (*n* = 4 biological replicates). **(D)** Overexpression of *CcFT1* in *Ntft4*^*–*^*Ntft5*^*–*^ tobacco plants. Flowering time and number of leaves at flowering of *Ntft4*^*–*^*Ntft5*^*–*^ plants overexpressing *CcFT1* compared with *Ntft4*^*–*^*Ntft5*^*–*^ and WT controls. Days until flowering and number of leaves were determined when the first bud had fully opened. Data represent *n* = 17 (line 1), 15 (line 6), 14 (line 9), 12 (*Ntft4*^*–*^*Ntft5*^*–*^), and 6 (WT) plants. Statistics for the individual treatment groups were not assessed, as no data points were available for non-flowering plants. Phenotypes of *Ntft4*^*–*^*Ntft5*^*–*^ plants with (exemplarily shown for line 1 and line 6) or without (*Ntft4*^*–*^*Ntft5*^–^) heterologous expression of *CcFT1* compared with WT controls were photographed when WT plants flowered. Relative expression levels of *CcFT1* in *Ntft4*^*–*^*Ntft5*^*–*^ plants overexpressing *CcFT1* were determined by RT–qPCR using *NtEF1α* as the reference gene (nf, non-flowering until end of experiment, 233 days after sowing). **(E)** Overexpression of *CcFT2* in *Ntft4*^*–*^*Ntft5*^*–*^ double mutants of tobacco. Flowering time and number of leaves at flowering of *Ntft4*^*–*^*Ntft5*^*–*^ plants overexpressing *CcFT2* compared with *Ntft4*^*–*^*Ntft5*^*–*^ and WT controls. Days until flowering and number of leaves were determined when the first bud had fully opened. Data represent *n* = 12 (line 2), 17 (line 4), 18 (line 8), 13 (*Ntft4*^*–*^*Ntft5*^*–*^), and 6 (WT) plants. Statistics for the individual treatment groups were not assessed, as no data points were available for non-flowering plants. Phenotypes of *Ntft4*^*–*^*Ntft5*^*–*^ plants with (exemplarily shown for line 2 and line 4) or without (*Ntft4*^*–*^*Ntft5*^–^) the heterologous expression of *CcFT2* compared with WT controls were photographed when WT plants flowered. Relative expression levels of *CcFT2* in *Ntft4*^*–*^*Ntft5*^*–*^ plants overexpressing *CcFT2* were determined by RT–qPCR using *NtEF1α* as the reference gene (nf, non-flowering until end of experiment, 233 days after sowing). In the boxplots, center line = median, square = mean, box limits = upper and lower quartiles, whiskers = 1.5× interquartile range, and diamonds = outliers.

To examine the endogenous FT–FD module in *C. campestris*, we compared 295 protein-coding genes associated with flowering time in multiple species, revealing only two putative gene losses specific to *Cuscuta* (*GIBBERELLIN 2-OXIDASE 8* and *UBIQUITIN-SPECIFIC PROTEASE 26*). Other losses and major sequence deviations compared with *Arabidopsis* were shared with other eudicots (Figure 1B; Supplemental Data 1). We sequenced the complete *CcFT1* and *CcFT2* genes and coding sequences, revealing a non-intact *Ty1*/*copia Ale*-type retrotransposon in the third intron of *CcFT2* and a LINE-type reverse transcriptase upstream of *CcFT1* and the *C. australis* ortholog *CaFT* (Supplemental Data 2), but RT–PCR indicated that all *FT* mRNAs were correctly spliced (Supplemental Figure 3). The deduced amino acid sequences of CcFT1 and CaFT were identical. A search for FD homologs revealed seven new *FD*-like sequences in *C. australis* and five in *C. campestris*. The association of FDs with 14-3-3 proteins and their interaction with FTs depends on the phosphorylation of serine/threonine residues in the SAP motif (Taoka et al., 2011). Only two of the *C. campestris FD*-like transcripts contained a SAP-like motif (*CcFD*-like1 and *CcFD*-like2), and their amino acid sequences were 99% identical. We also found a sequence identical to *CcFD-like1* in *C. australis* (*CaFD-like*), which differed from the published *CaFD* sequence that lacks a SAP motif (Supplemental Figure 4) and was reported not to interact with CaFT. We analyzed the expression of the *CcFD*-like and *CcFT* genes and detected strong *CcFD*-like expression in haustoria, buds, and flowers and slightly weaker expression in stem tips, stems, leaves, and branches. We detected *CcFT1* and *CcFT2* expression only in haustoria (low levels in both cases). Similarly, *CaFD-*like genes were expressed in all tissues, whereas *CaFT* was haustorium specific (Figure 1C). These results were confirmed by RNA sequencing analysis of FT, FD, and other floral regulators, which we detected in both species (Supplemental Tables 1 and 2). Together, these results show that *C. campestris* expresses two paralogs each of FT and its cofactor FD, as well as other flowering time regulators that interact with them. In contrast to previous findings (Shen et al., 2020), we also detected *CaFT* and *CaFD*-like gene expression in *C. australis*, probably because we analyzed *CaFT* and *CaFD*-like expression in different plant organs, whereas Shen et al. (2020) focused on different developmental stages of stem tissues. In addition, bimolecular fluorescence complementation revealed that both CcFT1 and CcFT2 interacted with CcFD-like1 in the nucleus of *Nicotiana benthamiana* epidermal cells in transient expression experiments. CcFT1 and CcFT2 also interacted with NtFD1, and CcFD-like1 interacted with NtFT5 (Supplemental Figure 5). These results highlight the conservation of the FT–FD module across species. Taken together, these results indicate that an endogenous FT–FD protein complex can form in both *Cuscuta* species.

To test whether CcFT1 is a functional floral activator, we overexpressed its coding sequence in the wild-type tobacco background. We found that CcFT1 overexpression failed to induce early flowering (Supplemental Figure 6), indicating that CcFT1 is unable to surpass the function of the two endogenous floral activators, NtFT4 and NtFT5. To avoid the endogenous expression of activating FTs, we determined the ability of CcFT1 and CcFT2 to complement the non-flowering phenotype of *Ntft4*^*–*^*Ntft5*^*–*^ plants. Under LD conditions, *Ntft4*^*–*^*Ntft5*^*–*^ plants overexpressing *CcFT1* flowered 67–207 days after sowing (Figure 1D). Only two of 48 plants remained vegetative after 233 days, probably owing to low *CcFT1* expression levels (Supplemental Figure 7). *Ntft4*^*–*^*Ntft5*^*–*^ plants overexpressing *CcFT2* flowered 70–130 days after sowing, whereas the *Ntft4*^*–*^*Ntft5*^*–*^ control plants had not flowered after 233 days (Figure 1E). Higher *CcFT1* and *CcFT2* expression in the complemented *Ntft4*^*–*^*Ntft5*^*–*^ plants were both significantly correlated with earlier flowering (Spearman rank correlation, one-tailed, *CcFT1*: *r*s = −0.2885, *p* = 0.023, *n* = 48; *CcFT2*: *r*_s_ = −0.6546, *p* < 0.001, *n* = 47) and fewer leaves until flowering (*CcFT1*: *r*_s_ = −0.3425, *p* = 0.009, *n* = 48; *CcFT2*: *r*_s_ = −0.6658, *p* < 0.001, *n* = 47; Supplemental Table S6). In contrast to Shen et al. (2020), we used an enhanced version of the CaMV35S promoter in conjunction with the TMV Ω leader to achieve optimal translation initiation efficiency for all our CcFT overexpression experiments, which revealed that CcFT1 also induces early flowering in *Arabidopsis thaliana* Col-0 wild-type plants under SD conditions (Supplemental Figure 8). We observed that increased FT gene expression coincided with early flowering (Spearman rank correlation, one-tailed: *r*s = −0.3404, *p* = 0.048, *n* = 25), expedited rosette development (*r*_s_ = −0.5187, *p* = 0.004, *n* = 25) and cauline leaf formation (*r*_s_ = −0.5579, *p* = 0.002, *n* = 25). These results show that CcFT1/CaFT and CcFT2 can induce floral transition in a heterologous system and should be considered functional floral activators.

Our data show that *C. campestris* does not synchronize its flowering time with the host and can induce flowering without a host FT signal. Furthermore, *C. campestris* and *C. australis* express endogenous *FT* and *FD-like* orthologs. *Cuscuta* species also produce sensors of both short-wave and long-wave light (Supplemental Data 1) that act upstream of FT. This aligns with *in vitro* cultivation protocols, which use photoperiod shifts to induce flowering in host-free *Cuscuta* plants (Baldev, 1962). An earlier claim that the *Cuscuta* flowering time network is impaired was based on the inferred loss of genes with key roles in *Arabidopsis*, such as CO and FLC (Sun et al., 2018; Shen et al., 2020). Although CO is present in several angiosperm lineages (e.g., monocots), its role as a positive regulator of photoperiod-dependent floral induction is limited outside the Brassicaceae. The CO pathway evolved from CO-like genes following gene duplication within this family (Simon et al., 2015). Moreover, the vernalization response, mainly controlled by FLC in *Arabidopsis*, has convergently evolved after the continental drift. For example, it has been shown that FLC homologs are not involved in the vernalization response pathways of cereals and Amaranthaceae (supplemental information). Floral transition genes have been studied in many species other than *Arabidopsis*, revealing numerous alternative genetic networks that regulate flowering time (Blümel et al., 2015). In potato (*Solanum tuberosum*), a close relative of tobacco, activating and repressing FT homologs not only control sexual reproduction but also vegetative reproduction by coordinating the floral transition and tuberization (supplemental information). Sugar beet (*Beta vulgaris*; Chenopodiaceae) has no functional CO ortholog, and its FLC homolog does not play a major role in the vernalization response (supplemental information). Instead, sugar beet produces a pair of antagonistic FT homologs that are differentially regulated in annuals and biennials by the joint action of BTC1 and BvBBX19, which together may integrate temperature and photoperiod signals (supplemental information). These are clear examples in which floral regulating pathways diverge from the *Arabidopsis* model, showcasing adaptations that have evolved during the diversification of angiosperms and their colonization of different environmental niches (Blümel et al., 2015).

The expression of *Cuscuta* FT homologs was detected only in the haustoria, not in other tissues. In parasitic plants, perception of the host’s physiological status is likely to facilitate successful reproduction by ensuring a sufficient energy supply for growth, floral induction, seed maturation, and ripening. This agrees with the fact that *FT* expression depends not only on endogenous cues such as hormones and plant age and seasonal cues such as temperature or photoperiod but also on carbon assimilation rates and sugar levels (supplemental information). Indeed, excised *C. campestris* stem tips only require sugars for growth and flowering (supplemental information). It remains unclear whether and to what extent the host contributes to floral induction in *Cuscuta*. A combination of direct experimental and circumstantial evidence suggests that the host provides physiological cues to induce flowering in the parasite but that the genetic components of the parasite’s floral induction pathway are endogenous. We have demonstrated that *C. campestris* and *C. australis* possess an endogenous FT–FD module, providing a reference point for identification of the upstream regulators and downstream targets.

## Data and code availability

All sequence data reported herein, including target-assembled protein-coding genes, alignment files, and gene family trees, as well as unscaled figures, have been published in standardized, reusable .fasta, .nexus, and .newick file formats at the Dryad Data Repository (https://datadryad.org/stash/share/DK8Olh2VqFwbGNL0GtkGt24dD0GhWhJn82oLBC1XK70). All newly generated sequence data have been deposited at NCBI GenBank under accession numbers NCBI: OP995431–OP995444.

## Funding

This study was supported by the German Science Foundation (10.13039/501100001659DFG, WI4507/3-1 to S.W.) and the Fraunhofer internal funding program PREPARE to G.A.N. and D.P.

## Author contributions

Conceptualization, S.M., A.K., G.A.N., and S.W.; investigation, S.M., A.K., L.M.K., and P.L.; validation, S.M. and A.K.; visualization, S.M., A.K., and S.W.; writing – original draft, A.K., D.P., G.A.N., and S.W.; writing – review & editing, D.P., G.A.N., and S.W.; data analysis, L.M.K., P.L., and S.W.; funding acquisition, D.P. and S.W.; supervision, D.P.; project administration, G.A.N. and S.W.

## Supplemental information

Document S1. Supplemental materials and methods, Supplemental Figures 1‒8, and Supplemental Tables 1‒6

Supplemental Data 1. Results of Exonerate-based genic conservation analysis of 295 flowering-time regulating genes in *Cuscuta* spp. and 13 related eudicots

Supplemental Data 2. Domain-based annotation of transposable elements (DANTE) and inferred fragments

Document S2. Article plus supplemental information
