# Supplementary material for: Gene complementation analysis indicates that parasitic dodder plants do not depend on the host FT protein for flowering
Source: Plant Commun. 2024 Jan 29;5(5):100826. doi: 10.1016/j.xplc.2024.100826 (PMC11121739; doi:10.1016/j.xplc.2024.100826)
Supplement: Document S1. Supplemental materials and methods, Supplemental Figures 1‒8, and Supplemental Tables 1‒6 [file mmc1.pdf]

**Plant Communications, Volume *5***

## Supplemental information

# Gene complementation analysis indicates that parasitic dodder plants do not depend on the host FT protein for flowering

**Sina Mäckelmann, Andrea Känel, Lara M. Kösters, Peter Lyko, Dirk Prüfer, Gundula A. Noll, and Susann Wicke**

# Supplemental Information

## Gene complementation analysis indicates that parasitic dodder plants do not depend on the host FT protein for flowering

Sina Mäckelmann[1¶], Andrea Känel[1¶], Lara M. Kösters[2], Peter Lyko[2], Dirk Prüfer[1,3], Gundula A. Noll[1,3*], and Susann Wicke[2*]

[1] Institute of Plant Biology and Biotechnology, University of Muenster, Schlossplatz 8, 48143, Muenster, Germany

[2] Institute of Biology, Humboldt-Universität zu Berlin, Haus 22, Philippstr. 13, 10115, Berlin, Germany

[3] Fraunhofer Institute for Molecular Biology and Applied Ecology IME, Schlossplatz 8, 48143, Muenster, Germany

[¶] These authors contributed equally to this work

* Corresponding authors: Gundula A. Noll, Susann Wicke

Email: gnoll@uni-muenster.de; susann.wicke@hu-berlin.de

## Supplemental materials and methods

**Additional information on study background and experimental design.** Due to constraints on word count and citation numbers, it was not feasible to incorporate all pertinent background information into the main text. Nevertheless, we wish to acknowledge the following studies for their crucial contributions: Ream et al. (2012) conducted a comprehensive review of the FLC-independent vernalization response in Amaranthaceae and cereals. Navarro et al. (2015) delved into the functional analysis of antagonistically acting FTs that govern sexual and vegetative reproduction in potatoes. Chia et al. (2008) and Vogt et al. (2014) investigated the roles of CO and FLC homologues in *Beta vulgaris*, respectively. Pin et al. (2010, 2012) and Dally et al. (2018) explored antagonistic FT proteins from *Beta vulgaris* and their regulators BTC1 and BvBBX19. Additionally, Cho et al. (2018) provided insights into the role of sugars in flowering time control. Notably, Loo (1946) demonstrated the sole requirement of sugars for growth and flowering in tissue culture of *Cuscuta campestris* stem tips.

**Plant material and cultivation conditions.** This study used tobacco (*Nicotiana tabacum* L. cv. Petit Havana SR1), *Ntft5*$^{-}$ mutant plants originating from the nullizygous $T_2$ progeny of line #78 without the pDECAS9-*NtFT5*$_{ex\ I\text{-}147.169bp}$ transgene (Schmidt et al., 2020), *Ntft5*$^{-}$ mutant plants which overexpress NtFT5 under control of the 35S promoter (35S:*NtFT5*$_{L4}$//SR1Δ*NtFT5*; Zimmermann et al., 2022), *Arabidopsis thaliana* Col-0 and soybean (*Glycine max* cv. Summer Shell). If not stated otherwise, plants were cultivated under long-day (LD) conditions in a climate-controlled greenhouse (16-h photoperiod, artificial light switched on if natural light fell below 700 µmol $m^{-2}$ $s^{-1}$, 22–25 °C under light, 19–25 °C in the dark). Alternatively, plants were cultivated in phytochambers under LD conditions (16-h photoperiod, 200 µmol $m^{-2}$ $s^{-1}$, 25–27 °C (tobacco) or 22°C (*Arabidopsis*) under light, 20 °C in the dark) or under short-day (SD) conditions in phytochambers (8-h photoperiod, 200 µmol $m^{-2}$ $s^{-1}$, 25–27 °C under light, 20 °C in the dark). *Cuscuta* spp. originated from the previous study material of the UIT Arctic University of Norway and the Kunming Institute of Botany (Vogel et al., 2018; Sun et al., 2018) and were harvested from the living research collections at the University of Münster and the Späth Arboretum, Humboldt-Universität zu Berlin. Before germination and infection of host plants, *Cuscuta* spp. seeds were immersed in 97% sulfuric acid for 20 min and then rinsed with autoclaved tap water 3–4 times. Afterwards, the seeds were placed on wet filter paper in Petri dishes and transferred to the greenhouse for germination. Seedlings 2–4 cm in length were placed near the stems of tobacco at the 2–3 true leaf stage, whereas soybean was parasitized as soon as the plants reached ~10 cm in height. A low red/far-red light ratio, facilitating parasitism (Tada et al., 1996), was established using far-red (730-nm) LEDs with a photon flux density of 281.4 µmol $m^{-2}$ $s^{-1}$ in addition to the standard greenhouse lights until the first haustorial connection had developed.

**Identification and validation of genomic and coding sequences.** Genomic DNA was isolated from *C. campestris* using the NucleoSpin Plant II Kit (Machery Nagel, Düren, Germany) and the genes were amplified in overlapping fragments using Phusion high-fidelity polymerase (Thermo Fisher Scientific, Waltham, MA, USA) and the primers listed in Supplemental Table 5. Gene fragments were subcloned into pCRIITopo (Thermo Fisher Scientific) and sequenced, revealing the complete genomic sequence of *CcFT1* (11,502 bp) and *CcFT2* (12,819 bp). For the amplification of the full length cDNAs and to confirm correct splicing of *Cuscuta FT* mRNAs, the 3′-UTR was identified by 3′-RACE. RNA was isolated from *C. campestris* or *C. australis* haustoria using the PureLink RNA Mini Kit (Thermo Fisher Scientific) and reverse transcribed using Superscript II reverse transcriptase (Thermo Fisher Scientific) and the 3`Oligo_d(T) primer (Supplemental Table 5). The cDNAs were used as templates for PCR with Phusion high-fidelity polymerase and the GSP1 fw and 3`Adap (Supplemental Table 5). The PCR product was diluted 1:20 and used as a template for nested PCR with Phusion high-fidelity polymerase and the primers GSP2 fw and 3`Adap_nested (Supplemental Table 5). The amplicons were purified, subcloned into pCR™II-Topo™ (Thermo Fisher Scientific) and sequenced. Based on the identified 3′-UTR sequence, *CcFT1*, *CcFT2* and *CaFT* full-length coding sequences including parts of the 3′-UTR were amplified using the primers listed in Supplemental Table 5, subcloned into pCR™II-Topo™ (Thermo Fisher Scientific) and verified by sequencing. The coding sequences of *CcFT1* and *CaFT* were 534 bp in length and the coding sequence of *CcFT2* was 531 bp in length.

**Quantitative real-time PCR.** Plant tissues were harvested, snap-frozen in liquid nitrogen and ground using either an MM400 bead mill (Retsch, Haan, Germany) or with a mortar and pestle under liquid nitrogen. Tobacco, *Arabidopsis* and soybean RNA was extracted using the innuPREP Plant RNA kit

(Analytik Jena, Jena, Germany) and *Cuscuta* spp. RNA was extracted using the PureLink RNA Mini Kit. Residual genomic DNA was digested using the TURBO DNA-free kit (Thermo Fisher Scientific). Following reverse transcription using PrimeScript RT master mix (Takara Bio, San Jose, CA, USA), gene expression was analyzed by quantitative real-time PCR using Kapa SYBR Fast qPCR Master Mix (Merck, Darmstadt, Germany), the primers listed in Supplemental Table 5, and the CFX96 Real-Time System (Bio-Rad Laboratories, Hercules, CA, USA). Each reaction was carried out in technical triplicates. Specificity was ensured by melt curve analysis, the sequencing of PCR products, and by including no-template and no-reverse-transcription controls. Because residual host tissues may have been co-harvested with the haustoria, we also tested tobacco and soybean cDNA with the same primer sets (Supplemental Table 5). We did not detect any amplicons, confirming the primer specificity. Individual PCR efficiency was determined using LinReg PCR v2017.0 and relative gene expression levels were normalized to *EF1α* and *Actin* (*Cuscuta* spp.), *PP2* and *Ubi* (*A. thaliana*), *EF1α* (*N. tabacum*), or *F-Box* (*G. max*).

**Cloning.** For the targeted editing of *NtFT4*, the binary construct pDECAS9-*NtFT4*$_{\text{ex I-59.79 bp}}$ was cloned as previously described (Schmidt et al., 2020). The original plasmids were kindly provided by Holger Puchta (Karlsruhe Institute of Technology, Karlsruhe, Germany). For BiFC assays, *CcFT1*, *CcFT2* and *CcFD-like1* were amplified from cDNA using primers with attached restriction sites (Supplemental Table 5) and were transferred to pENTR4 (Thermo Fisher Scientific) by restriction and ligation. Subsequent transfer to pBatTL vectors was achieved by Gateway recombination (pBatTL plasmids were kindly provided by Joachim Uhrig and Guido Jach, University of Cologne, Cologne, Germany). BiFC constructs containing *NtFD1* and *NtFT5* were available from previous studies (Beinecke et al., 2018). For the heterologous expression of *CcFT* genes, we amplified the CaMV35S promoter sequence including the TMV Ω leader from pFGC5941-GW (www.chromdb.org) using the splicing by overlap extension (SOE)-PCR technique to eliminate the internal XhoI site. The amplicon was then transferred to the SmaI/XhoI sites of pLab12.10 (Xing et al., 2014) or pLab12.1 (Post et al., 2012) to generate pLab12.10Q35S-P and pLab12.1Q35S-P, respectively. The vector backbones carry different resistance cassettes, with pLab12.10 conferring kanamycin resistance and pLab12.1 conferring phosphinothricin resistance. *CcFT1* and *CcFT2* were amplified from cDNA using primers with attached restriction sites (Supplemental Table 5) and were transferred to pLab12.10Q35S-P and pLab12.1Q35S-P, respectively, by restriction and ligation.

***Agrobacterium*-mediated stable plant transformation.** For the generation of *Ntft4*$^{-}$*Ntft5*$^{-}$ double knockout plants, the previously described nullizygous $T_2$ progeny of *Ntft5*$^{-}$ mutant line #78 without the pDECAS9-*NtFT5*$_{\text{ex I-147.169bp}}$ transgene (Schmidt et al., 2020) was used for transformation. For functional complementation studies, *Ntft4*$^{-}$*Ntft5*$^{-}$ double knockout plants ($T_2$ generation) were used for transformation with binary plasmids carrying *CcFT1* or *CcFT2* under the control of the 35S promoter (Q35S-P). Transformation was carried out using the leaf disc method (Horsch et al.,1985) with *Agrobacterium tumefaciens* strain LBA4404 for heterologous gene expression, or EHA105 for CRISPR/Cas9-mediated genome editing. The appropriate binary vectors were introduced into these strains by electroporation. For the selection of transgenic plants, MS medium was supplemented with 100 mg/L kanamycin or 3 mg/L phosphinothricin, as appropriate. After callus regeneration and rooting in sterile culture medium, independent transgenic plant lines were cultivated in soil under LD conditions in the greenhouse as described above. Transgene integration was verified by PCR and, depending on the constructs, plants were either analyzed for edited targets (see below) or transgene expression levels were determined by qRT-PCR. For seed production, *Ntft4*$^{-}$*Ntft5*$^{-}$ double knockout scions were grafted onto SR1 wild-type stocks, whereas *Ntft4*$^{-}$*Ntft5*$^{-}$ double knockout plants overexpressing *CcFT1* or *CcFT2* flowered spontaneously. The $T_1$ progeny were used for further experiments.

For the generation of transgenic *A. thaliana* Col-0 plants, wild-type plants were transformed with *Agrobacterium tumefaciens* strain EHA105 carrying the binary plasmid pLab12.10Q35S-P::*CcFT1* using the floral dip method (Clough and Bent, 1998). For the selection of transgenic plants, ½ MS medium was supplemented with 25 mg/L kanamycin. Transgene integration was verified by PCR and independent transgenic plant lines were cultivated in soil under LD conditions in phytochambers. For phenotypic analysis, $T_1$-plants of three independent lines were cultivated in soil under SD conditions in

phytochambers. Transgene expression levels were determined by qRT-PCR using the primers listed in Supplemental Table 5.

**Identification and screening of genome-edited plants.** The *NtFT5* locus in the mutant plants (*Ntft5*$^{-}$ and *Ntft4*$^{-}$*Ntft5*$^{-}$) was analyzed by isolating genomic DNA using the NucleoSpin Plant II Kit and amplifying exon I using Phusion high-fidelity polymerase and the primers listed in Supplemental Table 5. The amplicon was verified by sequencing. After regenerating *Ntft5*$^{-}$ plants transformed with pDECAS9-*NtFT4*$_{\text{ex I-59.79 bp}}$, transgene integration was verified by the isolation of genomic DNA followed by PCR using the 2x *i*-MAX Mastermix (INtRON Biotechnology, Seongnam, Korea) and the primers listed in Supplemental Table 5. For the analysis of genome editing in $T_0$ plants, parts of exon I were amplified using Phusion high-fidelity polymerase and the primers listed in Supplemental Table 5. The amplicons were transferred to pCRIITopo and sequenced. Promising plants were cultivated for seed production and the $T_1$ progeny were analyzed to confirm the knockout mutations. For the analysis of genome editing in $T_1$ plants, parts of exon I were amplified using the Phusion high-fidelity polymerase and the primers listed in Supplemental Table 5. The amplicons were either transferred to pCRIITopo for sequencing (if the plants carried different mutations in their two *NtFT4* alleles) or were sequenced directly (if the same mutation was present in both alleles).

**Phenotyping.** For tobacco, we counted the number of days from seed sowing to full opening of the first floral bud, and the number of leaves when the first floral bud fully opened. For *Cuscuta* spp., we counted the number of days from the formation of the first haustorial connection until the first floral bud had fully opened. For *Arabidopsis*, we counted the number of days from seed sowing to full opening of the first floral bud as well as the number of rosette leaves and cauline leaves when the first floral bud fully opened.

**Infiltration of *Nicotiana benthamiana*.** For transient expression of BiFC constructs, *A. tumefaciens* strain GV3101 pMP90 was transformed with the corresponding binary vectors by electroporation. *N. benthamiana* plants were cultivated in the greenhouse (16-h photoperiod) until they were 4–5 weeks old. The leaves were then infiltrated with *A. tumefaciens* strain GV3101 pMP90 carrying the appropriate plasmids ($OD_{600}$ = 0.25) and *A. tumefaciens* strain C58C1 ($OD_{600}$ = 0.3) carrying the pCH32 helper plasmid and the pBin61 plasmid encoding the RNA silencing suppressor p19 from tomato bushy stunt virus (Walter et al., 2004). Plants were cultivated under continuous light for 3–4 days, and leaf discs were screened for fluorescent cells in the abaxial epidermis.

**Microscopy.** For BiFC experiments, fluorescence was analyzed using a STELLARIS 8 confocal laser scanning microscope (Leica Microsystems, Wetzlar, Germany) at excitation/emission wavelengths of 549/569–629 nm for reconstituted mRFP. Interaction was confirmed if at least five independent images showing fluorescence were captured.

**Bioinformatic recovery of flowering time genes.** We extracted the full set of potentially functional flowering genes and corroborated gene losses in *C. campestris* and *C. australis* based on the genomic sequences of both species from NBCI GenBank (Bioprojects: PRJEB19879 and PRJNA394036), in addition to assembling and querying RNA-Seq data covering key developmental stages (Supplemental Table 2). Genome data alongside published annotations of 13 closely related eudicot species (*Ipomoea triloba* – PRJNA574454; *Beta vulgaris* – assembly EL10_1.0; *Spinacia oleraceae* – assembly ASM200726v1; *N. tabacum* – assembly Ntab-TN90; *N. sylvestris* – assembly Nsyl; *N. tomentosiformis* – assembly Ntom_v01; *Vaccinium darrowii* – assembly USDA_Vadar_1.0_pri; *Solanum lycopersicum* – assembly SL3.1; *Solanum tuberosum* – assembly SolTub_3.0; *Erythranthe guttata* – assembly Mimgu1_0; *Olea europaea* var. *sylvestris* – assembly O_europaea_v1; *Lactuca sativa* – assembly Lsat_Salinas_v8 and *Daucus carota* subsp. *sativus* – assembly ASM162521v1) were included for comparative evolutionary analysis of gene conservation. To account for differences in the quality of genomic data depending on the annotation version, we used a two-stage flowering time gene analysis in which a primary query for flowering time genes in all species was applied to the raw (annotation-free) genomic assembly and hits were then cross-validated and refined using readily available gene annotations/predictions. The two *Cuscuta* species differ significantly in their coding capacity (Vogel et al., 2018; Sun et al., 2018; Lyko and Wicke, 2021), so we also reciprocally searched their genomes for missing protein-coding genes using blastX (e-value cutoff = $10^{-3}$) and exonerate (protein2genome mode; min length coverage 70%), and compared these data to the closest, nonparasitic relative *Ipomoea triloba*, which belongs to the same family as *Cuscuta* (Convolvulaceae).

This approach retrieved many originally “missing” genes (DRYAD data repository, https://datadryad.org/stash/share/DK8Olh2VqFwbGNL0GtkGt24dD0GhWhJn82oLBC1XK70), so we used the BRAKER pipeline to re-annotate *Cuscuta* spp. and *Ipomoea nil* (assembly Asagao_1.1; Bioprojects: PRJNA344313, PRJDB4356) for a direct comparison of the new *ab initio* gene model predictions. Training Augustus in the BRAKER pipeline followed the steps described for *C. campestris* (Vogel et al., 2018), whereby we also used the odp10 protein database plus *I. nil* and *I. triloba* proteins. Our annotation revealed an almost perfect 2:1 gene annotation ratio between *C. campestris* and *C. australis*. Given that an analysis of syntenic blocks across *Cuscuta australis*, *C. campestris* and *Ipomoea triloba* computed using the Doerr & Moret (2017) pipeline found counterparts with conserved genomic orientation on all genomic fragments longer than three million bases in both parasite species (DRYAD:https://datadryad.org/stash/share/DK8Olh2VqFwbGNL0GtkGt24dD0GhWhJn82oLBC1XK70) we conclude that the genome assemblies may be available in a collapsed vs. uncollapsed allelic/pseudochromosomal configuration, rather than reflecting a ploidization event since the parasite species diverged. For the purpose of screening for flowering genes, we merged these new gene sets with the corresponding original ones and removed redundancy in cases of perfect matches. The genomic sets were used to seed-search for 295 protein-coding genes implicated in flowering time regulation using *exonerate* in protein2genome mode (https://www.ebi.ac.uk/about/vertebrate-genomics/software/exonerate), allowing for introns between 15 bp and 35 kb. Reference flowering time proteins were obtained from the FLOR-ID database http://www.phytosystems.ulg.ac.be/florid/ (Bouché et al., 2016) and the Araport11 genome release of *Arabidopsis thaliana* (https://www.arabidopsis.org/index.jsp). We excluded all 11 miRNAs involved in flowering time regulation. In addition, the exonerate-based search for flowering time genes using the Arabidopsis reference gene set was repeated for *C. australis* and *C. campestris* transcriptome assemblies, using all RNA-Seq datasets summarized in Supplemental Table 2. *Cuscuta* spp. transcriptomes were assembled *de novo* using the Trinity RNA-Seq pipeline with default settings (Haas et al., 2013). Recovered hit sequence seeds were then used to extend the region bidirectionally by iteratively mapping genomic and transcriptomic sequence data using *bowtie*2, whereby up to 10 iterations were performed. The exonerate wrapper and all retrieved seed sequence alignments are available in DRYAD: https://datadryad.org/stash/share/DK8Olh2VqFwbGNL0GtkGt24dD0GhWhJn82oLBC1XK70. To compare the divergence in sequence conservation of *Cuscuta* spp. flowering time genes with the corresponding homologous gene models of 13 other eudicot species, we ran *exonerate* in protein2genome mode (as above). Matched protein-coding gene regions were extracted, aligned using *mafft* in auto mode (Katoh and Standley, 2013), and evaluated for the highest-scoring coding sequence length and sequence divergence, relative to functionally validated *Arabidopsis* references (% conservation of gene models). Finally, we used phylogeny-aware domain-based examination, for which we extracted the full gene models matching reference FD and FT sequences from the combined genomic annotations. We built FT and FD alignments using *mafft* in e-ins-i mode with a gap penalty of 1.93. From these data, we reconstructed the protein relationships with RAxML (Stamatakis, 2014) with 200 bootstrap replicates, using the WAG model of amino acid substitution with a γ rate distribution.

**Transposable element analysis.** To check whether transposable element insertion may have rendered FT or FD genes nonfunctional, we used domain-based annotation of transposable elements (DANTE; https://github.com/kavonrtep/dante). We extracted the extended gene regions (–20 kbp and +20 kbp of the entire genes of both species), allowing us to retrieve full-length mobile DNA elements, if any. DANTE was run with Viridiplantae 3.0 as the taxon and protein database and a BLOSUM80 scoring matrix. Single hits were retained if they scored at least 80% similarity to database records. DANTE’s built-in *Protein Domain Filter* tool was then used on the full DANTE annotation and only domains with at least 35% protein sequence identity between input and mapped protein from the database, 45% minimum similarity, and 80% minimum alignment length were considered. We filtered hits with more than three interruptions, such as frameshifts and/or stop codons and insertions resulting in a domain length extension of 1.2 between the new and the reference domain from the database. The resulting filtered and unfiltered evidence for transposable elements was extracted from GFF3 files.

**RNA-Seq gene expression analysis.** We extracted the full coding sequence models of our candidate FD and FT sequences (and for comparison, those of the FT-interacting genes SOC1, CO, LFY, TFL1, and GI) from the combined genomic annotations of both *Cuscuta* species. We assessed gene expression of FT, FD and our selected interacting proteins by mapping all available RNA-Seq data

(Supplemental Table 2) using *bowtie2* in local mode (Langmead and Salzberg, 2012). The unsorted read alignments were parsed to calculate per-reference base coverage using the *pileup* function of the BBMap program suite (Bushnell, 2014; https://github.com/BioInfoTools/BBMap). RNA-Seq expression levels were extremely low for FT (Supplemental Table 1), so its mapping results were double-checked and confirmed by qRT-PCR using highly discriminating primers (Supplemental Table 5).

**Statistics.** All boxplots in the figures were prepared in OriginPro2022 (OriginLab Corporation, Northampton, MA, USA) using default settings (center line = median; square = mean, box limits = upper and lower quartiles; whiskers = 1.5× interquartile range; diamonds = outliers). Statistical analysis was carried out using OriginPro2022. Equality of variances was determined by one-way analysis of variance (ANOVA), and pairwise comparisons were assessed using Tukey's *post hoc* test for multiple comparisons and Student's *t*-test for single pairwise comparisons, unless stated otherwise. If ANOVA revealed significant differences in variances, pairwise comparisons were assessed using ANOVA with Welch´s correction and Games-Howell *post hoc* test for multiple comparisons or Student's *t*-test with Welch´s correction for single pairwise comparisons. Spearman rank correlation tests were conducted in *R* as one-sided tests with alpha (significance level) set to 0.05 (5%), highlighting the strong expectation that higher FT-expression is associated with earlier flowering, therefore expecting a negative correlation with true *r*s being less than 0.

## Supplemental references

**Beinecke, F. A., Grundmann, L., Wiedmann, D. R., Schmidt, F. J., Caesar, A. S., Zimmermann, M., Lahme, M., Twyman, R. M., Prüfer, D., Noll, G. A.** (2018) The FT/FD-dependent initiation of flowering under long-day conditions in the day-neutral species *Nicotiana tabacum* originates from the facultative short-day ancestor *Nicotiana tomentosiformis*. *The Plant Journal* **96**(2):329-342. https://doi.org/10.1111/tpj.14033

**Bouché, F., Lobet, G., Tocquin, P., Périlleux, C.** (2016) FLOR-ID: an interactive database of flowering-time gene networks in *Arabidopsis thaliana*. *Nucleic Acids Res* **44**:D1167-1171. https://doi.org/10.1093/nar/gkv1054

**Bushnell, B.** (2014) BBMap: A Fast, Accurate, Splice-Aware Aligner. https://www.osti.gov/servlets/purl/1241166.

**Chia, T.Y.P., Müller, A., Jung, C., and Mutasa-Göttgens, E.S.** (2008) Sugar beet contains a large CONSTANS-LIKE gene family including a CO homologue that is independent of the early-bolting (B) gene locus. *J. Exp. Bot.* **59**(10): 2735–2748. https://doi.org/10.1093/jxb/ern129

**Cho L. H., Pasriga R., Yoon J., Jeon J. S., An G.** (2018) Roles of sugars in controlling flowering time. *J. Plant Biol.* 2018; **61**: 121-130. https://doi.org/10.1007/s12374-018-0081-z

**Clough S.J., Bent A.F.** (1998) Floral dip: a simplified method for *Agrobacterium*-mediated transformation of *Arabidopsis thaliana*. *The Plant Journal* **16**(6):735-743. https://doi.org/10.1046/j.1365-313x.1998.00343.x

**Dally, N., Eckel, M., Batschauer, A., Höft, N., and Jung, C.** (2018) Two CONSTANS-LIKE genes jointly control flowering time in beet. *Sci. Rep.* **8**(1): 16120. https://doi.org/10.1038/s41598-018-34328-4

**Doerr, D., Moret, B. M. E.** (2018) Sequence-based synteny analysis of multiple large genomes. In *Comparative Genomics, Methods in Molecular Biology*., Setubal, J. C., Stoye, J., Stadler, P. F., Eds. (Springer New York), pp. 317–329.

**Haas, B. J., Papanicolaou, A., Yassour, M., Grabherr, M., Blood, P. D., Bowden, J., ... & Regev, A.** (2013). De novo transcript sequence reconstruction from RNA-seq using the Trinity platform for reference generation and analysis. *Nat. Protoc.* **8**(8):1494-1512. https://doi.org/10.1038/nprot.2013.084

**Horsch, B., Fry, J. E., Hoffmann, N. L., Wallroth, M., Eichholtz, D., Rogers, S. G., Fraley, R.T. (**1985) A simple and general method for transferring genes into plants. *Science* **227**:1229–1231. https://doi.org/doi.org/10.1126/science.227.4691.1229

**Katoh, K., and Standley, D. M.** (2013) MAFFT multiple sequence alignment software version 7: improvements in performance and usability. *Mol. Biol. Evol.* **30**:772–780. https://doi.org/10.1093/molbev/mst010

**Langmead, B., and Salzberg, S. L.** (2012) Fast gapped-read alignment with Bowtie 2. *Nat. Methods* **9**:357–359. https://doi.org/10.1038/nmeth.1923

**Libault, M., Thibivilliers, S., Bilgin, D. D., Radwan, O., Benitez, M., Clough, S. J., Stacey, G.** (2008) Identification of four soybean reference genes for gene expression normalization. *The Plant Genome*, *1*(1). https://doi.org/10.3835/plantgenome2008.02.0091

**Loo S. W.** (1946) Cultivation of excised stem tips of dodder in vitro. *Am. J. Bot.* **33**(4): 295–300. https://doi.org/10.2307/2437436

**Lyko, P., Wicke, S.** (2021) Genomic reconfiguration in parasitic plants involves considerable gene losses alongside global genome size inflation and gene births. *Plant Physiol.* **186:**1412–1423. https://doi.org/10.1093/plphys/kiab192

**Navarro, C., Cruz-Oró, E., and Prat, S.** (2015) Conserved function of FLOWERING LOCUS T (FT) homologues as signals for storage organ differentiation. *Curr. Opin. Plant Biol.* **23**: 45–53. https://doi.org/10.1016/j.pbi.2014.10.008

**Pin, P.A., Benlloch, R., Bonnet, D., Wremerth-Weich, E., Kraft, T., Gielen, J.J.L., and Nilsson, O.** (2010) An antagonistic pair of FT homologs mediates the control of flowering time in sugar beet. *Science* **330** (6009): 1397–1400. https://doi.org/10.1126/science.1197004

**Pin, P.A., Zhang, W., Vogt, S.H., Dally, N., Büttner, B., Schulze-Buxloh, G., Jelly, N.S., Chia, T.Y.P., Mutasa-Göttgens, E.S., and Dohm, J.C., et al.** (2012) The role of a pseudo-response regulator gene in life cycle adaptation and domestication of beet. *Curr. Biol.* CB **22** (12): 1095–1101. https://doi.org/10.1016/j.cub.2012.04.007

**Post, J.J., Eisenreich, W., Huber, C., Twyman, R.M., Prüfer, D. and Schulze Gronover, C. (2013)** Establishment of an ex vivo laticifer cell suspension culture from *Taraxacum brevicorniculatum* as a production system for cis-isoprene. *J. Mol. Catal. B Enzym* **103**:85-93. https://doi.org/doi.org/10.1016/j.molcatb.2013.07.013

**Ream T. S., Woods D. P., Amasino R. M.** (2012) The molecular basis of vernalization in different plant groups. *Cold Spring Harb. Symp. Quant. Biol.* **77**: 105-15. https://doi.org/10.1101/sqb.2013.77.014449

**Rezaei, H., Alamisaeed, K., Moslemkhani, C.** (2017) Overexpression of stress-related genes in *Cuscuta campestris* in response to host defense reactions. *BioTechnologia* **98**(2):131-139 https://doi.org/10.5114/bta.2017.68312

**Schmidt, F. J., Zimmermann, M. M., Wiedmann, D. R., Lichtenauer, S., Grundmann, L., Muth, J., Twyman, R. M., Prüfer, D., Noll, G. A.** (2020) The major floral promoter NtFT5 in tobacco (*Nicotiana tabacum*) is a promising target for crop improvement. *Front. Plant Sci.* 1666. https://doi.org/10.3389/fpls.2019.01666

**Shen G., Liu N., Zhang J., Xu Y., Baldwin I. T., Wu, J.** (2020) *Cuscuta australis* (dodder) parasite eavesdrops on the host plants' FT signals to flower. Proc. Natl. Acad. Sci. U S A. 117(37):23125-23130. https://doi.org/10.1073/pnas.2009445117

**Stamatakis, A.** (2014) RAxML version 8: a tool for phylogenetic analysis and post-analysis of large phylogenies. *Bioinformatics* **30:**1312–1313. https://doi.org/10.1093/bioinformatics/btu033

**Sun, G., Xu, Y., Liu, H., Sun, T., Zhang, J., Hettenhausen, C., Shen, G., Qi, J., Li, J., Wang, L., Chang, W., Guo, Z., Baldwin, I.T., Wu, J.** (2018) Large-scale gene losses underlie the genome evolution of parasitic plant *Cuscuta australis*. *Nat. Commun.* **9**(1):2683. https://doi.org/10.1038/s41467-018-04721-8

**Tada, Y., Sugai, M., Furuhashi, K.** (1996) Haustoria of *Cuscuta japonica*, a holoparasitic flowering plant, are induced by the cooperative effects of far-red light and tactile stimuli. *Plant Cell Physiol.* **37**:1049–1053. https://doi.org/10.1093/oxfordjournals.pcp.a029052

**Vogel, A., Schwacke, R., Denton, A. K., Usadel, B., Hollmann, J., Fischer, K., Bolger, A., Schmidt, M.H.-W., Bolger, M.E., Gundlach, H., Mayer, K.F.X., Weiss-Schneeweiss, H., Temsch, E.M, Krause, K.** (2018) Footprints of parasitism in the genome of the parasitic flowering plant *Cuscuta campestris*. *Nat. Commun.* **9**(1):2515. https://doi.org/10.1038/s41467-018-04344-z

**Vogt, S.H., Weyens, G., Lefèbvre, M., Bork, B., Schechert, A., and Müller, A.E.** (2014) The FLC-like gene BvFL1 is not a major regulator of vernalization response in biennial beets. *Front. Plant Sci.* **5**: 146. https://doi.org/10.3389/fpls.2014.00146

**Walter, M., Chaban, C., Schütze, K., Batistic, O., Weckermann, K., Näke, C., Blazevic, D., Grefen, C., Schumacher, K., Oecking, C., Harter, K., Kudla, J.** (2004) Visualization of protein interactions in living plant cells using bimolecular fluorescence complementation. *The Plant Journal* **40**(3):428-438. https://doi.org/10.1111/j.1365-313X.2004.02219.x

**Xing, S., van Deenen, N., Magliano, P., Frahm, L., Forestier, E., Nawrath, C., Schaller, H., Schulze Gronover, C., Prüfer, D., Poirier, Y.** (2014) ATP citrate lyase activity is post-translationally regulated by sink strength and impacts the wax, cutin and rubber biosynthetic pathways. *The Plant Journal* **79**(2):270-284. https://doi.org/10.1111/tpj.12559

**Zhang, D., Qi, J., Yue, J., Huang, J., Sun, T., Li, S., Wen, J.-F., Hettenhausen, C., Wu, J., Wang, L., Zhuang, H., Wu, J., Sun, G**. (2014) Root parasitic plant Orobanche aegyptiaca and shoot parasitic plant *Cuscuta australis* obtained Brassicaceae-specific strictosidine synthase-like genes by horizontal gene transfer. *BMC plant biology*, **14**(1):1-14. https://doi.org/10.1186/1471-2229-14-19

**Zimmermann, M.M., Grundmann, L., Känel, A., Schwarze, A., Wiedmann, D.R., Muth, J., Twyman, R.M., Prüfer, D., and Noll, G.A.** (2022) Unraveling the mystery behind the short-day-specific flowering of tobacco cultivar Maryland Mammoth. *bioRxiv*. https://doi.org/10.1101/2022.06.27.497884

## Supplementary Figures

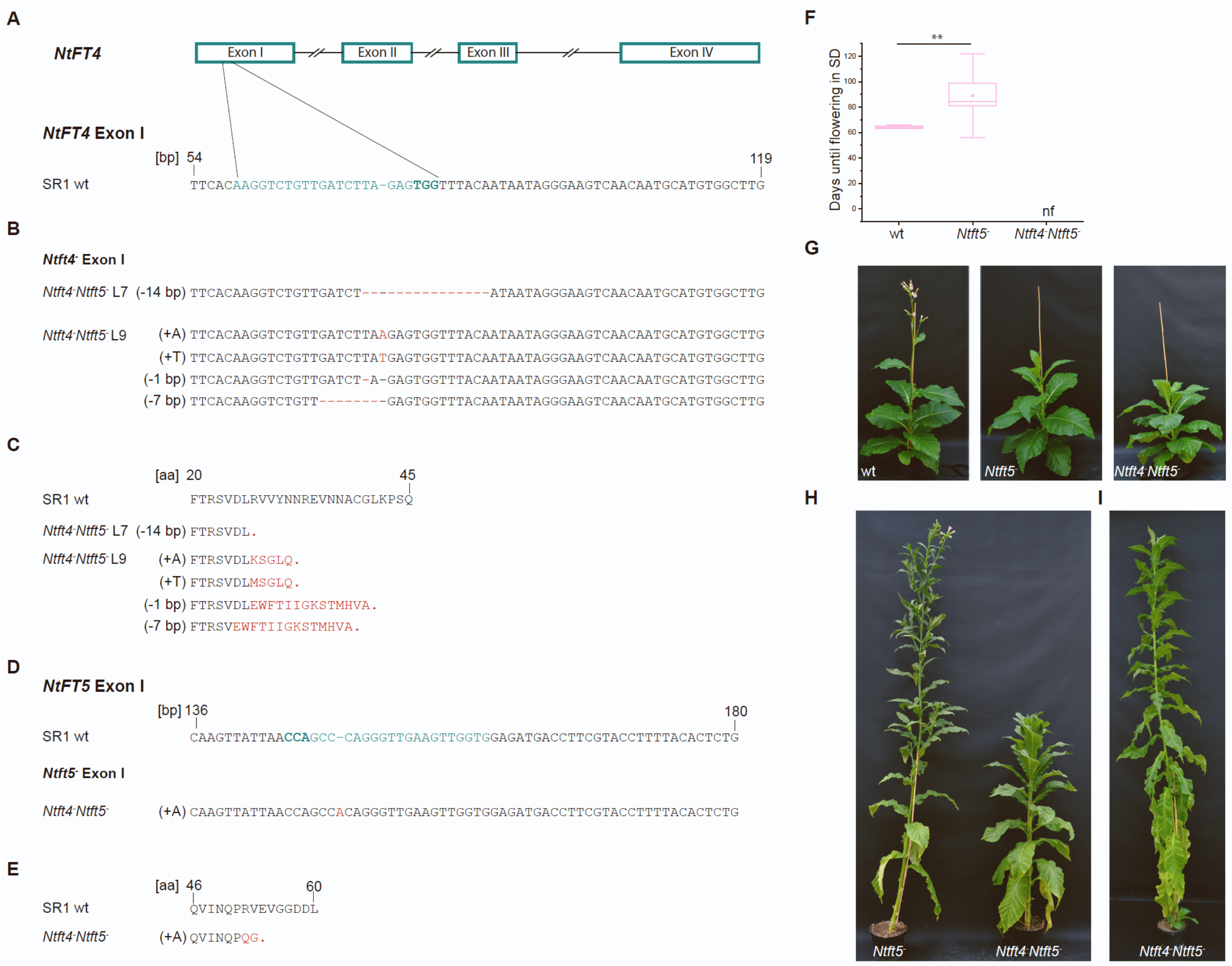


**Supplemental Figure 1. Molecular and phenotypic analysis of *Ntft4*$^{-}$*Ntft5*$^{-}$ double knockout plants. A)** Location of the *NtFT4*-specific protospacer (teal) and the protospacer adjacent motif (PAM; bold) in the 59–82 bp region of exon I of wild-type SR1 plants (SR1 WT). Exons are shown as boxes, introns as lines. **B)** PCR-based screening of two independent transgenic lines (L7, L9) for mutated allelic variants of *NtFT4*. Amplicons were subcloned and sequenced. Insertions and deletions are highlighted in red letters. L7 progeny plants possess a homozygous 14-bp deletion, whereas in L9 progeny plants the two *NtFT4* alleles each harbor one of four different mutations. **C)** Sequence alignment of native NtFT4 and the protein variants encoded by the mutated alleles. Amino acid substitutions and premature stop codons are highlighted in red letters. **D)** Knockout of NtFT5 (Schmidt et al., 2020) by a single base pair insertion at position 153 in exon I, leading to a frameshift and a premature stop codon, as validated by PCR and direct sequencing of the PCR product. **E)** Sequence alignment of native NtFT5 and the protein variant encoded by the mutated allele. Amino acid substitutions and premature stop codon are highlighted in red letters. **F)** Flowering time of SR1 wild-type plants (WT), *Ntft5*$^{-}$ plants and *Ntft4*$^{-}$*Ntft5*$^{-}$ plants. Tobacco is a day-neutral plant that produces two FT floral inducers (NtFT4 and NtFT5). NtFT5 is predominantly responsible for floral induction under LD conditions, whereas both NtFT4 and NtFT5 induce flowering under short-day (SD) conditions. Under SD conditions, SR1 wild-type plants flowered on average after 64 days, whereas *Ntft5*$^{-}$ plants, which carry homozygous knockout mutations in *NtFT5* but still express *NtFT4* under SD conditions, flowered on average after 89 days. *Ntft4*$^{-}$*Ntft5*$^{-}$ plants, lacking both floral activators, did not flower at all under SD conditions up to the end of the experiment (122 days after sowing). Days until flowering were determined when the first bud had fully opened. ** Statistically significant difference as determined using Student's *t*-test with Welch's correction, $p < 0.01$ ($n = 10$). In the boxplots, center line = median, square = mean, box limits = upper and lower quartiles, and whiskers = 1.5× interquartile range. **G)** Phenotypes of WT, *Ntft5*$^{-}$, and *Ntft4*$^{-}$*Ntft5*$^{-}$ plants cultivated under SD conditions 65 days after sowing. **H)** Phenotypes of *Ntft5*$^{-}$ and *Ntft4*$^{-}$*Ntft5*$^{-}$ plants cultivated under SD conditions 120 days after sowing. **I)** Phenotype of *Ntft4*$^{-}$*Ntft5*$^{-}$ plant cultivated under LD conditions 139 days after sowing.

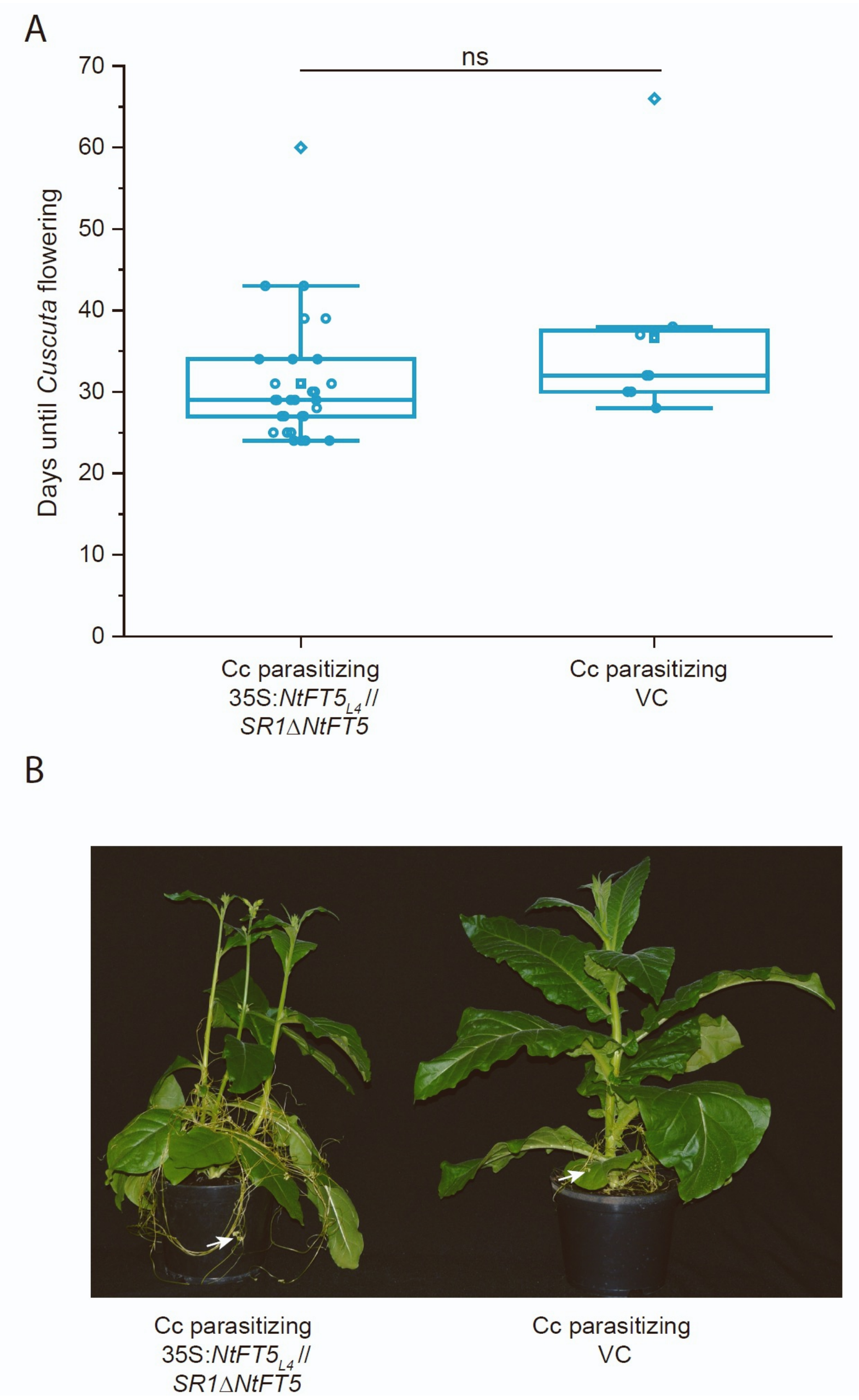


**Supplemental Figure 2. Impact of NtFT5 overexpression in the tobacco host on flowering time of *C. campestris*.** *C. campestris* (Cc) was allowed to parasitize 35S:*NtFT5*$_{L4}$//SR1Δ*NtFT5* plants, whose flowering time is reduced by approx. 20% compared to vector controls (VC) (Zimmermann et al., 2022). **A)** Elevated *NtFT5* expression levels in the host did not influence *Cuscuta* flowering time as determined by Student´s *t*-test (ns = non-significant), $n$ = 29 (35S:*NtFT5*$_{L4}$//SR1Δ*NtFT5*); $n$ = 8 (VC). In the boxplots, center line = median, square = mean, box limits = upper and lower quartiles, whiskers = 1.5× interquartile range, and diamonds = outliers. **B)** Flowering phenotype of *C. campestris* parasitizing 35S:*NtFT5*$_{L4}$//SR1Δ*NtFT5* or VC. *Cuscuta* flowers are indicated exemplarily by white arrows. The photograph was taken 79 days after tobacco seed sowing.

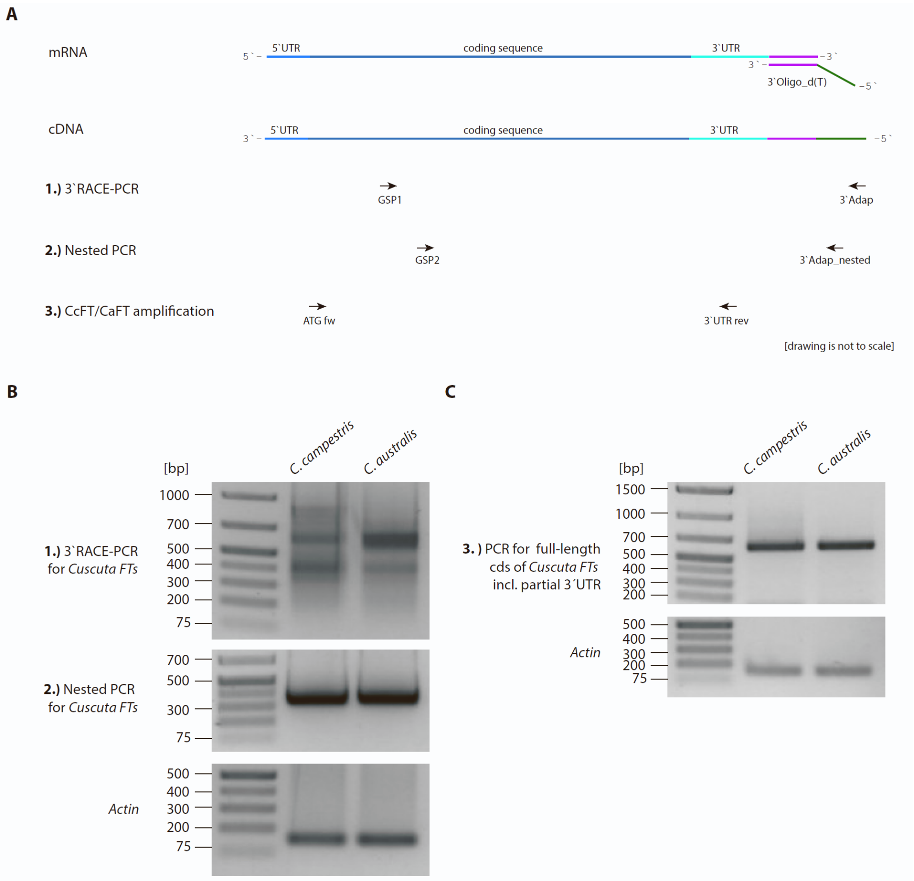


**D**

***CcFT1***

ATGGCGGCGAGGCGGCAGAGAGACGCTCTGGCTGTTGGGCGCGTGATCGGAGAGGTGCTTGAGCCTTTTACGGGCTCCGTAAACCTCAGGGTGGCTT
ACGGCGACCGCACCGTCACCAACGGCTGCGAGCTCCGGCCATCTCTCGTGCTCAACCCGCCGAGGGTCGACATCGGCGGCCATGATCTACGCACTTT
CTATACTCTCGTGATGGTGGATCCTGATGCTCCAAGTCCAAGTGATCCAAACTTAAGAGAATACTTGCACTGGTTGGTGACTGACATTCCAGCGAGC
ACCGGAGTTAATTTTGGGCATGAAATGGTATGCTATGAAAGCCCGCAACCAATCGTCGGAATCCACCGCTTCGTATTCGTACTGTTCCAGCAGATGG
GCCGACAGACCGTTTTCGCCCCGGGTTGGCGCCAAAATTTCAGCACTCGAGAATTCGCCGAGGTTCACAACCTCGGTCCACCGGTCGCGGCGATCTT
CTTTAACGGTCAACGAGAGGCGGGCACCGGCGGCAGGCGCCGTCAATAG*GAAATTCTTTGACTCCGACGTAGTCGTAGTTTATGTATGTACCGATGT*
*ACGTGTTGAGAACAATAATCCACCGTGCGCGACGGCTAGTTTCCACAATAAATATAATAATAATTCCAATTAGTG***AAAAAAAAAAAAAAAAAAAACA**
**CTGTCATGCCGTTACGTAGCG**

***CaFT1***

ATGGCGGCGAGGCGGCAGAGAGACGCTCTGGCTGTTGGGCGCGTGATCGGAGAGGTGCTTGAGCCTTTTACGGGCTCCGTAAACCTCAGGGTGGCTT
ACGGCGACCGCACCGTCACCAACGGCTGCGAGCTCCGGCCATCTCTCGTGCTCAACCCGCCGAGGGTCGACATCGGCGGCCATGATCTACGCACTTT
CTATACTCTCGTGATGGTGGATCCTGATGCTCCAAGTCCAAGTGATCCAAACTTAAGAGAATACTTGCACTGGTTGGTGACTGACATTCCAGCGAGC
ACCGGAGTTAATTTTGGGCATGAAATGGTATGCTATGAAAGCCCGCAACCAATCGTCGGAATCCACCGCTTCGTATTCGTACTGTTCCAGCAGATGG
GCCGACAGACCGTTTTCGCCCCGGGTTGGCGCCAAAATTTCAGCACTCGAGAATTCGCCGAGGTTCACAACCTCGGTCCACCGGTCGCGGCGATCTT
CTTTAACGGTCAACGAGAGGCGGGCACCGGCGGCAGGCGCCGTCAATAG*GAAATTCTTTGACTCCGACGTAGTCGTAGTTTATGTATGTACCGATGT*
*ACGTGTTGAGAACAATAATCCACCGTGCGCGGCGGCTAGTTTCCACAATAAATATAATAATAATTCCAATTAGTG***AAAAAAAAAAAAAAAAAAAAAA**
**AAACACTGTCATGCCGTTACGTAGCG**

***CcFT2***

ATGGCGGCGAGGCGGCAGAGAGACGCTCTGGCAGTTGGGCGCGTGATCGGAGAGGTGCTGGAGCCATTTACGGGCTCCATAAACCTCAGGGTGGCTT
ACGGCGATCGCACCGTCACCAACGGCTGCGAGCTCCGGCCCTCTCTCGTGCTCCACCCGCCGAGGGTCGACATCGGCGGCCATGATCTACGCACTTT
CTATACTCTCGTGATGGTGGATCCTGATGCTCCAAGTCCAAGTGATCCAAACTTAAGAGAATACTTGCACTGGTTGGTGAGTGACATTCCAGCGAGC
ACCGGAGTTAATTTTGGGCATGAGATTGTATGTTATGAAAGCCCACAGCCAATCGTCGGAATCCACCGGTTCGTATTTGTACTGTTCCAGCAGATGG
GCCGACAGACCGTTTTCGCCCCGGGTTGGCGCCAAAATTTCAGCACTCGAGAATTCGCCGAGGTTCACAACCTCGGTCCACCGGTGGCGGCGATCTT
CTTTAACGGTCAACGAGAGGCGGGCACCGGAGGCAGGCGCCGTTAA*TAGGAAATTCTTTGACTCCGACGTAGTCGTAGTTTATGTATGTACCGATGT*
*ACGTACGTATTGAGAACAATAATCCACCGTGCGC*

**Supplemental Figure 3. Identification of full-length coding sequences and 3`UTR of *Cuscuta FT* homologs.** **A)** Schematic drawing of the applied RACE-PCR strategy. GSP=gene specific primer; 3´Adap=adaptor primer; 3´Adap_nested = nested adaptor primer. The potential 5`UTR is shown in blue, coding sequence is shown in teal, 3`UTR is shown in turquoise, Poly(A)-tail and complementary Oligo d(T) are shown in purple and the adaptor sequence is shown in dark green. **B)** After cDNA synthesis using the 3`Oligo_d(T) primer with an overhanging adaptor sequence, the 3`RACE-PCR was carried out using a *CcFT1/CaFT*-specific primer (GSP1) and a primer specifically binding to the adaptor sequence (3´Adap), resulting in several unspecific bands. The PCR product was diluted 1:20 and used as a template for the second PCR using a nested *CcFT1*/*CaFT*-specific primer (GSP2) and a nested adaptor primer (3´Adap_nested), resulting in specific bands, which were purified, subcloned and sequenced. **C)** Amplification of the full-length sequences of *CcFT1*/*CaFT* and *CcFT2* was carried out using cDNA as template and primers spanning the start codon and parts of the 3`UTR. The PCR products were purified, subcloned and sequenced. **D)** Sequence of the full-length *CcFT1, CaFT* and *CcFT2* coding sequences and their 3´UTRs (partial 3´UTR in case of *CcFT2*). The coding sequences are shown in regular letters and the (partial) 3`UTR sequences are shown in italic letters. The Poly(A) tails of *CcFT1* and *CaFT* are shown in bold letters. The 3´Adap_nested primer is indicated by underlined bold letters, while the 3´UTR primer is indicated by underlined italic letters.

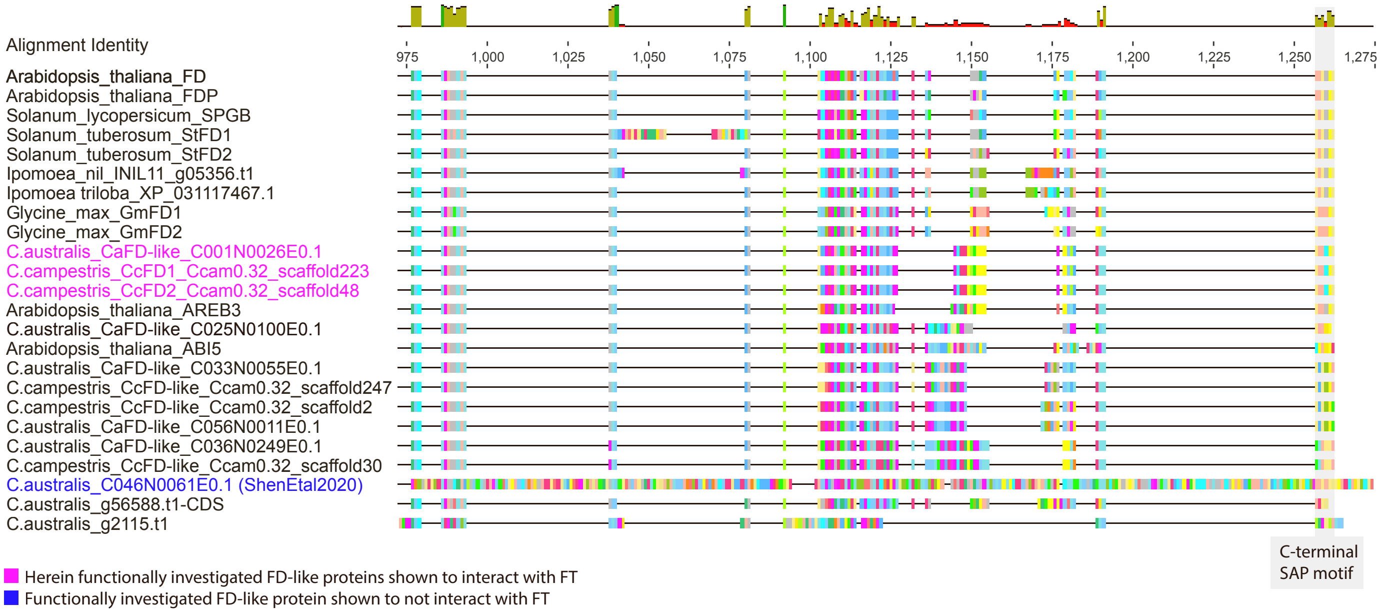


**Supplemental Figure 4. SAP-motif region of FD-like proteins.** Visualization of aligned sequences at the C-terminus of FD-like proteins, showing the presence of a SAP-like motif in the newly identified and functionally tested CcFD1, CcFD2 and CaFD-like genes (magenta), for which we showed an interaction with endogenous *Cuscuta* FT proteins. Within the alignment block, same-colored boxes indicate the same amino acids. For comparison, functionally validated FD and FD-related proteins from *Arabidopsis thaliana* and other angiosperms are included. Highlighted in blue is the FD protein sequence reported not to interact with endogenous *Cuscuta* FT (Shen et al., 2020). The complete alignment is published in reusable Nexus format in the DRYAD Data Repository, https://datadryad.org/stash/share/DK8Olh2VqFwbGNL0GtkGt24dD0GhWhJn82oLBC1XK70

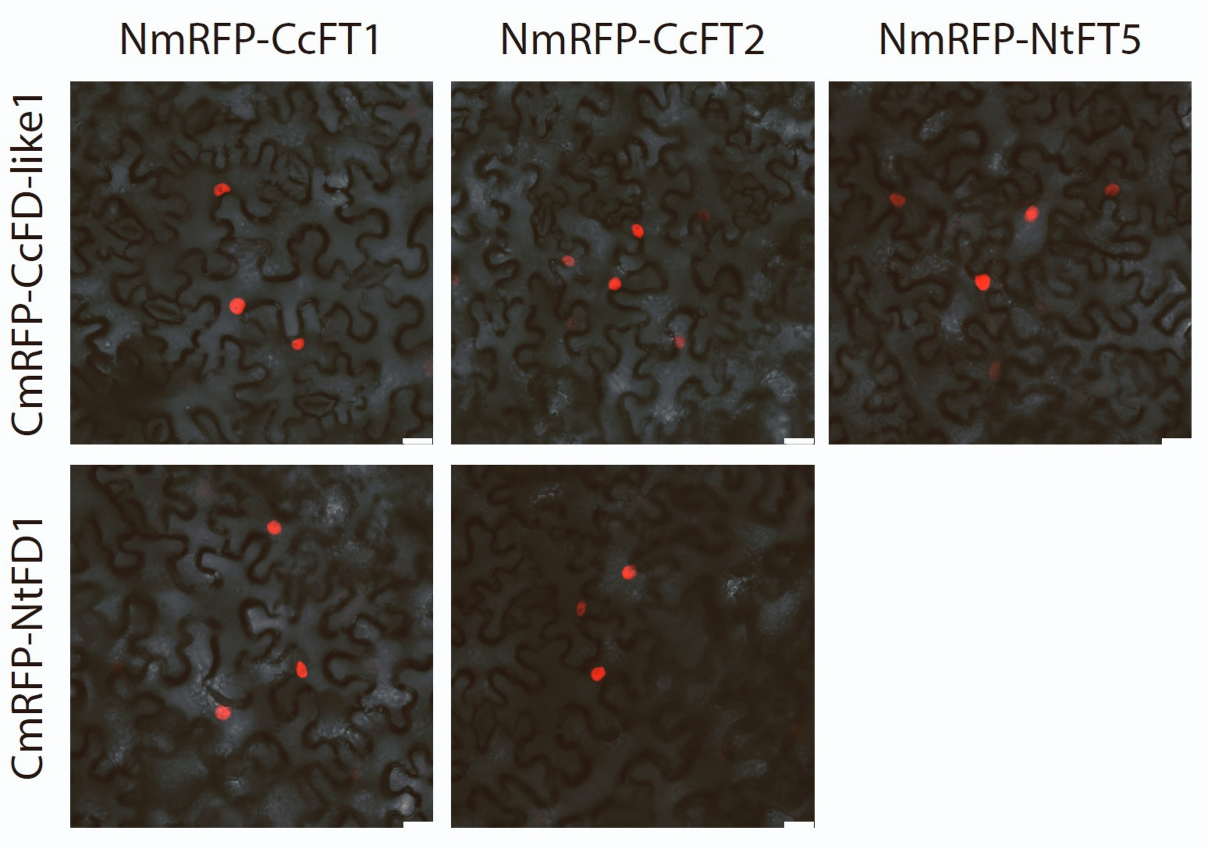


**Supplemental Figure 5. Bimolecular fluorescence complementation assay in *Nicotiana benthamiana* epidermal cells with *Cuscuta* and tobacco FD/FT homologs.** The FD homologs were C-terminally fused to the C-terminal mRFP1 fragment, and the FT homologs were C-terminally fused to the N-terminal mRFP1 fragment. Interaction was confirmed by reconstituted mRFP fluorescence visible in the nuclei. N-terminal or C-terminal mRFP1 fragments served as negative controls and were co-expressed with the corresponding FD or FT fusion proteins. We did not observe any nonspecific interactions in any combination. Scale bar = 20 μm.

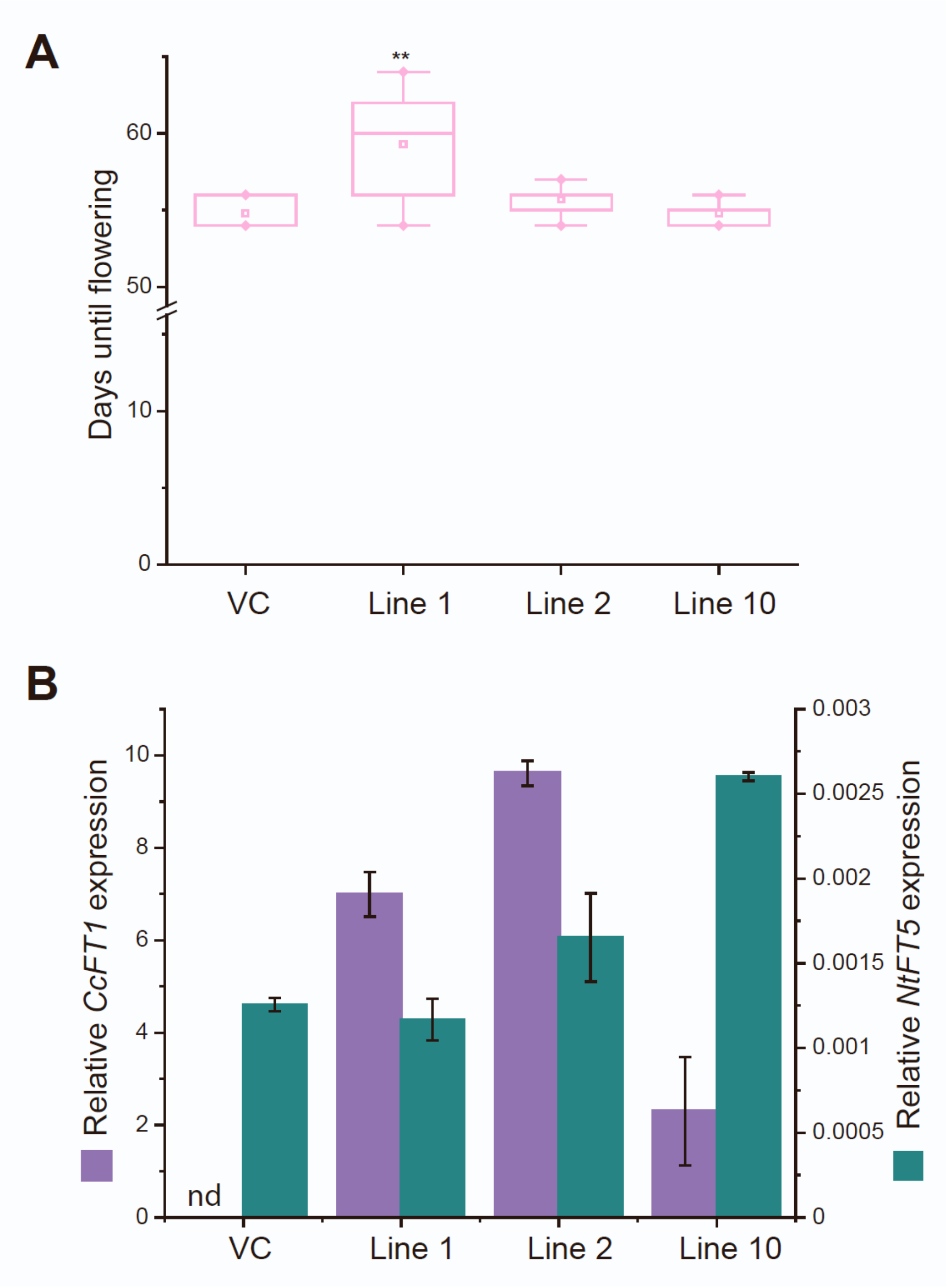


**Supplemental Figure 6. Flowering time analysis of tobacco plants overexpressing *CcFT1*. A)** Flowering phenotype of tobacco lines 1, 2, and 10 overexpressing *CcFT1* compared to vector controls (VC). Days until flowering were determined when the first bud had fully opened. **Statistically significant difference as determined by ANOVA and Tukey's *post hoc* test, p <0.01; *n* = 10 (lines 1, 2 and 10), *n* = 5 (VC). In the boxplots, center line = median, square = mean, box limits = upper and lower quartiles, whiskers = 1.5× interquartile range, and diamonds = outliers. **B)** Overexpression levels of *CcFT1* and endogenous expression levels of *NtFT5* in transgenic plants were determined when plants entered the reproductive growth phase. No *CcFT1* expression was detected in vector controls (nd). Data are means ± SEM, *n* = 3 biological replicates.

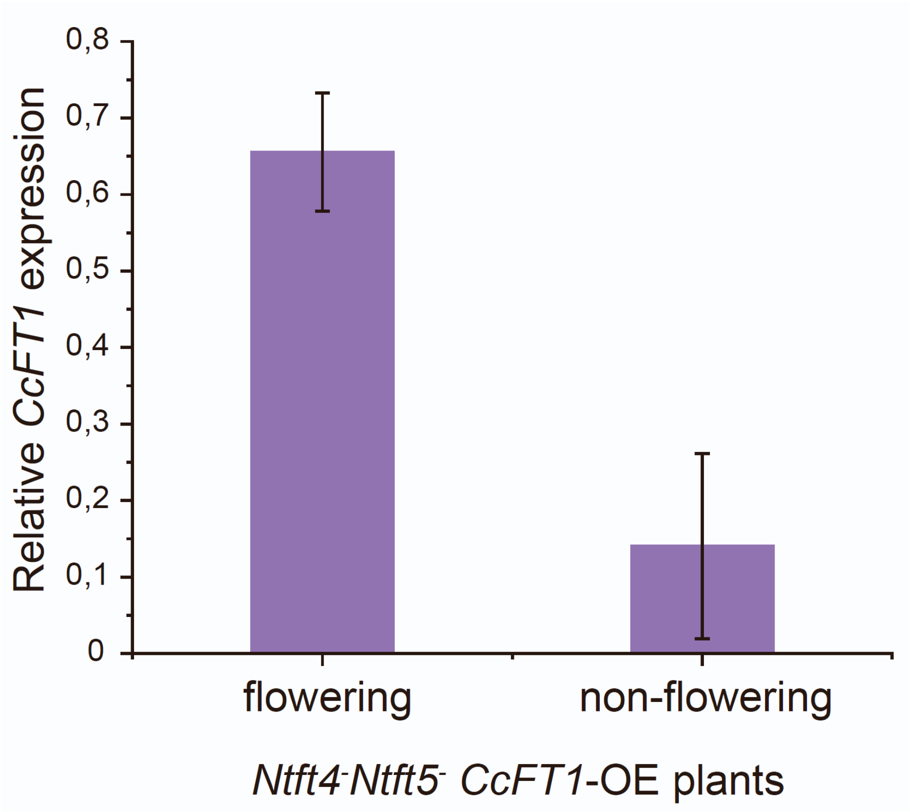


**Supplemental Figure 7. Relative expression levels of *CcFT1* in flowering versus non-flowering *Ntft4⁻Ntft5⁻* plants overexpressing *CcFT1*.** Data are means ± SEM. *n* = 46 (flowering), *n* = 2 (non-flowering).

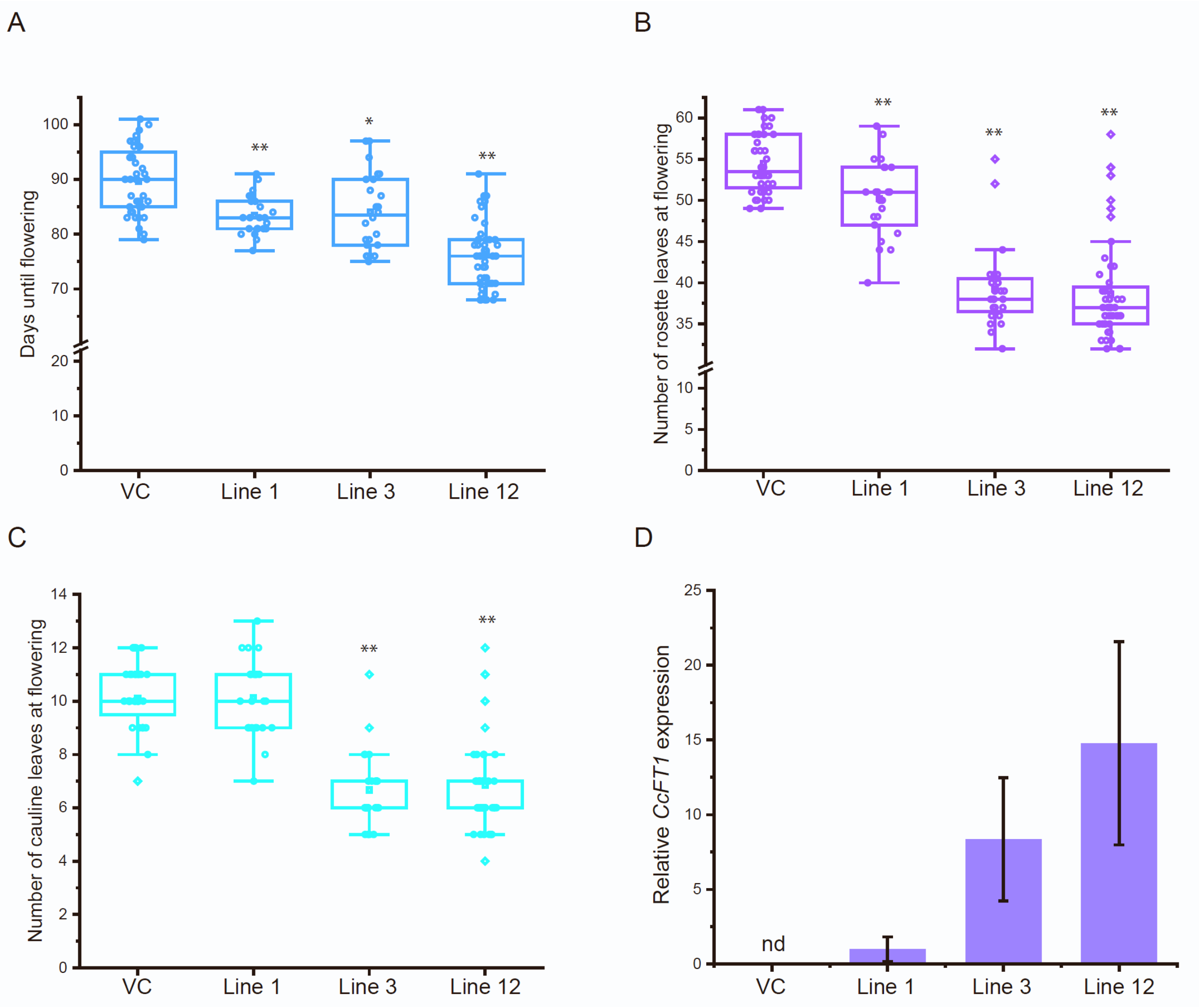


**Supplemental Figure 8 Phenotypic analysis of *CcFT1* overexpressing *A. thaliana* Col-0 plants grown under SD conditions. A)** Flowering phenotype of overexpression lines 1, 3, and 12 compared to vector controls (VC). Days until flowering were determined when the first bud had fully opened. **B)** Numbers of rosette leaves and **C)** cauline leaves were counted when the first bud had fully opened. *n* = 23 (line 1), *n* = 24 (line 3) and *n* = 48 (line 12), *n* = 40 (VC). In the boxplots, center line = median, square = mean, box limits = upper and lower quartiles, whiskers = 1.5× interquartile range, and diamonds = outliers. **D)** Overexpression levels of *CcFT1* in transgenic plants and vector controls were determined in seedlings. No *CcFT1* expression was detected in vector controls (nd). Data are means ± SEM, n ≥ 8 biological replicates. Statistically significant differences compared to vector controls was determined by Welch´s ANOVA and Games-Howell *post hoc* test for (A) and ANOVA and Tuckey´s *post hoc* test for (B-D), with *p = <0.05, **p <0.01.

## Supplementary Tables

**Supplemental Table 1. RNA Seq transcript levels.**

| | | *Cuscuta campestris* total reads mapped: 1 990 329 102 (after trim) | | *Cuscuta australis* total reads mapped: 908 853 342 (after trim) | |
|---|---|---|---|---|---|
| | **Gene length** | **Coverage** | **RPKM** | **Coverage** | **RPKM** |
| FT1 | 534 bp | 4.56 | 57.01 | 12.98 | 633.80 |
| FT2[1] | 531 bp | 4.68 | 64.81 | -- | -- |
| FD-like | 918 bp | 1 025.49 | 11 596.03 | 398.02 | 16 046.96 |
| SOC1 | 714 bp | 43 406.26 | 498 893.10 | 6 731.04 | 297 048.13 |
| TFL1 | 594 bp | 1 724.57 | 22 286.12 | 5 020.35 | 226 723.48 |
| LFY | 1263 bp | 236.22 | 2 592.57 | 313.55 | 13 852.78 |
| CO-like | 1047 bp | 3 986.97 | 50 309.32 | 2 676.82 | 123 982.11 |
| GI-like | 3453 bp | 8 752.11 | 97 400.03 | 1 981.41 | 79 456.96 |

**Supplemental Table 2. RNA-Seq data for *Cuscuta campestris* and *C. australis*.**

| Organism | Tissue | SRA accession | Illumina read length, mode | Bases (Gbp) |
|---|---|---|---|---|
| *C. australis* | Stem | SRR19610423 | 151 bp, PE | 6,445 |
| *C. australis* | Stem | SRR19611357 | 151 bp, PE | 6,505 |
| *C. australis* | Stem | SRR19611520 | 151 bp, PE | 6,386 |
| *C. australis* | Stem | SRR19611521 | 151 bp, PE | 5,863 |
| *C. australis* | Stem | SRR19612874 | 151 bp, PE | 5,699 |
| *C. australis* | Stem | SRR19612875 | 151 bp, PE | 6,616 |
| *C. australis* | Stem | SRR19612878 | 151 bp, PE | 6,220 |
| *C. australis* | Stem | SRR19627926 | 151 bp, PE | 6,231 |
| *C. australis* | Stem | SRR19627939 | 151 bp, PE | 6,261 |
| *C. australis* | Stem | SRR20661662 | 151 bp, PE | 6,186 |
| *C. australis* | Stem | SRR20662442 | 151 bp, PE | 6,547 |
| *C. australis* | Stem | SRR20662443 | 151 bp, PE | 5,744 |
| *C. australis* | Germinating seedling | SRR6664647 | 151 bp, PE | 6,087 |
| *C. australis* | Haustoria on tomato | SRR6664648 | 151 bp, PE | 7,236 |
| *C. australis* | Bud | SRR6664649 | 151 bp, PE | 8,374 |
| *C. australis* | Ovary | SRR6664650 | 151 bp, PE | 8,936 |
| *C. australis* | Stem tip on tomato | SRR6664651 | 151 bp, PE | 7,697 |
| *C. australis* | Seeds | SRR6664652 | 151 bp, PE | 7,329 |
| *C. australis* | Pre-haustoria on tomato | SRR6664653 | 151 bp, PE | 8,503 |
| *C. australis* | Stems on tomato | SRR6664654 | 151 bp, PE | 7,452 |
| | | | | **136,328** |

| | | | | |
|---|---|---|---|---|
| *C. campestris* | Not specified* | ERR1916345 | 125 bp, PE | 5,062 |
| *C. campestris* | Not specified* | ERR1916346 | 125 bp, PE | 1,832 |
| *C. campestris* | Not specified* | ERR1916347 | 125 bp, PE | 8,524 |
| *C. campestris* | Not specified* | ERR1916348 | 125 bp, PE | 20,608 |
| *C. campestris* | Not specified* | ERR1916349 | 125 bp, PE | 17,925 |
| *C. campestris* | Not specified* | ERR1916350 | 125 bp, PE | 7,865 |
| *C. campestris* | Not specified* | ERR1916351 | 125 bp, PE | 8,375 |
| *C. campestris* | Not specified* | ERR1916352 | 125 bp, PE | 8,777 |
| *C. campestris* | Not specified* | ERR1916353 | 125 bp, PE | 5,125 |
| *C. campestris* | Not specified* | ERR1916354 | 125 bp, PE | 2,518 |
| *C. campestris* | Not specified* | ERR1916355 | 125 bp, PE | 14,034 |
| *C. campestris* | Not specified* | ERR1916356 | 125 bp, PE | 6,807 |
| *C. campestris* | Not specified* | ERR1916357 | 125 bp, PE | 0.541 |
| *C. campestris* | Not specified* | ERR1916358 | 125 bp, PE | 24,504 |
| *C. campestris* | Not specified* | ERR1916359 | 125 bp, PE | 6,649 |
| *C. campestris* | Not specified* | ERR1916360 | 125 bp, PE | 1,202 |
| *C. campestris* | Not specified* | ERR1916361 | 125 bp, PE | 4,443 |
| *C. campestris* | Not specified* | ERR1916362 | 125 bp, PE | 13,772 |
| *C. campestris* | Not specified* | ERR1916363 | 125 bp, PE | 5,153 |
| *C. campestris* | Not specified* | ERR1916364 | 125 bp, PE | 3,823 |
| *C. campestris* | Penetrating haustorium | SRR12763776 | 101 bp, PE | 3,121 |
| *C. campestris* | Attaching haustorium | SRR12763777 | 101 bp, PE | 3,094 |
| *C. campestris* | Attaching haustorium | SRR12763778 | 101 bp, PE | 3,175 |
| *C. campestris* | Attaching haustorium | SRR12763779 | 101 bp, PE | 3,168 |
| *C. campestris* | Swelling haustorium | SRR12763780 | 101 bp, PE | 3,181 |
| *C. campestris* | Swelling haustorium | SRR12763781 | 101 bp, PE | 3,194 |
| *C. campestris* | Swelling haustorium | SRR12763782 | 101 bp, PE | 3,187 |
| *C. campestris* | Vegetative stem | SRR12763783 | 101 bp, PE | 3,176 |
| *C. campestris* | Penetrating haustorium | SRR12763787 | 101 bp, PE | 3,130 |
| *C. campestris* | Penetrating haustorium | SRR12763788 | 101 bp, PE | 3,204 |
| *C. campestris* | Vegetative stem | SRR12763789 | 101 bp, PE | 3,119 |
| *C. campestris* | Vegetative stem | SRR12763790 | 101 bp, PE | 3,095 |
| *C. campestris* | Haustorial region | SRR13299802 | 51 bp, SE | 0.387 |
| *C. campestris* | Haustorial region | SRR13299801 | 51 bp, SE | 0.396 |
| *C. campestris* | Haustorial region | SRR13299800 | 51 bp, SE | 0.618 |

| | | | | |
|---|---|---|---|---|
| *C. campestris* | Haustorial region | SRR13299799 | 51 bp, SE | 0.714 |
| *C. campestris* | Haustorial region | SRR13299798 | 51 bp, SE | 0.172 |
| *C. campestris* | Haustorial region | SRR13299797 | 51 bp, SE | 0.301 |
| *C. campestris* | Haustorial region | SRR13299796 | 51 bp, SE | 0.212 |
| *C. campestris* | Haustorial region | SRR13299795 | 51 bp, SE | 0.308 |
| *C. campestris* | Haustorial region | SRR13299794 | 51 bp, SE | 0.225 |
| *C. campestris* | Haustorial region | SRR13299793 | 51 bp, SE | 0.283 |
| *C. campestris* | Haustorial region | SRR14853881 | 151 bp, PE | 8,619 |
| *C. campestris* | Haustorial region | SRR14853883 | 151 bp, PE | 8,954 |
| *C. campestris* | Haustorial region | SRR14853885 | 151 bp, PE | 11,372 |
| *C. campestris* | Haustorial region | SRR14853887 | 151 bp, PE | 9,394 |
| | | | | **247,357** |

* Vogel et al. (2018) used "non-infectious feeding stems, detached starving stems and different infection stages", p.8; PE – paired-end mode; SE – single-end mode.

**Supplemental Table 3. Taxon list and accession numbers for FD-like proteins used in gene family analysis.**

| Species | Gene denomination (if any) | Locus tag, accession number |
|---|---|---|
| *Arabidopsis thaliana* | ABI5 | AT2G36270 |
| *Arabidopsis thaliana* | AREB3 | AT3G56850 |
| *Arabidopsis thaliana* | FD | AT4G35900 |
| *Arabidopsis thaliana* | FDP (BZIP27) | AT2G17770 |
| *Brachypodium distachyon* | BdFD1 | Bradi3g60870 |
| *Brachypodium distachyon* | BdFD2 | Bradi4g36587 |
| *Brachypodium distachyon* | BdFD3 | Bradi1g29920 |
| *Cuscuta australis* | BZIP-family gene | g21250.t1 |
| *Cuscuta australis* | BZIP-family gene | g56588.t1-CDS |
| *Cuscuta australis* | BZIP-family gene | g2115.t1 |
| *Cuscuta australis* | BZIP-family gene | g29401.t1 |
| *Cuscuta australis* | *CaFD | *C046N0061E0.1 |
| *Cuscuta australis* | CaFD-like | C001N0026E0.1; |
| *Cuscuta australis* | CaFD-like | C025N0100E0.1 |
| *Cuscuta australis* | CaFD-like | C033N0055E0.1 |
| *Cuscuta australis* | CaFD-like | C056N0011E0.1 |
| *Cuscuta australis* | CaFD-like | C036N0249E0.1 |
| *Cuscuta campestris* | CcFD-like | Ccam0.32, scaffold247, Cc032062 |
| *Cuscuta campestris* | CcFD-like | Ccam0.32, scaffold2, Cc010070 |
| *Cuscuta campestris* | CcFD-like | Ccam0.32, scaffold30, Cc002951 |
| *Cuscuta campestris* | FD-like | Ccam0.32, scaffold218, Cc030291 |
| *Cuscuta campestris* | CcFD1 | Ccam0.32, scaffold223, Cc030608 |
| *Cuscuta campestris* | CcFD2 | Ccam0.32, scaffold48, OOIL02004257.1 |
| *Fragaria vesca* | FvFD1 | mrna14556.1-v1.0-hybrid |
| *Fragaria vesca* | FvFD2 | mrna08556.1-v1.0-hybrid |
| *Glycine max* | GmFD1 | Glyma04g02420 |
| *Glycine max* | GmFD2 | Glyma06g02470 |
| *Hordeum vulgare* | HvFD1 | BAJ91167 |
| *Hordeum vulgare* | HvFD2 | BAK04622 |
| *Hordeum vulgare* | HvFD3 | AK249012 |
| *Ipomoea nil* | INIL11 | g05356.t1 |
| *Ipomoea triloba* | ItFD | XP_31117467.1 |
| *Malus domestica* | MdFD1 | MDP0000169473 |
| *Malus domestica* | MdFD2 | MDP0000636541 |
| *Musa acuminata* | MaFD1 | GSMUA_Achr1T02630 |
| *Musa acuminata* | MaFD2 | GSMUA_Achr5T11470_001 |
| *Musa acuminata* | MaFD3 | GSMUA_chr9G24090_001 |
| *Oryza sativa* | n.a. | Os01g0813100 |
| *Oryza sativa* | OsFD1 | Os09g0540800 |
| *Oryza sativa* | OsFD2 | Os06g0720900 |
| *Oryza sativa* | OsFD3 | Os02g0833600 |

| | | |
|---|---|---|
| *Oryza sativa* | OsFD4 | Os08g0549600 |
| *Oryza sativa* | OsFD5 | Os06g0724000 |
| *Oryza sativa* | OsFD6 | Os06g0719500 |
| *Oryza sativa* | TRAB1 | Os08g0472000 |
| *Phoenix dactylifera* | PdFD1 | PDK30s1175071g003 |
| *Piecea glauca* | PgABI5-1 | BT102312.1 |
| *Piecea glauca* | PgABI5-2 | BT110053.1 |
| *Pinus taeda* | PtABI5 | AEK86263.1 |
| *Popolus trichocarpa* | PtFD1 | POPTR_0005s11140.1 |
| *Popolus trichocarpa* | PtFD2 | POPTR_0005s26480 |
| *Setaria italica* | SiFD1 | Si031077m |
| *Setaria italica* | SiFD2 | Si007412m |
| *Setaria italica* | SiFD3 | Si023448m |
| *Setaria italica* | SiFD4 | Si014546m |
| *Solanum lycopersicum* | SlFD2 | Solyc02g061990.2.1 |
| *Solanum lycopersicum* | SPGB | Solyc02g083520 |
| *Solanum tuberosum* | StFD1 | Sotub02g026810 |
| *Solanum tuberosum* | StFD2 | Sotub02g009830 |
| *Sorghum bicolor* | SbFD1 | Sb02g031340 |
| *Sorghum bicolor* | SbFD2 | Sb10g030400 |
| *Sorghum bicolor* | SbFD3 | Sb04g038600 |
| *Triticum aestivum* | TaFD1 | CK206464 |
| *Triticum aestivum* | TaFDL13 | ABZ91908 |
| *Triticum aestivum* | TaFDL15 | ABZ91909 |
| *Triticum aestivum* | TaFDL2 | ABZ91911 |
| *Triticum aestivum* | TaFDL3 | ABZ91912 |
| *Vitis vinifera* | VvFD1 | GSVIVT01009970001 |
| *Vitis vinifera* | VvFD2 | GSVIVT01006332001 |
| *Zea mays* | DLF1 | GRMZM2G067921 |
| *Zea mays* | ZmFD2 | GRMZM2G402862 |
| *Zea mays* | ZmFD3 | GRMZM2G073892 |

*CaFD sequence identified by Shen et al. (2020).

**Supplemental Table 4. Taxon list and accession numbers of PEBP proteins for phylogenetic inference.**

| Species | Gene denomination (if any) | Locus tag, accession number |
|---|---|---|
| *Aegilops tauschii* | FT | ABI34864.1 |
| *Antirrhinum majus* | CENTRORADIALIS-like | CAC21563.1 |
| *Antirrhinum majus* | CENTRORADIALIS-like | CAC21564.1 |
| *Aquilegia formosa* | TERMINAL FLOWER 0 | ABB90591.1 |
| *Arabidopsis lyrata* subsp. *lyrata* | CENTRORADIALIS-like | XP_2876387.1 |
| *Arabidopsis lyrata* subsp. *lyrata* | TERMINAL FLOWER 0 | AAM27957.1 |
| *Arabidopsis lyrata* subsp. *lyrata* | TERMINAL FLOWER 1 | XP_2873117.1 |
| *Arabidopsis lyrata* subsp. *lyrata* | BROTHER of FT and TFL 1 | XP_2864769.1 |
| *Arabidopsis thaliana* | PEBP family protein | NP_176726.1 |
| *Arabidopsis thaliana* | PEBP family protein | NP_193770.1 |
| *Arabidopsis thaliana* | CENTRORADIALIS-like | NP_180324.1 |
| *Arabidopsis thaliana* | PEBP family protein | NP_196004.1 |
| *Arabidopsis thaliana* | PEBP family protein | NP_201010.1 |
| *Arabidopsis thaliana* | PEBP family protein | NP_173250.1 |
| *Beta vulgaris* | flowering locus T-like protein FT2 | ADM92610.1 |
| *Beta vulgaris* | flowering locus T-like protein FT1 | ADM92608.1 |
| *Beta vulgaris* | centroradialis-like protein CEN1 | ADM92611.1 |
| *Brassica napus* | BNTFL1-1 | BAA33415.1 |
| *Brassica napus* | BNTFL1-3 | BAA33417.1 |
| *Brassica napus* | BNTFL1-2 | BAA33416.1 |
| *Brassica rapa* | BRTFL1-1 | BAA33418.1 |
| *Brassica rapa* | BRTFL1-2 | BAA33419.1 |
| *Capsicum annuum* | self-pruning-like protein | AAZ66798.1 |
| *Capsicum frutescens* | fasciculate | ACL27223.1 |
| *Carica papaya* | flowering locus T | ACX85427.1 |
| *Chaenomeles sinensis* | TFL1-like protein | BAD10965.1 |
| *Chaenomeles sinensis* | TFL1-like protein | BAD10971.1 |
| *Chrysanthemum lavandulifolium* | flowering locus T-like protein | ACY82397.2 |
| *Chrysanthemum* x *morifolium* | flowering locus T-like protein | ACX48949.1 |
| *Chrysanthemum* x *morifolium* | flowering locus T-Like protein | BAJ14266.2 |
| *Citrus sinensis* | terminal flower | AAR04683.1 |
| *Citrus sinensis* | terminal flower | AAR04684.1 |
| *Citrus trifoliata* | CTRSFT1-like protein | ABY91244.1 |
| *Citrus trifoliata* | CTRSTFL-like protein | ABY91242.1 |
| *Citrus trifoliata* | CTRSTFL-like protein | ABY91243.1 |
| *Citrus unshiu* | CiFT | BAA77836.1 |
| *Citrus unshiu* | flowering locus T | BAF96644.1 |
| *Citrus unshiu* | flowering locus T | BAF96645.1 |
| *Citrus unshiu* | MOTHER of FT, TFL1-like protein | BAF93494.1 |
| *Crocus sativus* | terminal flower 1-like protein | ACX53295.1 |
| *Cucumis sativus* | FT-like protein | BAH28253.1 |
| *Cucumis sativus* | TFL1-like protein | BAH28255.1 |
| *Cucurbita maxima* | flowering locus T-like 2 | ABI94606.1 |
| *Cucurbita maxima* | flowering locus T-like 1 | ABI94605.1 |
| *Cucurbita moschata* | FTL2 | ABR20499.1 |
| *Cucurbita moschata* | FTL1 | ABR20498.1 |
| *Cuscuta australis* | FT, partial | Data Dryad |
| *Cuscuta australis* | C020N0378E0.1 | CaFT |
| *Cuscuta campestris* | CcFT1 | Data Dryad |
| *Cuscuta campestris* | CcFT2 | Data Dryad |

| | | |
|---|---|---|
| *Cuscuta campestris* | TL1-like partial | Data Dryad |
| *Cydonia oblonga* | TFL1-like protein | BAD10964.1 |
| *Cydonia oblonga* | TFL1-like protein | BAD10970.1 |
| *Erythrante guttata* | FT, partial | Data Dryad |
| *Ficus carica* | flowering locus T-like protein | BAI60052.1 |
| *Glycine max* | Dt1 | ADF30893.1 |
| *Glycine max* | brother of FT and TFL1 protein | ABS57463.1 |
| *Glycine max* | CETS1 | ABF65987.1 |
| *Glycine max* | mother of FT-like protein | ACA24491.1 |
| *Glycine max* | unknown | ACU14433.1 |
| *Gossypium arboreum* | terminal flower 1a | ABW24961.1 |
| *Gossypium arboreum* | terminal flower 1b | ABW24962.1 |
| *Gossypium arboreum* | terminal flower 1b | ABW24969.1 |
| *Gossypium hirsutum* | terminal flower 1a | ABW24963.1 |
| *Gossypium hirsutum* | terminal flower 1a | ABW24970.1 |
| *Gossypium hirsutum* | terminal flower 1a | ABW24967.1 |
| *Gossypium hirsutum* | terminal flower 1b | ABY62770.1 |
| *Gossypium hirsutum* | terminal flower 1b | ABW24964.1 |
| *Gossypium raimondii* | terminal flower 1a | ABW24965.1 |
| *Gossypium raimondii* | terminal flower 1b | ABW24966.1 |
| *Helianthus annuus* | flowering locus T4 | ADF32945.1 |
| *Helianthus annuus* | flowering locus T2 | ADF32947.1 |
| *Helianthus annuus* | terminal flower 1 | ADO61015.1 |
| *Hordeum vulgare* | FT | ABI55201.1 |
| *Hordeum vulgare* | FT | ABI55202.1 |
| *Hordeum vulgare* | FT | ABJ97441.1 |
| *Hordeum vulgare* subsp. *vulgare* | vrn-H3 late flowering allele | ABK91684.1 |
| *Hordeum vulgare* subsp. *vulgare* | FT-like protein | ABB99414.1 |
| *Hordeum vulgare* subsp. *vulgare* | terminal flower 1-like protein | ABF85670.1 |
| *Hordeum vulgare* subsp. *vulgare* | homologous protein to TFL1 | BAH24197.1 |
| *Impatiens balsamina* | TERMINAL FLOWER 1 protein | CAI61980.1 |
| *Impatiens balsamina* | TERMINAL FLOWER 1 protein | CAI61982.1 |
| *Ipomoea nil* | FT-like protein | ABW73563.1 |
| *Ipomoea nil* | FT-like protein | ABW73562.1 |
| *Ipomoea nil* | CENTRORADIALIS homolog | BAE44112.1 |
| *Ipomoea triloba* | FT, partial | Data Dryad |
| *Lactuca sativa* | FT, partial | Data Dryad |
| *Leavenworthia crassa* | terminal flower 1-like protein | ADC32542.1 |
| *Lolium perenne* | FT3 | ABC33722.1 |
| *Lolium perenne* | terminal flower 1-like protein | AAG31808.1 |
| *Lotus japonicus* | CEN/TFL1-like protein | AAQ93599.1 |
| *Malus domestica* | flowering locus T | ACL98164.1 |
| *Malus domestica* | flowering locus T like protein | BAD08340.1 |
| *Malus domestica* | CENTRORADIALIS like protein | BAG31957.1 |
| *Malus domestica* | CENTRORADIALIS like protein | BAG31958.1 |
| *Malus domestica* | TFL1-like protein | BAD06418.1 |
| *Malus domestica* | TFL1-like protein | BAD10961.1 |
| *Malus domestica* | TFL1-like protein | BAD10967.1 |
| *Malus domestica* | TFL1 like protein | BAG31959.1 |
| *Medicago truncatula* | PEBP | ABE80135.1 |
| *Misopates orontium* | CEN/TFL1-like protein | CAJ44126.1 |
| *Nicotiana tabacum* | NtFT5 | Data Dryad |
| *Nicotiana tabacum* | NtFT4 | Data Dryad |

| | | |
|---|---|---|
| *Nicotiana tabacum* | CEN-like protein 2 | AAD43529.1 |
| *Nicotiana tabacum* | CEN-like protein 4 | AAD43530.1 |
| *Nicotiana tabacum* | CEN-like protein 1 | AAD43528.1 |
| *Olea europaea* | FT, partial | Data Dryad |
| *Oncidium* hybrid cultivar | flowering locus T | ACC59806.1 |
| *Oryza glumipatula* | FT-like protein | BAH56285.1 |
| *Oryza longistaminata* | FT-like protein | BAH30248.1 |
| *Oryza rufipogon* | Hd3a | BAG72302.1 |
| *Oryza rufipogon* | FT-like protein | BAH30250.1 |
| *Oxybasis rubra* | Flowering locus T-like 1 protein | ABV56568.1 |
| *Oxybasis rubra* | flowering locus T-like 2 | ABP02016.1 |
| *Phyllostachys meyeri* | FT-like protein | BAI49899.1 |
| *Phyllostachys meyeri* | FT-like protein | BAI49900.1 |
| *Phyllostachys meyeri* | FT-like protein | BAI49901.1 |
| *Picea abies* | FT-like/TFL1-like protein | ABQ85553.1 |
| *Picea sitchensis* | unknown | ABK25607.1 |
| *Picea sitchensis* | unknown | ACN40175.1 |
| *Pisum sativum* | TFL1a | AAR03725.1 |
| *Populus deltoides* | flowering locus T-like protein FT1 | AAS00056.1 |
| *Populus nigra* | flowering locus T | BAD01561.1 |
| *Populus nigra* | flowering locus T | BAD01612.1 |
| *Populus nigra* | flowering locus T | BAD02371.1 |
| *Populus nigra* | FLOWERING LOCUS T | BAG12904.1 |
| *Populus nigra* | terminal flower 1 | BAD22599.1 |
| *Populus nigra* | flowering locus T like protein | BAD22601.1 |
| *Populus nigra* | flowering locus T like protein | BAD27481.1 |
| *Populus nigra* | flowering locus T like protein | BAD22677.1 |
| *Populus tremula* | flowering locus T-like protein FT1 | ABD52003.1 |
| *Populus trichocarpa* | protein HEADING DATE 3A | XP_2316173.1 |
| *Populus trichocarpa* | protein HEADING DATE 3A | XP_2311264.1 |
| *Populus trichocarpa* | CEN-like protein 2 | XP_2312811.1 |
| *Populus trichocarpa* | CEN-like protein 1 | XP_2321903.2 |
| *Populus trichocarpa* | MFT-like protein | ABC26020.1 |
| *Prunus mume* | flowering locus T like protein | CAQ16124.1 |
| *Prunus mume* | TFL1-like protein | BAJ14521.1 |
| *Prunus persica* | flower locus T | ACH73165.1 |
| *Pyrus communis* | TFL1-like protein | BAD10963.1 |
| *Pyrus communis* | TFL1-like protein | BAD10969.1 |
| *Pyrus pyrifolia* | TFL1-like protein | BAD10962.1 |
| *Pyrus pyrifolia* | TFL1-like protein | BAD10968.1 |
| *Raphanus sativus* | terminal flower1-like protein | BAH37010.1 |
| *Rhaphiolepis bibas* | TFL1-like protein | BAD10966.1 |
| *Rhaphiolepis bibas* | TFL1-like protein | BAD10972.1 |
| *Sinapis alba* | flowering locus T | ACM69283.1 |
| *Solanum lycopersicum* | self-pruning protein | AAC26161.1 |
| *Solanum tuberosum* | terminal flower 1 protein | ABC24691.1 |
| *Sorghum bicolor* | protein HEADING DATE 3A | XP_2436509.1 |
| *Sorghum bicolor* | protein TWIN SISTER of FT | XP_2443085.1 |
| *Sorghum bicolor* | protein FLOWERING LOCUS T | XP_2454134.1 |
| *Sorghum bicolor* | CEN-like protein 2 | XP_2442808.1 |
| *Sorghum bicolor* | CEN-like protein 2 | XP_2450283.1 |
| *Sorghum bicolor* | SELF-PRUNING | XP_2453931.1 |
| *Sorghum bicolor* | FLOWERING LOCUS T | XP_2438551.1 |

| | | |
|---|---|---|
| *Sorghum bicolor* | FLOWERING LOCUS T | XP_2446272.2 |
| *Sorghum bicolor* | FLOWERING LOCUS T | XP_2451827.2 |
| *Sorghum bicolor* | HEADING DATE 3A | XP_2456354.1 |
| *Sorghum bicolor* | MOTHER of FT/TFL1 homolog 2 | XP_2457494.1 |
| *Spinacia oleraceae* | FT, partial | Data Dryad |
| *Triticum aestivum* | putative kinase inhibitor | BAG12867.1 |
| *Triticum aestivum* | flowering locus T | AAW23034.1 |
| *Triticum aestivum* | flowering locus T | ACA25439.1 |
| *Triticum aestivum* | flowering locus T, partial | ACA25438.1 |
| *Triticum monococcum* | VRN3 | ABK32206.1 |
| *Triticum turgidum* | VRN3 | ABK32207.1 |
| *Vaccinum darrowii* | FT, partial | Data Dryad |
| *Vicia faba* | TFL1, partial | ABP73380.1 |
| *Vigna unguiculata* | terminal flower 1a | BAJ22384.1 |
| *Vigna unguiculata* | terminal flower 1b | BAJ22383.1 |
| *Vitis mustangensis* | terminal flower 1 | ADF80900.1 |
| *Vitis vinifera* | flowering locus T-like protein | ABF56526.1 |
| *Vitis vinifera* | FT-like protein | ABI99465.1 |
| *Vitis vinifera* | flowering locus T | ABL98120.1 |
| *Vitis vinifera* | TFL1A protein | ABI99466.1 |
| *Vitis vinifera* | TFL1B protein | ABI99467.1 |
| *Vitis vinifera* | TFL1C protein | ABI99468.1 |
| *Vitis vinifera* | MFT-like protein | ABI99469.1 |

**Supplemental Table 5.** List of primers used in this study. Restriction sites in 5′-overhangs are underlined. Annealing temperatures (T) are given only for PCR primers.

| Primer name | Sequence (5´to 3´) | T [°C] | purpose |
|---|---|---|---|
| Q35S-P SmaI fw | AGACCCGGGTCCAATCCCACCAAAAC | 58 | Generation of pLab12.10Q35S-P |
| Q35S-P SOE rev | GCTCTTATACTTGAGCGTGTCCT | | |
| TMV Ω TL SOE fw | AGGACACGCTCAAGTATAAGAGC | 58 | |
| TMV Ω TL XhoI rev | AGACTCGAGATGGATAATTGTAAATGTAATTGTAATG | | |
| CcFT1-like NcoI fw | AGACCATGGCGGCGAGGCGGCAGA | 68 | Cloning of pLab12.10Q35S-P::*CcFT1* |
| CcFT1-like XbaI rev | AGATCTAGACTATTGACGGCGCCTGCCGC | | |
| CcFT2 PspXI fw | AGACTCGAGCA ACAATGGCGGCGAGGCGGCAGA | 68 | Cloning of pLab12.1Q35S-P::*CcFT2* |
| CcFT2-like XbaI rev | AGATCTAGACTATTAACGGCGCCTGCCTCCG | | |
| CAS9 fw | ACGTGACCGAGGGAATGAGG | 62 | Analysis of transgene integration into the genome of *Ntft5*$^{-}$ plants |
| CAS9 rev | TTGCAGGAGATCCAGCGAGG | | |
| NtFT4 ExI CRISPR T3 f | ATTGAAGGTCTGTTGATCTTAGAG | - | *NtFT4* protospacer sequence |
| NtFT4 ExI CRISPR T3 r | AAACCTCTAAGATCAACAGACCTT | - | |
| NtFT4 5'UTRf | GAAAAGTAAAGTATTTAGTGTATAG | 48 | Amplification of *NtFT4* (exon I) for sequencing (Schmidt et al., 2020) |
| NtFT4 Intr1 rev | ATGCGAATCAATTATAAATGG | | |
| NtFT5 5'UTR for2 | CCCTTAGACTTGTAAAAACATGC | 53 | Amplification of *NtFT5* (exon I) for sequencing (Schmidt et al., 2020) |
| NtFT5 Intr2 rev3 | ATTGTAGATAGAGTTCCTAGCG | | |
| CcFT-like NcoI fw | AGACCATGGCGGCGAGGCGGCAGA | 68 | Cloning of BIFC constructs |
| CcFT1-like XbaI rev | AGATCTAGACTATTGACGGCGCCTGCCGC | | |
| CcFT-like NcoI fw | AGACCATGGCGGCGAGGCGGCAGA | 68 | |
| CcFT2-like XbaI rev | AGATCTAGACTATTAACGGCGCCTGCCTCCG | | |
| CcFD-like NcoI fw | AGACCATGGGATCTCAGGGCGGTGGT | 61 | |
| CcFD-like NheI rev | AGAGCTAGCTCAAAAAGGATTCGAGCTTGTTC | | |
| CcFT ATG fw | ATGGCGGCGAGGCGGCAGA | 68 | Identification of *CcFT1* genomic sequence |
| CcFT ExonII rev | CACTTGGACTTGGAGCATCAGGATC | | |
| CcFT ExonII fw | GTGATGGTGGATCCTGATGCTC | 63 | |
| CcFT1 gDNA1 II rev | TTTAAGTGGGCATTGATTGTTGGTGTCA | | |
| CcFT1 gDNA2 fw | TGACACCAACAATCAATGCC | 61 | |
| CcFT1 gDNA2 rev | TCGTCTGGAAGTCCATACCCAG | | |
| CcFT1 gDNA3 fw | TCCAGACGAACAATCGTGTTA | 59 | |
| CcFT1 gDNA3 rev | AAATTAGGAGTGACGTATTCTTAT | | |
| CcFT1 gDNA4 fw | TGGATAATCGATCTTTTAGAGTCA | 59 | |
| CcFT1 gDNA4 rev | TCTGTGATACTGTGATGCA | | |
| CcFT1 gDNA5 fw | AAAAGTTAGGAGAACACCAAAT | 60 | |
| CcFT1 gDNA5 rev | TATACCTTTCCACTTGATGACA | | |
| CcFT1 gDNA6 fw | AGTAGCACTTTTAGTTGAGTCA | 59 | |
| CcFT1 gDNA6 rev | AAGTCAAACTGTGACTATCTT | | |
| CcFT1 g_gap fw | GTGTCCAAGTCATTCTCGGATC | 62 | |
| CcFT1 g_gap rev | CACCTTTGCCGCAAGTAGTTG | | |
| CcFT1 gDNA8 fw | GAAGTTCTCTGAACTCACACAAG | 59 | |
| CcFT1 gDNA8 rev | GATAGAAATTAGTTGATAACATTAGGT | | |
| CcFT1 gDNA9 fw | ACCTAATGTTATCAACTAATTTCTATC | 59 | |
| CcFT1 gDNA9 rev | TTTAGTTTTGAACTCTTAAACCTTAAA | | |
| CcFT1 gDNA10 fw | TTTAAGGTTTAAGAGTTCAAAACTAAA | 61 | |
| CcFT1 gDNA10 rev | AGACCCGGAGCAAATAGAT | | |

| | | | |
|---|---|---|---|
| CcFT1 gDNA11 fw | GGTCTGGACCTATGGTCTG | 61 | |
| CcFT1 gDNA11 rev | AGTTCACATTGACTCTATGAGTT | | |
| CcFT1 gDNA12 fw | AACTCATAGAGTCAATGTGAACT | 59 | |
| CcFT1 gDNA12 rev | CCTCACACTAAGACATTATTAAG | | |
| CcFT ATG fw | ATGGCGGCGAGGCGGCAGA | 68 | Identification of *CcFT2* genomic sequence |
| CcFT ExonII rev | CACTTGGACTTGGAGCATCAGGATC | | |
| CcFT ExonII fw | GTGATGGTGGATCCTGATGCTC | 64 | |
| CcFT2-IntII_rev | GGAGAGGGATTTAAGGAGAGTGTA | | |
| CcFT2 gDNA1 fw | TACCCATCACATCAAAGGAC | 59 | |
| CcFT2 gDNA1 rev | CATAACTCATTTCACACTTGTC | | |
| CcFT2 gDNA2 fw | GGCATAATGAACGAATTCGAC | 59 | |
| CcFT2 gDNA2 rev | GTCTTAGTGGAAGGAATAATGC | | |
| CcFT2 gDNA3 fw | CCCGTTCCGAATTAATCCTA | 58 | |
| CcFT2 gDNA3 rev | ACCTTTGCCGCAAGTAGTTC | | |
| CcFT2 gDNA4 fw | AGTCAAGCGTGCTTGACTTGAAG | 64 | |
| CcFT2 gDNA4 rev | TGCTATGAGAATCCTAGAACTAGCCA | | |
| CcFT2 gDNA5 fw | CACGTGAGAAGGGACATG | 59 | |
| CcFT2 gDNA5 rev | TCTGCTCGTGTACGATTACAG | | |
| CcFT2 gDNA6 fw | CATACAGAGTATATCTCAATCCTAGA | 59 | |
| CcFT2 gDNA6 rev | CCAAAATTAACTCCGGTGCTCG | | |
| CcFT2 g_gap1 fw | CTTTCTGACCAAGTACGCCAC | 62 | |
| CcFT2 g_gap1 rev | CGTTCAGCCACAGACTACCTACAG | | |
| CcFT2 g_gap2 fw | CACCCAGGAAGACACAGTAACC | 62 | |
| CcFT2 g_gap2 rev | GTCAGTTGGGAAAGCATGTGC | | |
| CcFT2 g_gap3 fw | GGATTGCCATAAGTCTGTATGC | 62 | |
| CcFT2 g_gap3 rev | GCTGGAACAGTACAAATACGAACC | | |
| CcFT2 gap2 seq fw | CTGCATCAGAATAAGCCTGTAG | - | |
| CcFT2 gap2 seq rev | GTAGTTTTCGACGAGCACAC | - | |
| M13 fw | GTAAAACGACGGCCAG | - | Sequencing of subcloned PCR products |
| M13 bw | CAGGAAACAGCTATGAC | - | |
| 3´Oligo_d(T) | GCTGTCAACGATACGCTACGTAACGGCATGACAGTGT TTTTTTTTTTTTTTTTTTTTTTTT | - | cDNA synthesis for 3´-RACE |
| GSP1 fw | TGCTATGAAAGCCCGCAACC | 64 | 3′-RACE of *CcFT1* and *CaFT* (1. PCR) |
| 3´Adap | GCTGTCAACGATACGCTACGTAACG | | |
| GSP2 fw | TCGTACTGTTCCAGCAGATG | 60 | 3′-RACE of *CcFT1* and *CaFT* (2. PCR) |
| 3´Adap_ nested | CGCTACGTAACGGCATGACAGTG | | |
| CcFT ATG fw | ATGGCGGCGAGGCGGCAGA | 64 | Amplification of *CcFT1*/*CcFT2*/*CaFT* cDNA |
| 3′-UTR CcFT rev | GCGCACGGTGGATTATTGTTCT | | |
| qPCR CcActin fw | ATGGAAGCTGCTGGAATCCAC | 62 | Expression analysis of *Cuscuta* spp. *Actin* by qRT-PCR (Rezaei et al., 2017) |
| qPCR CcActin rev | TTGCTCATACGGTCAGCGATG | | |
| qPCR CaEF1α fw | TCAGACTGTTGCTGTGGGTG | 62 | Expression analysis of *Cuscuta* spp. *EF1a* by qRT-PCR (Zhang et al., 2014) |
| qPCR CaEF1α rev | CCCTTGTGCGGTTCACTTCT | | |
| qPCR CcFT1 disc fw | GGAGAGGTGCTTGAGCCT | 62 | Expression analysis of *CcFT1* and *CaFT* by qRT-PCR |
| qPCR CcFT1 disc rev | CGGGCTTTCATAGCATACC | | |
| qPCR CcFT2 disc fw | GTGATCGGAGAGGTGCTG | 62 | Expression analysis of *CcFT2* by qRT-PCR |
| qPCR CcFT2 disc rev | CTGTGGGCTTTCATAACATACA | | |
| qPCR CcFD fw | ACCGATGACCATGTCTCCAT | 62 | |

| | | | |
|---|---|---|---|
| qPCR CcFD rev | CTTGCTCTCAAGCTCCTGTG | | Expression analysis of *CcFD*-like and *CaFD* by qRT-PCR |
| qRTNt/Ntom EF-1α for | AAGCTGACTGTGCTGTCCTGA | 67 | Expression analysis of *NtEF1α* by qRT-PCR (Beinecke et al., 2018) |
| qRTNt/Ntom EF-1α rev | GGTGGTAGCATCCATCTTGTTG | | |
| qRTNtFT5/NtomFTy for discr | TCCCAAGTTATTAACCAGCCC | 67 | Expression analysis of *NtFT5* by qRT-PCR (Beinecke et al., 2018) |
| qRTNtFT5/NtomFTy rev discr | CATAGCACACAATTTCCTGGC | | |
| qPCR GmF-Box fw | AGATAGGGAAATGGTGCAGGT | 62 | Expression analysis of *GmF-Box* by qRT-PCR (Libault et al., 2008) |
| qPCR GmF-Box rev | CTAATGGCAATTGCAGCTCTC | | |
| qRT Ubi_HKG1 fw | CCAAGCCGAAGAAGATCAAG | 62 | Expression analysis of *AtUbi* by qRT-PCR |
| qRT Ubi_HKG1 rev | ATGACTCGCCATGAAAGTCC | | |
| qRT PP2_HKG2 fw | ACAGCTACGGTCTGGTGTTTTC | 62 | Expression analysis of *AtPP2* by qRT-PCR |
| qRT PP2_HKG2 rev | TCGTGTCGAATTTTCCCGTGATG | | |

**Supplemental Table 6.** Data for CcFT1 and/or CcFt2 expression, time until flowering (d) and number of leaves at the time of flowering on tobacco and Arabidopsis.

| Plant ID | Relative Expression | Days until flowering | No. of leaves at onset of flowering |
|---|---|---|---|
| **CcFT1 - overexpression on tobacco** | | | |
| 1#1 | 0.2067730661 | 90 | 26 |
| 1#2 | 0.1728595650 | 91 | 27 |
| 1#4 | 0.1802287676 | 89 | 25 |
| 1#5 | 0.3753132132 | 76 | 21 |
| 1#6 | 0.9017726117 | 74 | 22 |
| 1#7 | 0.5033205683 | 90 | 26 |
| 1#8 | 1.3516376759 | 68 | 17 |
| 1#10 | 0.3751998785 | 69 | 25 |
| 1#12 | 0.5654169017 | 67 | 18 |
| 1#13 | 0.2841872852 | 74 | 22 |
| 1#14 | 0.2954170555 | 74 | 24 |
| 1#15 | 1.3288333524 | 74 | 22 |
| 1#16 | 0.5889524353 | 83 | 23 |
| 1#17 | 0.5001842534 | 92 | 23 |
| 1#18 | 0.8493423675 | 71 | 20 |
| 1#19 | 0.4829208173 | 77 | 23 |
| 1#20 | 3.3859477121 | 82 | 17 |
| 6#1 | 0.6343422469 | 129 | 72 |
| 6#2 | 0.4548091970 | 160 | 81 |
| 6#5 | 0.4061261982 | 75 | 24 |
| 6#6 | 1.1224620483 | 81 | 26 |
| 6#7 | 0.3789291416 | 95 | 35 |
| 6#8 | 0.4590320100 | 207 | 38 |
| 6#10 | 0.5396141183 | 74 | 21 |
| 6#11 | 0.2952481654 | 109 | 34 |
| 6#12 | 0.4741230156 | 183 | 33 |
| 6#13 | 1.1566881839 | 74 | 20 |
| 6#14 | 0.9308797161 | 205 | 42 |
| 6#15 | 0.3314048604 | 131 | 75 |
| 6#16 | 0.2206757491 | 102 | 38 |
| 6#17 | 0.8467453124 | 94 | 40 |
| 6#18 | 0.6141515747 | 107 | 53 |
| 9#2 | 0.6282041082 | 74 | 22 |
| 9#3 | 0.4554078601 | 70 | 21 |
| 9#5 | 0.3913214324 | 74 | 21 |
| 9#6 | 1.4419544036 | 71 | 21 |
| 9#7 | 0.5131743556 | 71 | 20 |
| 9#8 | 0.5243743157 | 70 | 21 |
| 9#10 | 0.6221000786 | 88 | 24 |
| 9#11 | 0.6302383947 | 73 | 22 |
| 9#12 | 0.3106841572 | 75 | 21 |
| 9#13 | 1.0686859862 | 90 | 21 |
| 9#14 | 0.4932683898 | 88 | 22 |

| | | | |
|---|---|---|---|
| 9#15 | 0.8379214912 | 90 | 23 |
| 9#17 | 0.8774354893 | 70 | 22 |
| 9#18 | 0.1339198935 | 74 | 22 |
| 9#20* | 0.0192464231 | >183 | >70 |
| 6#4* | 0.2612192882 | >183 | >70 |
| ***CcFT2* - overexpression in tobacco** | | | |
| 2#1 | 2.037591234 | 102 | 26 |
| 2#2 | 2.823738176 | 83 | 28 |
| 2#3 | 2.095012615 | 90 | 30 |
| 2#5 | 3.019717501 | 74 | 30 |
| 2#7 | 1.247004635 | 76 | 26 |
| 2#8 | 1.642040414 | 77 | 26 |
| 2#9 | 1.656559291 | 83 | 28 |
| 2#10 | 4.011194804 | 85 | 33 |
| 2#11 | 4.641326827 | 73 | 24 |
| 2#12 | 4.037542538 | 76 | 25 |
| 2#16 | 2.185010505 | 91 | 31 |
| 2#18 | 1.872439854 | 77 | 28 |
| 4#1 | 14.6249495 | 74 | 20 |
| 4#2 | 7.428662026 | 73 | 21 |
| 4#3 | 13.40485268 | 73 | 19 |
| 4#4 | 4.186376451 | 74 | 23 |
| 4#5 | 5.424698488 | 74 | 25 |
| 4#6 | 7.827817545 | 75 | 22 |
| 4#7 | 2.105576421 | 81 | 28 |
| 4#8 | 9.131467961 | 74 | 21 |
| 4#9 | 4.802371353 | 74 | 25 |
| 4#11 | 5.869765608 | 74 | 23 |
| 4#13 | 2.509631926 | 77 | 26 |
| 4#14 | 5.264760997 | 74 | 23 |
| 4#15 | 2.794895058 | 83 | 26 |
| 4#16 | 7.353500122 | 76 | 23 |
| 4#17 | 8.351269009 | 73 | 23 |
| 4#18 | 3.785101609 | 76 | 21 |
| 4#20 | 4.805185577 | 76 | 27 |
| 8#1 | 2.133443179 | 89 | 30 |
| 8#3 | 3.030158757 | 71 | 23 |
| 8#4 | 1.581568999 | 76 | 27 |
| 8#5 | 2.345474659 | 74 | 22 |
| 8#6 | 1.807947341 | 74 | 23 |
| 8#7 | 5.182002715 | 70 | 22 |
| 8#8 | 4.280542984 | 70 | 22 |
| 8#9 | 3.213035066 | 73 | 25 |
| 8#10 | 2.238721346 | 81 | 26 |
| 8#11 | 0.795006423 | 130 | 40 |
| 8#12 | 2.103198431 | 81 | 22 |
| 8#13 | 4.780948872 | 71 | 22 |

| | | | | |
|---|---|---|---|---|
| 8#14 | 7.036158485 | 70 | 21 | |
| 8#16 | 1.813303493 | 112 | 32 | |
| 8#17 | 2.950274677 | 73 | 23 | |
| 8#18 | 2.579505831 | 74 | 21 | |
| 8#19 | 6.165470159 | 70 | 21 | |
| 8#20 | 6.546006148 | 70 | 22 | |
| **CcFt overexpression on *Arabidopsis*** | | | | |
| **Sample** | **Relative CcFT1 expression** | **Days until flowering** | **Rosette leaves** | **Cauline leaves** |
| L01 #01 | 0.14396 | 88 | 51 | 10 |
| L01 #08 | 0.32303 | 80 | 44 | 7 |
| L01 #09 | 0.00019 | 90 | 58 | 13 |
| L01 #10 | 7.62724 | 86 | 51 | 11 |
| L01 #11 | 0.31998 | 84 | 46 | 9 |
| L01 #13 | 0.26894 | 79 | 45 | 9 |
| L01 #17 | 0.27342 | 77 | 48 | 9 |
| L01 #21 | 0.00063 | 85 | 54 | 11 |
| L01 #22 | 0.00054 | 91 | 55 | 11 |
| L03 #01 | 0.00121 | 85 | 38 | 7 |
| L03 #11 | 0.25602 | 76 | 34 | 6 |
| L03 #12 | 33.98078 | 84 | 37 | 6 |
| L03 #15 | 6.20036 | 83 | 41 | 7 |
| L03 #17 | 6.7494 | 76 | 37 | 5 |
| L03 #18 | 16.01514 | 97 | 41 | 7 |
| L03 #20 | 0.11643 | 75 | 37 | 6 |
| L03 #22 | 3.53241 | 94 | 38 | 5 |
| L12 #08 | 51.22309 | 77 | 37 | 6 |
| L12 #17 | 18.70006 | 77 | 38 | 6 |
| L12 #18 | 0.68332 | 77 | 39 | 7 |
| L12 #26 | 0.00111 | 91 | 48 | 6 |
| L12 #31 | 0.05698 | 68 | 33 | 7 |
| L12 #34 | 34.42638 | 68 | 38 | 6 |
| L12 #38 | 0.00025 | 87 | 58 | 10 |
| L12 #42 | 13.10201 | 68 | 34 | 6 |

* non-flowering individuals

## Supplemental Datasets

**Dataset S1 (*separate file*). Results of exonerate-based genic conservation analysis of 295 flowering time regulating genes in *Cuscuta* spp. and 13 related eudicots.** Gene and reading frame conservation is provided as length identity score from exonerate-based *protein2genome* alignments, using validated reference proteins of *Arabidopsis thaliana* as queries. In addition to genomic data searches of *Cuscuta* spp., we also examined transcriptome assemblies, the results of which given in separate columns. Finally, we computed the divergence of *Cuscuta* flowering gene conservation relative to that of the mean length identity score of the 13 nonparasitic eudicots, whereby values >1 indicate stronger conservation and/or longer reading frames in *Cuscuta* spp. than the average, and values <1 suggest less conservation and/or shorter genes in the parasites. Datasets with recovered genic fragments are published as reusable fasta files in the Dryad Data Repository, https://datadryad.org/stash/share/DK8OIh2VqFwbGNL0GtkGt24dD0GhWhJn82oLBC1XK70.

**Dataset S2 (*separate file*). Domain-based annotation of transposable elements (DANTE) and inferred fragments.** Filtered and unfiltered database hits of DANTE searches within the extended *CaFT*, *CcFT1* and *CcFT2* gene regions are summarized by length, identity, similarity, and number of interruptions. The retrieved and reference transposable element domains are provided for every hit, indicating interruptions such as gaps and stop codons by backslashes and asterisks, respectively. Datasets with recovered genic fragments are published as reusable fasta files in the Dryad Data Repository, https://datadryad.org/stash/share/DK8OIh2VqFwbGNL0GtkGt24dD0GhWhJn82oLBC1XK70.
