# Supplementary material for: Gene complementation analysis indicates that parasitic dodder plants do not depend on the host FT protein for flowering
Source: Plant Commun. 2024 Jan 29;5(5):100826. doi: 10.1016/j.xplc.2024.100826 (PMC11121739; doi:10.1016/j.xplc.2024.100826)
Supplement: Document S2. Article plus supplemental information [file mmc4.pdf]

Plant Communications
Correspondence

CellPress Partner Journal

Gene complementation analysis indicates that parasitic dodder plants do not depend on the host FT protein for flowering

Dear Editor,

Cuscuta, a genus of parasitic plants, poses a significant threat to global agriculture due to its broad host range that encompasses numerous cultivated crops. The plants form specialized feeding organs (haustoria) that connect to the host vascular tissue and import water, nutrients, secondary metabolites, small RNAs, mRNAs, and proteins. One such protein is FT, a peptide hormone and florigen that was recently proposed to cause the synchronization of flowering in the parasite–host system (Shen et al., 2020). Flowering is tightly controlled by endogenous and environmental cues, ensuring that floral development occurs only under favorable conditions. FT is a major network hub that integrates signals from several floral pathways and is the mobile signal that moves from leaves to the shoot apical meristem, where it interacts with its cofactor FD and 14-3-3 proteins to promote flowering (Taoka et al., 2011; Blümel et al., 2015). Genome analysis in *C. australis* and *C. campestris* indicated the loss of multiple floral regulator genes (Sun et al., 2018; Vogel et al., 2018), suggesting that the translocated host FT protein may complement the incomplete regulatory network in the parasite by forming a complex with endogenous FD to enable floral transition (Shen et al., 2020). However, *Cuscuta* species flower independently *in vitro* (Bernal Galeano et al., 2022). Furthermore, host flowering status has never affected the flowering time of *C. campestris* in our laboratories.

To investigate this in more detail, we used tobacco (*Nicotiana tabacum*) as the host plant, which, like *Cuscuta*, belongs to the order of Solanales. Tobacco is a day-neutral plant that produces two FT floral inducers (NtFT4 and NtFT5) and two FT floral repressors (NtFT1 and NtFT2). NtFT5 is predominantly responsible for floral induction under long-day (LD) conditions, whereas both NtFT4 and NtFT5 induce flowering under short-day (SD) conditions. NtFT1 is responsible for floral repression under LD conditions, whereas both NtFT1 and NtFT2 repress flowering under SD conditions (Harig et al., 2012; Beinecke et al., 2018). First, we generated non-flowering tobacco (*N. tabacum*) host plants by mutating the two tobacco floral activators NtFT4 and NtFT5 (Schmidt et al., 2020; Supplemental Figure 1). We allowed *C. campestris* to parasitize wild-type tobacco and two independent *Ntft4⁻Ntft5⁻* T_2 lines. The wild-type plants flowered after 66 days, whereas onset of flowering had not occurred in *Ntft4⁻Ntft5⁻* plants by the end of the experiment (83 days). *C. campestris* plants parasitizing wild-type tobacco flowered after 42 days, and those parasitizing the two double-mutant hosts flowered after 39 and 42 days, respectively (Figure 1A). Conversely, we tested whether FT overexpression in the host influences *C. campestris* flowering time. As NtFT5 overexpression in the wild-type tobacco background causes extremely early flowering, making infestation with *Cuscuta* impossible, we used tobacco hosts with a knockout mutation in NtFT5 and simultaneous overexpression of NtFT5 using the constitutive 35S promoter (35S:*NtFT5*$_{L4}$//SR1Δ*NtFT5*), whose flowering time is shortened by approximately 20% compared with that of the controls (Zimmermann et al., 2022). We found that flowering time of *Cuscuta* did not change significantly when feeding on the 35S:*NtFT5*$_{L4}$//SR1Δ*NtFT5* plants compared with control hosts (Supplemental Figure 2). These data suggest that *C. campestris* does not rely on the host's FT signal.

To examine the endogenous FT–FD module in *C. campestris*, we compared 295 protein-coding genes associated with flowering time in multiple species, revealing only two putative gene losses specific to *Cuscuta* (*GIBBERELLIN 2-OXIDASE 8* and *UBIQUITIN-SPECIFIC PROTEASE 26*). Other losses and major sequence deviations compared with *Arabidopsis* were shared with other eudicots (Figure 1B; Supplemental Data 1). We sequenced the complete *CcFT1* and *CcFT2* genes and coding sequences, revealing a non-intact *Ty1/copia Ale*-type retrotransposon in the third intron of *CcFT2* and a LINE-type reverse transcriptase upstream of *CcFT1* and the *C. australis* ortholog *CaFT* (Supplemental Data 2), but RT–PCR indicated that all *FT* mRNAs were correctly spliced (Supplemental Figure 3). The deduced amino acid sequences of CcFT1 and CaFT were identical. A search for FD homologs revealed seven new *FD*-like sequences in *C. australis* and five in *C. campestris*. The association of FDs with 14-3-3 proteins and their interaction with FTs depends on the phosphorylation of serine/threonine residues in the SAP motif (Taoka et al., 2011). Only two of the *C. campestris FD*-like transcripts contained a SAP-like motif (*CcFD*-like1 and *CcFD*-like2), and their amino acid sequences were 99% identical. We also found a sequence identical to *CcFD-like1* in *C. australis* (*CaFD-like*), which differed from the published *CaFD* sequence that lacks a SAP motif (Supplemental Figure 4) and was reported not to interact with CaFT. We analyzed the expression of the *CcFD*-like and *CcFT* genes and detected strong *CcFD*-like expression in haustoria, buds, and flowers and slightly weaker expression in stem tips, stems, leaves, and branches. We detected *CcFT1* and *CcFT2* expression only in haustoria (low levels in both cases). Similarly, *CaFD*-like genes were expressed in all tissues, whereas *CaFT* was haustorium specific (Figure 1C). These results were confirmed by RNA sequencing analysis of FT, FD,

Published by the Plant Communications Shanghai Editorial Office in association with Cell Press, an imprint of Elsevier Inc., on behalf of CSPB and CEMPS, CAS.

This is an open access article under the CC BY license (http://creativecommons.org/licenses/by/4.0/).

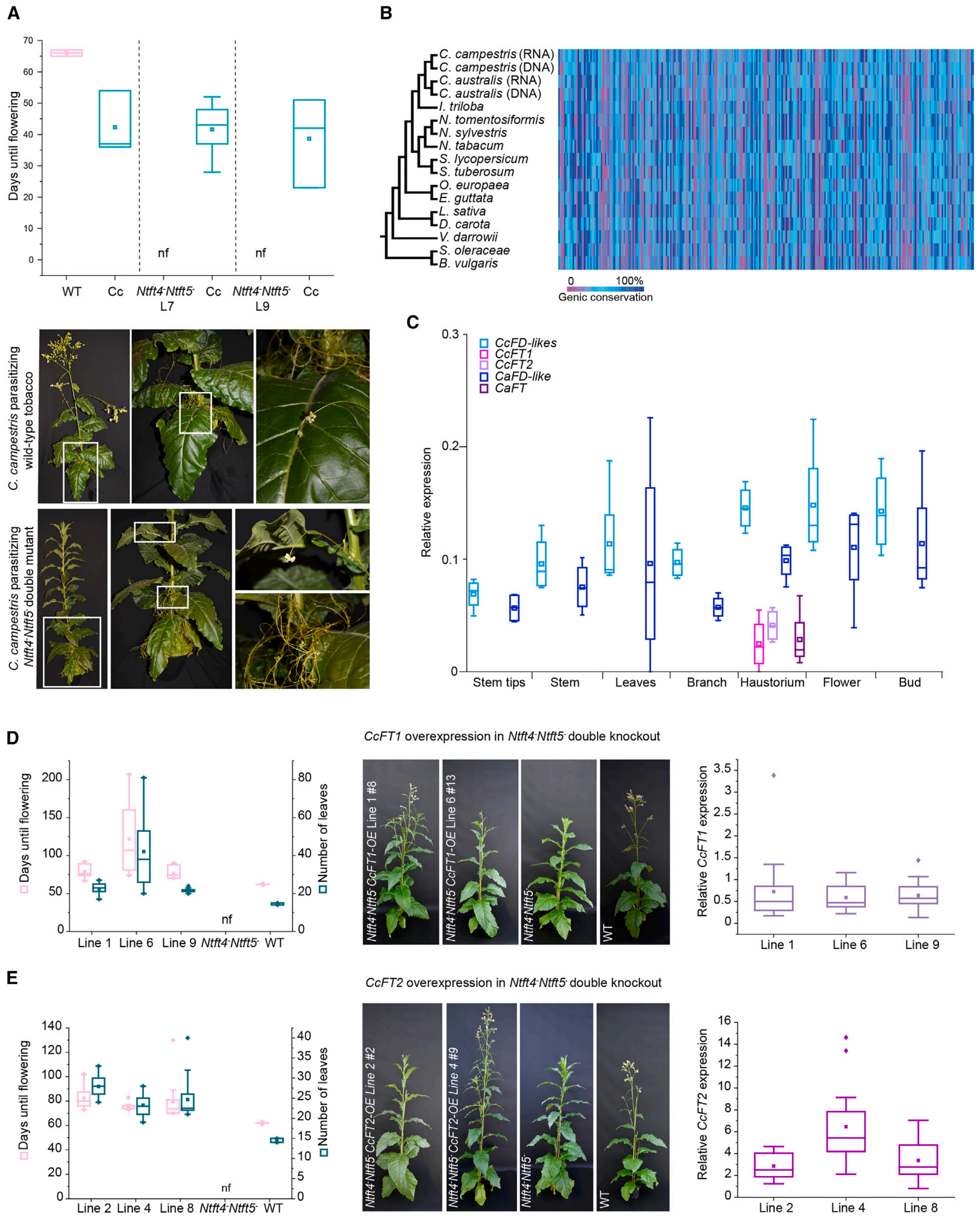

Figure 1. Analysis of flowering, conservation of flowering genes (heatmap), expression analysis, and overexpression experiments in *Cuscuta* plants to investigate the role of host FT.

(A) *Cuscuta campestris* was allowed to parasitize tobacco SR1 wild-type (WT) plants or two independent *Ntft4*$^-$*Ntft5*$^-$ double-knockout mutants (lines 7 and 9). The presence or absence of floral activating FTs in the host did not influence *C. campestris* flowering time, as determined by analysis of variance

(legend continued on next page)

and other floral regulators, which we detected in both species (Supplemental Tables 1 and 2). Together, these results show that *C. campestris* expresses two paralogs each of FT and its cofactor FD, as well as other flowering time regulators that interact with them. In contrast to previous findings (Shen et al., 2020), we also detected *CaFT* and *CaFD*-like gene expression in *C. australis*, probably because we analyzed *CaFT* and *CaFD*-like expression in different plant organs, whereas Shen et al. (2020) focused on different developmental stages of stem tissues. In addition, bimolecular fluorescence complementation revealed that both CcFT1 and CcFT2 interacted with CcFD-like1 in the nucleus of *Nicotiana benthamiana* epidermal cells in transient expression experiments. CcFT1 and CcFT2 also interacted with NtFD1, and CcFD-like1 interacted with NtFT5 (Supplemental Figure 5). These results highlight the conservation of the FT–FD module across species. Taken together, these results indicate that an endogenous FT–FD protein complex can form in both *Cuscuta* species.

To test whether CcFT1 is a functional floral activator, we overexpressed its coding sequence in the wild-type tobacco background. We found that CcFT1 overexpression failed to induce early flowering (Supplemental Figure 6), indicating that CcFT1 is unable to surpass the function of the two endogenous floral activators, NtFT4 and NtFT5. To avoid the endogenous expression of activating FTs, we determined the ability of CcFT1 and CcFT2 to complement the non-flowering phenotype of *Ntft4⁻Ntft5⁻* plants. Under LD conditions, *Ntft4⁻Ntft5⁻* plants overexpressing *CcFT1* flowered 67–207 days after sowing (Figure 1D). Only two of 48 plants remained vegetative after 233 days, probably owing to low *CcFT1* expression levels (Supplemental Figure 7). *Ntft4⁻Ntft5⁻* plants overexpressing *CcFT2* flowered 70–130 days after sowing, whereas the *Ntft4⁻Ntft5⁻* control plants had not flowered after 233 days (Figure 1E). Higher *CcFT1* and *CcFT2* expression in the complemented *Ntft4⁻Ntft5⁻* plants were both significantly correlated with earlier flowering (Spearman rank correlation, one-tailed, *CcFT1*: rs = -0.2885, $p = 0.023$, $n = 48$; *CcFT2*: r_s = -0.6546, $p < 0.001$, $n = 47$) and fewer leaves until flowering (*CcFT1*: $r_s = -0.3425$, $p = 0.009$, $n = 48$; *CcFT2*: $r_s = -0.6658$, $p < 0.001$, $n = 47$; Supplemental Table S6). In contrast to Shen et al. (2020), we used an enhanced version of the CaMV35S promoter in conjunction with the TMV Ω leader to achieve optimal translation initiation efficiency for all our CcFT overexpression experiments, which revealed that CcFT1 also induces early flowering in *Arabidopsis thaliana* Col-0 wild-type plants under SD conditions (Supplemental Figure 8). We observed that increased FT gene expression coincided with early flowering (Spearman rank correlation, one-tailed: rs = -0.3404, $p = 0.048$, $n = 25$), expedited rosette development ($r_s = -0.5187$, $p = 0.004$, $n = 25$) and cauline leaf formation ($r_s = -0.5579$, $p = 0.002$, $n = 25$). These results show that CcFT1/CaFT and CcFT2 can induce floral transition in a heterologous system and should be considered functional floral activators.

Our data show that *C. campestris* does not synchronize its flowering time with the host and can induce flowering without a host FT signal. Furthermore, *C. campestris* and *C. australis* express endogenous *FT* and *FD-like* orthologs. *Cuscuta* species also produce sensors of both short-wave and long-wave light (Supplemental Data 1) that act upstream of FT. This aligns with *in vitro* cultivation protocols, which use photoperiod shifts to induce flowering in host-free *Cuscuta* plants (Baldev, 1962). An earlier claim that the *Cuscuta* flowering time network is impaired was based on the inferred loss of genes with key roles in *Arabidopsis*, such as CO and FLC (Sun et al., 2018; Shen et al.,

and Tukey's post hoc test (n ≥ 3 biological replicates; nf, non-flowering until the end of experiment, 83 days after sowing). Photos were taken 83 days after sowing (magnified areas are indicated by white squares).

(B) Heatmap showing the conservation of flowering-time genes (and corresponding coding regions) in the genomes of *Cuscuta* spp. and 13 closely related eudicots. Small blocks in columns represent each of the 295 protein-coding genes associated with flowering time (detailed in Supplemental Data 1). Regarding gene conservation, blue indicates a conserved length and amino acid sequence, cyan shows genic drift, and magenta indicates the absence of a coding region. Each line corresponds to one of the analyzed 13 eudicots and *Cuscuta* spp., whose vertical order is displayed in a phylogenetic context as illustrated on the left-hand side. For the two *Cuscuta* species, *C. australis* and *C. campestris*, gene conservation was analyzed at both the genome data level and the RNA (transcriptome) level, as indicated by "DNA" or "RNA," respectively. Further details of the analysis are available in the supplemental materials and methods and Supplemental Data 1.

(C) Expression analysis of *FD* and *FT* homologs in different *Cuscuta* tissues. *C. campestris* tissue was harvested from flowering plants parasitizing WT tobacco. *C. australis* tissue was harvested from flowering plants parasitizing soybean. *CcFD-like1* and *CcFD-like2* expression levels were determined using non-discriminating primers. Relative expression levels of *CcFT1*, *CcFT2*, *CcFD-like* genes, *CaFT1*, and *CaFD-like* were determined by RT–qPCR using *Actin* and *EF1α* as reference genes ($n = 4$ biological replicates).

(D) Overexpression of *CcFT1* in *Ntft4⁻Ntft5⁻* tobacco plants. Flowering time and number of leaves at flowering of *Ntft4⁻Ntft5⁻* plants overexpressing *CcFT1* compared with *Ntft4⁻Ntft5⁻* and WT controls. Days until flowering and number of leaves were determined when the first bud had fully opened. Data represent $n = 17$ (line 1), 15 (line 6), 14 (line 9), 12 (*Ntft4⁻Ntft5⁻*), and 6 (WT) plants. Statistics for the individual treatment groups were not assessed, as no data points were available for non-flowering plants. Phenotypes of *Ntft4⁻Ntft5⁻* plants with (exemplarily shown for line 1 and line 6) or without (*Ntft4⁻Ntft5⁻*) heterologous expression of *CcFT1* compared with WT controls were photographed when WT plants flowered. Relative expression levels of *CcFT1* in *Ntft4⁻Ntft5⁻* plants overexpressing *CcFT1* were determined by RT–qPCR using *NtEF1α* as the reference gene (nf, non-flowering until end of experiment, 233 days after sowing).

(E) Overexpression of *CcFT2* in *Ntft4⁻Ntft5⁻* double mutants of tobacco. Flowering time and number of leaves at flowering of *Ntft4⁻Ntft5⁻* plants overexpressing *CcFT2* compared with *Ntft4⁻Ntft5⁻* and WT controls. Days until flowering and number of leaves were determined when the first bud had fully opened. Data represent $n = 12$ (line 2), 17 (line 4), 18 (line 8), 13 (*Ntft4⁻Ntft5⁻*), and 6 (WT) plants. Statistics for the individual treatment groups were not assessed, as no data points were available for non-flowering plants. Phenotypes of *Ntft4⁻Ntft5⁻* plants with (exemplarily shown for line 2 and line 4) or without (*Ntft4⁻Ntft5⁻*) the heterologous expression of *CcFT2* compared with WT controls were photographed when WT plants flowered. Relative expression levels of *CcFT2* in *Ntft4⁻Ntft5⁻* plants overexpressing *CcFT2* were determined by RT–qPCR using *NtEF1α* as the reference gene (nf, non-flowering until end of experiment, 233 days after sowing). In the boxplots, center line = median, square = mean, box limits = upper and lower quartiles, whiskers = 1.5× interquartile range, and diamonds = outliers.

2020). Although CO is present in several angiosperm lineages (e.g., monocots), its role as a positive regulator of photoperiod-dependent floral induction is limited outside the Brassicaceae. The CO pathway evolved from CO-like genes following gene duplication within this family (Simon et al., 2015). Moreover, the vernalization response, mainly controlled by FLC in *Arabidopsis*, has convergently evolved after the continental drift. For example, it has been shown that FLC homologs are not involved in the vernalization response pathways of cereals and Amaranthaceae (supplemental information). Floral transition genes have been studied in many species other than *Arabidopsis*, revealing numerous alternative genetic networks that regulate flowering time (Blümel et al., 2015). In potato (*Solanum tuberosum*), a close relative of tobacco, activating and repressing FT homologs not only control sexual reproduction but also vegetative reproduction by coordinating the floral transition and tuberization (supplemental information). Sugar beet (*Beta vulgaris*; Chenopodiaceae) has no functional CO ortholog, and its FLC homolog does not play a major role in the vernalization response (supplemental information). Instead, sugar beet produces a pair of antagonistic FT homologs that are differentially regulated in annuals and biennials by the joint action of BTC1 and BvBBX19, which together may integrate temperature and photoperiod signals (supplemental information). These are clear examples in which floral regulating pathways diverge from the *Arabidopsis* model, showcasing adaptations that have evolved during the diversification of angiosperms and their colonization of different environmental niches (Blümel et al., 2015).

The expression of *Cuscuta* FT homologs was detected only in the haustoria, not in other tissues. In parasitic plants, perception of the host's physiological status is likely to facilitate successful reproduction by ensuring a sufficient energy supply for growth, floral induction, seed maturation, and ripening. This agrees with the fact that *FT* expression depends not only on endogenous cues such as hormones and plant age and seasonal cues such as temperature or photoperiod but also on carbon assimilation rates and sugar levels (supplemental information). Indeed, excised *C. campestris* stem tips only require sugars for growth and flowering (supplemental information). It remains unclear whether and to what extent the host contributes to floral induction in *Cuscuta*. A combination of direct experimental and circumstantial evidence suggests that the host provides physiological cues to induce flowering in the parasite but that the genetic components of the parasite's floral induction pathway are endogenous. We have demonstrated that *C. campestris* and *C. australis* possess an endogenous FT–FD module, providing a reference point for identification of the upstream regulators and downstream targets.

DATA AND CODE AVAILABILITY

All sequence data reported herein, including target-assembled protein-coding genes, alignment files, and gene family trees, as well as unscaled figures, have been published in standardized, reusable .fasta, .nexus, and .newick file formats at the Dryad Data Repository (https://datadryad.org/stash/share/DK8Olh2VqFwbGNL0GtkGt24dD0GhWhJn82oLBC1XK70). All newly generated sequence data have been deposited at NCBI GenBank under accession numbers NCBI: OP995431–OP995444.

SUPPLEMENTAL INFORMATION

Supplemental information is available at *Plant Communications Online*.

FUNDING

This study was supported by the German Science Foundation (DFG, WI4507/3-1 to S.W.) and the Fraunhofer internal funding program PREPARE to G.A.N. and D.P.

AUTHOR CONTRIBUTIONS

Conceptualization, S.M., A.K., G.A.N., and S.W.; investigation, S.M., A.K., L.M.K., and P.L.; validation, S.M. and A.K.; visualization, S.M., A.K., and S.W.; writing – original draft, A.K., D.P., G.A.N., and S.W.; writing – review & editing, D.P., G.A.N., and S.W.; data analysis, L.M.K., P.L., and S.W.; funding acquisition, D.P. and S.W.; supervision, D.P.; project administration, G.A.N. and S.W.

ACKNOWLEDGMENTS

The authors would like to thank Dr. Kirsten Krause (UIT, The Arctic University of Norway) and Dr. Jianqiang Wu (Kunming Institute of Botany, China) for providing plant material and seeds of *C. campestris* and *C. australis*, respectively. We thank Alina Griese, Michael Lahme, Andreas Wagner, and Dr. Jost Muth for technical and horticultural assistance. We thank Richard M. Twyman for critically reading the manuscript. No conflict of interest is declared.

Received: February 21, 2023
Revised: January 11, 2024
Accepted: January 24, 2024
Published: January 29, 2024

Sina Mäckelmann[1,4], Andrea Känel[1,4], Lara M. Kösters[2], Peter Lyko[2], Dirk Prüfer[1,3], Gundula A. Noll[1,3,] and Susann Wicke[2,*]*

[1]Institute of Plant Biology and Biotechnology, University of Muenster, Schlossplatz 8, 48143 Muenster, Germany
[2]Institute for Biology, Humboldt-University of Berlin, Haus 22, Philippstr. 13, 10115 Berlin, Germany
[3]Fraunhofer Institute for Molecular Biology and Applied Ecology IME, Schlossplatz 8, 48143 Muenster, Germany
[4]These authors contributed equally to this article.
***Correspondence: Gundula A. Noll (gnoll@uni-muenster.de), Susann Wicke (susann.wicke@hu-berlin.de)**
https://doi.org/10.1016/j.xplc.2024.100826

REFERENCES

Baldev, B. (1962). *In vitro* studies of floral induction on stem apices of *Cuscuta reflexa* Roxb.—a short-day plant. Ann. Bot. **26**:173–174. https://doi.org/10.1093/oxfordjournals.aob.a083784.

Beinecke, F.A., Grundmann, L., Wiedmann, D.R., Schmidt, F.J., Caesar, A.S., Zimmermann, M., Lahme, M., Twyman, R.M., Prüfer, D., and Noll, G.A. (2018). The FT/FD-dependent initiation of flowering under long-day conditions in the day-neutral species Nicotiana tabacum originates from the facultative short-day ancestor Nicotiana tomentosiformis. Plant J. **96**:329–342. https://doi.org/10.1111/tpj.14033.

Bernal-Galeano, V., Beard, K., and Westwood, J.H. (2022). An artificial host system enables the obligate parasite *Cuscuta campestris* to grow and reproduce in vitro. Plant Physiol. **189**:687–702. https://doi.org/10.1093/plphys/kiac106.

Blümel, M., Dally, N., and Jung, C. (2015). Flowering time regulation in crops—what did we learn from *Arabidopsis*? Curr. Opin. Biotechnol. **32**:121–129. https://doi.org/10.1016/j.copbio.2014.11.023.

Harig, L., Beinecke, F.A., Oltmanns, J., Muth, J., Müller, O., Rüping, B., Twyman, R.M., Fischer, R., Prüfer, D., and Noll, G.A. (2012). Proteins

from the FLOWERING LOCUS T-like subclade of the PEBP family act antagonistically to regulate floral initiation in tobacco. Plant J. **72**:908–921. https://doi.org/10.1111/j.1365-313X.2012.05125.x.

Schmidt, F.J., Zimmermann, M.M., Wiedmann, D.R., Lichtenauer, S., Grundmann, L., Muth, J., Twyman, R.M., Prüfer, D., and Noll, G.A. (2020). The major floral promoter NtFT5 in tobacco (*Nicotiana tabacum*) is a promising target for crop improvement. Front. Plant Sci. **10**, 1666. https://doi.org/10.3389/fpls.2019.01666.

Shen, G., Liu, N., Zhang, J., Xu, Y., Baldwin, I.T., and Wu, J. (2020). *Cuscuta australis* (dodder) parasite eavesdrops on the host plants' FT signals to flower. Proc. Natl. Acad. Sci. USA **117**:23125–23130. https://doi.org/10.1073/pnas.2009445117.

Simon, S., Rühl, M., de Montaigu, A., Wötzel, S., and Coupland, G. (2015). Evolution of CONSTANS regulation and function after gene duplication produced a photoperiodic flowering switch in the Brassicaceae. Mol. Biol. Evol. **32**:2284–2301. https://doi.org/10.1093/molbev/msv110.

Sun, G., Xu, Y., Liu, H., Sun, T., Zhang, J., Hettenhausen, C., Shen, G., Qi, J., Qin, Y., Li, J., et al. (2018). Large-scale gene losses underlie the genome evolution of parasitic plant Cuscuta australis. Nat. Commun. **9**:2683. https://doi.org/10.1038/s41467-018-04721-8.

Taoka, K.I., Ohki, I., Tsuji, H., Furuita, K., Hayashi, K., Yanase, T., Yamaguchi, M., Nakashima, C., Purwestri, Y.A., Tamaki, S., et al. (2011). 14-3-3 proteins act as intracellular receptors for rice Hd3a florigen. Nature **476**:332–335. https://doi.org/10.1038/nature10272.

Vogel, A., Schwacke, R., Denton, A.K., Usadel, B., Hollmann, J., Fischer, K., Bolger, A., Schmidt, M.H.-W., Bolger, M.E., Gundlach, H., et al. (2018). Footprints of parasitism in the genome of the parasitic flowering plant *Cuscuta campestris*. Nat. Commun. **9**:2515. https://doi.org/10.1038/s41467-018-04344-z.

Zimmermann, M.M., Grundmann, L., Känel, A., Schwarze, A., Wiedmann, D.R., Muth, J., Twyman, R.M., Prüfer, D., and Noll, G.A. (2022). Unraveling the mystery behind the short-day-specific flowering of tobacco cultivar Maryland Mammoth. Preprint at bioRxiv. https://doi.org/10.1101/2022.06.27.497884.

Plant Communications, Volume *5*

Supplemental information

Gene complementation analysis indicates that parasitic dodder plants do not depend on the host FT protein for flowering

Sina Mäckelmann, Andrea Känel, Lara M. Kösters, Peter Lyko, Dirk Prüfer, Gundula A. Noll, and Susann Wicke

Supplemental Information

Gene complementation analysis indicates that parasitic dodder plants do not depend on the host FT protein for flowering

Sina Mäckelmann[1¶], Andrea Känel[1¶], Lara M. Kösters[2], Peter Lyko[2], Dirk Prüfer[1,3], Gundula A. Noll[1,3*], and Susann Wicke[2*]

[1] Institute of Plant Biology and Biotechnology, University of Muenster, Schlossplatz 8, 48143, Muenster, Germany

[2] Institute of Biology, Humboldt-Universität zu Berlin, Haus 22, Philippstr. 13, 10115, Berlin, Germany

[3] Fraunhofer Institute for Molecular Biology and Applied Ecology IME, Schlossplatz 8, 48143, Muenster, Germany

[¶] These authors contributed equally to this work

* Corresponding authors: Gundula A. Noll, Susann Wicke

Email: gnoll@uni-muenster.de; susann.wicke@hu-berlin.de

Supplemental materials and methods

Additional information on study background and experimental design. Due to constraints on word count and citation numbers, it was not feasible to incorporate all pertinent background information into the main text. Nevertheless, we wish to acknowledge the following studies for their crucial contributions: Ream et al. (2012) conducted a comprehensive review of the FLC-independent vernalization response in Amaranthaceae and cereals. Navarro et al. (2015) delved into the functional analysis of antagonistically acting FTs that govern sexual and vegetative reproduction in potatoes. Chia et al. (2008) and Vogt et al. (2014) investigated the roles of CO and FLC homologues in *Beta vulgaris*, respectively. Pin et al. (2010, 2012) and Dally et al. (2018) explored antagonistic FT proteins from *Beta vulgaris* and their regulators BTC1 and BvBBX19. Additionally, Cho et al. (2018) provided insights into the role of sugars in flowering time control. Notably, Loo (1946) demonstrated the sole requirement of sugars for growth and flowering in tissue culture of *Cuscuta campestris* stem tips.

Plant material and cultivation conditions. This study used tobacco (*Nicotiana tabacum* L. cv. Petit Havana SR1), *Ntft5*⁻ mutant plants originating from the nullizygous T_2 progeny of line #78 without the pDECAS9-*NtFT5*$_{\text{ex I-147.169bp}}$ transgene (Schmidt et al., 2020), *Ntft5*⁻ mutant plants which overexpress NtFT5 under control of the 35S promoter (35S:*NtFT5*$_{\text{L4}}$//SR1Δ*NtFT5*; Zimmermann et al., 2022), *Arabidopsis thaliana* Col-0 and soybean (*Glycine max* cv. Summer Shell). If not stated otherwise, plants were cultivated under long-day (LD) conditions in a climate-controlled greenhouse (16-h photoperiod, artificial light switched on if natural light fell below 700 µmol m^{-2} s^{-1}, 22–25 °C under light, 19–25 °C in the dark). Alternatively, plants were cultivated in phytochambers under LD conditions (16-h photoperiod, 200 µmol m^{-2} s^{-1}, 25–27 °C (tobacco) or 22°C (*Arabidopsis*) under light, 20 °C in the dark) or under short-day (SD) conditions in phytochambers (8-h photoperiod, 200 µmol m^{-2} s^{-1}, 25–27 °C under light, 20 °C in the dark). *Cuscuta* spp. originated from the previous study material of the UIT Arctic University of Norway and the Kunming Institute of Botany (Vogel et al., 2018; Sun et al., 2018) and were harvested from the living research collections at the University of Münster and the Späth Arboretum, Humboldt-Universität zu Berlin. Before germination and infection of host plants, *Cuscuta* spp. seeds were immersed in 97% sulfuric acid for 20 min and then rinsed with autoclaved tap water 3–4 times. Afterwards, the seeds were placed on wet filter paper in Petri dishes and transferred to the greenhouse for germination. Seedlings 2–4 cm in length were placed near the stems of tobacco at the 2–3 true leaf stage, whereas soybean was parasitized as soon as the plants reached ~10 cm in height. A low red/far-red light ratio, facilitating parasitism (Tada et al., 1996), was established using far-red (730-nm) LEDs with a photon flux density of 281.4 µmol m^{-2} s^{-1} in addition to the standard greenhouse lights until the first haustorial connection had developed.

Identification and validation of genomic and coding sequences. Genomic DNA was isolated from *C. campestris* using the NucleoSpin Plant II Kit (Machery Nagel, Düren, Germany) and the genes were amplified in overlapping fragments using Phusion high-fidelity polymerase (Thermo Fisher Scientific, Waltham, MA, USA) and the primers listed in Supplemental Table 5. Gene fragments were subcloned into pCRIITopo (Thermo Fisher Scientific) and sequenced, revealing the complete genomic sequence of *CcFT1* (11,502 bp) and *CcFT2* (12,819 bp). For the amplification of the full length cDNAs and to confirm correct splicing of *Cuscuta FT* mRNAs, the 3′-UTR was identified by 3′-RACE. RNA was isolated from *C. campestris* or *C. australis* haustoria using the PureLink RNA Mini Kit (Thermo Fisher Scientific) and reverse transcribed using Superscript II reverse transcriptase (Thermo Fisher Scientific) and the 3`Oligo_d(T) primer (Supplemental Table 5). The cDNAs were used as templates for PCR with Phusion high-fidelity polymerase and the GSP1 fw and 3`Adap (Supplemental Table 5). The PCR product was diluted 1:20 and used as a template for nested PCR with Phusion high-fidelity polymerase and the primers GSP2 fw and 3`Adap_nested (Supplemental Table 5). The amplicons were purified, subcloned into pCR™II-Topo™ (Thermo Fisher Scientific) and sequenced. Based on the identified 3′-UTR sequence, *CcFT1*, *CcFT2* and *CaFT* full-length coding sequences including parts of the 3′-UTR were amplified using the primers listed in Supplemental Table 5, subcloned into pCR™II-Topo™ (Thermo Fisher Scientific) and verified by sequencing. The coding sequences of *CcFT1* and *CaFT* were 534 bp in length and the coding sequence of *CcFT2* was 531 bp in length.

Quantitative real-time PCR. Plant tissues were harvested, snap-frozen in liquid nitrogen and ground using either an MM400 bead mill (Retsch, Haan, Germany) or with a mortar and pestle under liquid nitrogen. Tobacco, *Arabidopsis* and soybean RNA was extracted using the innuPREP Plant RNA kit

(Analytik Jena, Jena, Germany) and *Cuscuta* spp. RNA was extracted using the PureLink RNA Mini Kit. Residual genomic DNA was digested using the TURBO DNA-free kit (Thermo Fisher Scientific). Following reverse transcription using PrimeScript RT master mix (Takara Bio, San Jose, CA, USA), gene expression was analyzed by quantitative real-time PCR using Kapa SYBR Fast qPCR Master Mix (Merck, Darmstadt, Germany), the primers listed in Supplemental Table 5, and the CFX96 Real-Time System (Bio-Rad Laboratories, Hercules, CA, USA). Each reaction was carried out in technical triplicates. Specificity was ensured by melt curve analysis, the sequencing of PCR products, and by including no-template and no-reverse-transcription controls. Because residual host tissues may have been co-harvested with the haustoria, we also tested tobacco and soybean cDNA with the same primer sets (Supplemental Table 5). We did not detect any amplicons, confirming the primer specificity. Individual PCR efficiency was determined using LinReg PCR v2017.0 and relative gene expression levels were normalized to *EF1α* and *Actin* (*Cuscuta* spp.), *PP2* and *Ubi* (*A. thaliana*), *EF1α* (*N. tabacum*), or *F-Box* (*G. max*).

Cloning. For the targeted editing of *NtFT4*, the binary construct pDECAS9-*NtFT4*$_{\text{ex I-59.79 bp}}$ was cloned as previously described (Schmidt et al., 2020). The original plasmids were kindly provided by Holger Puchta (Karlsruhe Institute of Technology, Karlsruhe, Germany). For BiFC assays, *CcFT1*, *CcFT2* and *CcFD-like1* were amplified from cDNA using primers with attached restriction sites (Supplemental Table 5) and were transferred to pENTR4 (Thermo Fisher Scientific) by restriction and ligation. Subsequent transfer to pBatTL vectors was achieved by Gateway recombination (pBatTL plasmids were kindly provided by Joachim Uhrig and Guido Jach, University of Cologne, Cologne, Germany). BiFC constructs containing *NtFD1* and *NtFT5* were available from previous studies (Beinecke et al., 2018). For the heterologous expression of *CcFT* genes, we amplified the CaMV35S promoter sequence including the TMV Ω leader from pFGC5941-GW (www.chromdb.org) using the splicing by overlap extension (SOE)-PCR technique to eliminate the internal XhoI site. The amplicon was then transferred to the SmaI/XhoI sites of pLab12.10 (Xing et al., 2014) or pLab12.1 (Post et al., 2012) to generate pLab12.10Q35S-P and pLab12.1Q35S-P, respectively. The vector backbones carry different resistance cassettes, with pLab12.10 conferring kanamycin resistance and pLab12.1 conferring phosphinothricin resistance. *CcFT1* and *CcFT2* were amplified from cDNA using primers with attached restriction sites (Supplemental Table 5) and were transferred to pLab12.10Q35S-P and pLab12.1Q35S-P, respectively, by restriction and ligation.

***Agrobacterium*-mediated stable plant transformation.** For the generation of *Ntft4*$^{-}$*Ntft5*$^{-}$ double knockout plants, the previously described nullizygous T_2 progeny of *Ntft5*$^{-}$ mutant line #78 without the pDECAS9-*NtFT5*$_{\text{ex I-147.169bp}}$ transgene (Schmidt et al., 2020) was used for transformation. For functional complementation studies, *Ntft4*$^{-}$*Ntft5*$^{-}$ double knockout plants (T_2 generation) were used for transformation with binary plasmids carrying *CcFT1* or *CcFT2* under the control of the 35S promoter (Q35S-P). Transformation was carried out using the leaf disc method (Horsch et al.,1985) with *Agrobacterium tumefaciens* strain LBA4404 for heterologous gene expression, or EHA105 for CRISPR/Cas9-mediated genome editing. The appropriate binary vectors were introduced into these strains by electroporation. For the selection of transgenic plants, MS medium was supplemented with 100 mg/L kanamycin or 3 mg/L phosphinothricin, as appropriate. After callus regeneration and rooting in sterile culture medium, independent transgenic plant lines were cultivated in soil under LD conditions in the greenhouse as described above. Transgene integration was verified by PCR and, depending on the constructs, plants were either analyzed for edited targets (see below) or transgene expression levels were determined by qRT-PCR. For seed production, *Ntft4*$^{-}$*Ntft5*$^{-}$ double knockout scions were grafted onto SR1 wild-type stocks, whereas *Ntft4*$^{-}$*Ntft5*$^{-}$ double knockout plants overexpressing *CcFT1* or *CcFT2* flowered spontaneously. The T_1 progeny were used for further experiments.

For the generation of transgenic *A. thaliana* Col-0 plants, wild-type plants were transformed with *Agrobacterium tumefaciens* strain EHA105 carrying the binary plasmid pLab12.10Q35S-P::*CcFT1* using the floral dip method (Clough and Bent, 1998). For the selection of transgenic plants, ½ MS medium was supplemented with 25 mg/L kanamycin. Transgene integration was verified by PCR and independent transgenic plant lines were cultivated in soil under LD conditions in phytochambers. For phenotypic analysis, T_1-plants of three independent lines were cultivated in soil under SD conditions in

phytochambers. Transgene expression levels were determined by qRT-PCR using the primers listed in Supplemental Table 5.

Identification and screening of genome-edited plants. The *NtFT5* locus in the mutant plants (*Ntft5*$^{-}$ and *Ntft4*$^{-}$*Ntft5*$^{-}$) was analyzed by isolating genomic DNA using the NucleoSpin Plant II Kit and amplifying exon I using Phusion high-fidelity polymerase and the primers listed in Supplemental Table 5. The amplicon was verified by sequencing. After regenerating *Ntft5*$^{-}$ plants transformed with pDECAS9-*NtFT4*$_{\text{ex I-59.79 bp}}$, transgene integration was verified by the isolation of genomic DNA followed by PCR using the 2x *i*-MAX Mastermix (INtRON Biotechnology, Seongnam, Korea) and the primers listed in Supplemental Table 5. For the analysis of genome editing in T_0 plants, parts of exon I were amplified using Phusion high-fidelity polymerase and the primers listed in Supplemental Table 5. The amplicons were transferred to pCRIITopo and sequenced. Promising plants were cultivated for seed production and the T_1 progeny were analyzed to confirm the knockout mutations. For the analysis of genome editing in T_1 plants, parts of exon I were amplified using the Phusion high-fidelity polymerase and the primers listed in Supplemental Table 5. The amplicons were either transferred to pCRIITopo for sequencing (if the plants carried different mutations in their two *NtFT4* alleles) or were sequenced directly (if the same mutation was present in both alleles).

Phenotyping. For tobacco, we counted the number of days from seed sowing to full opening of the first floral bud, and the number of leaves when the first floral bud fully opened. For *Cuscuta* spp., we counted the number of days from the formation of the first haustorial connection until the first floral bud had fully opened. For *Arabidopsis*, we counted the number of days from seed sowing to full opening of the first floral bud as well as the number of rosette leaves and cauline leaves when the first floral bud fully opened.

Infiltration of *Nicotiana benthamiana*. For transient expression of BiFC constructs, *A. tumefaciens* strain GV3101 pMP90 was transformed with the corresponding binary vectors by electroporation. *N. benthamiana* plants were cultivated in the greenhouse (16-h photoperiod) until they were 4–5 weeks old. The leaves were then infiltrated with *A. tumefaciens* strain GV3101 pMP90 carrying the appropriate plasmids (OD_{600} = 0.25) and *A. tumefaciens* strain C58C1 (OD_{600} = 0.3) carrying the pCH32 helper plasmid and the pBin61 plasmid encoding the RNA silencing suppressor p19 from tomato bushy stunt virus (Walter et al., 2004). Plants were cultivated under continuous light for 3–4 days, and leaf discs were screened for fluorescent cells in the abaxial epidermis.

Microscopy. For BiFC experiments, fluorescence was analyzed using a STELLARIS 8 confocal laser scanning microscope (Leica Microsystems, Wetzlar, Germany) at excitation/emission wavelengths of 549/569–629 nm for reconstituted mRFP. Interaction was confirmed if at least five independent images showing fluorescence were captured.

Bioinformatic recovery of flowering time genes. We extracted the full set of potentially functional flowering genes and corroborated gene losses in *C. campestris* and *C. australis* based on the genomic sequences of both species from NBCI GenBank (Bioprojects: PRJEB19879 and PRJNA394036), in addition to assembling and querying RNA-Seq data covering key developmental stages (Supplemental Table 2). Genome data alongside published annotations of 13 closely related eudicot species (*Ipomoea triloba* – PRJNA574454; *Beta vulgaris* – assembly EL10_1.0; *Spinacia oleraceae* – assembly ASM200726v1; *N. tabacum* – assembly Ntab-TN90; *N. sylvestris* – assembly Nsyl; *N. tomentosiformis* – assembly Ntom_v01; *Vaccinium darrowii* – assembly USDA_Vadar_1.0_pri; *Solanum lycopersicum* – assembly SL3.1; *Solanum tuberosum* – assembly SolTub_3.0; *Erythranthe guttata* – assembly Mimgu1_0; *Olea europaea* var. *sylvestris* – assembly O_europaea_v1; *Lactuca sativa* – assembly Lsat_Salinas_v8 and *Daucus carota* subsp. *sativus* – assembly ASM162521v1) were included for comparative evolutionary analysis of gene conservation. To account for differences in the quality of genomic data depending on the annotation version, we used a two-stage flowering time gene analysis in which a primary query for flowering time genes in all species was applied to the raw (annotation-free) genomic assembly and hits were then cross-validated and refined using readily available gene annotations/predictions. The two *Cuscuta* species differ significantly in their coding capacity (Vogel et al., 2018; Sun et al., 2018; Lyko and Wicke, 2021), so we also reciprocally searched their genomes for missing protein-coding genes using blastX (e-value cutoff = 10^{-3}) and exonerate (protein2genome mode; min length coverage 70%), and compared these data to the closest, nonparasitic relative *Ipomoea triloba*, which belongs to the same family as *Cuscuta* (Convolvulaceae).

This approach retrieved many originally "missing" genes (DRYAD data repository, https://datadryad.org/stash/share/DK8Olh2VqFwbGNL0GtkGt24dD0GhWhJn82oLBC1XK70), so we used the BRAKER pipeline to re-annotate *Cuscuta* spp. and *Ipomoea nil* (assembly Asagao_1.1; Bioprojects: PRJNA344313, PRJDB4356) for a direct comparison of the new *ab initio* gene model predictions. Training Augustus in the BRAKER pipeline followed the steps described for *C. campestris* (Vogel et al., 2018), whereby we also used the odp10 protein database plus *I. nil* and *I. triloba* proteins. Our annotation revealed an almost perfect 2:1 gene annotation ratio between *C. campestris* and *C. australis*. Given that an analysis of syntenic blocks across *Cuscuta australis*, *C. campestris* and *Ipomoea triloba* computed using the Doerr & Moret (2017) pipeline found counterparts with conserved genomic orientation on all genomic fragments longer than three million bases in both parasite species (DRYAD:https://datadryad.org/stash/share/DK8Olh2VqFwbGNL0GtkGt24dD0GhWhJn82oLBC1XK70) we conclude that the genome assemblies may be available in a collapsed vs. uncollapsed allelic/pseudochromosomal configuration, rather than reflecting a ploidization event since the parasite species diverged. For the purpose of screening for flowering genes, we merged these new gene sets with the corresponding original ones and removed redundancy in cases of perfect matches. The genomic sets were used to seed-search for 295 protein-coding genes implicated in flowering time regulation using *exonerate* in protein2genome mode (https://www.ebi.ac.uk/about/vertebrate-genomics/software/exonerate), allowing for introns between 15 bp and 35 kb. Reference flowering time proteins were obtained from the FLOR-ID database http://www.phytosystems.ulg.ac.be/florid/ (Bouché et al., 2016) and the Araport11 genome release of *Arabidopsis thaliana* (https://www.arabidopsis.org/index.jsp). We excluded all 11 miRNAs involved in flowering time regulation. In addition, the exonerate-based search for flowering time genes using the Arabidopsis reference gene set was repeated for *C. australis* and *C. campestris* transcriptome assemblies, using all RNA-Seq datasets summarized in Supplemental Table 2. *Cuscuta* spp. transcriptomes were assembled *de novo* using the Trinity RNA-Seq pipeline with default settings (Haas et al., 2013). Recovered hit sequence seeds were then used to extend the region bidirectionally by iteratively mapping genomic and transcriptomic sequence data using *bowtie*2, whereby up to 10 iterations were performed. The exonerate wrapper and all retrieved seed sequence alignments are available in DRYAD: https://datadryad.org/stash/share/DK8Olh2VqFwbGNL0GtkGt24dD0GhWhJn82oLBC1XK70. To compare the divergence in sequence conservation of *Cuscuta* spp. flowering time genes with the corresponding homologous gene models of 13 other eudicot species, we ran *exonerate* in protein2genome mode (as above). Matched protein-coding gene regions were extracted, aligned using *mafft* in auto mode (Katoh and Standley, 2013), and evaluated for the highest-scoring coding sequence length and sequence divergence, relative to functionally validated *Arabidopsis* references (% conservation of gene models). Finally, we used phylogeny-aware domain-based examination, for which we extracted the full gene models matching reference FD and FT sequences from the combined genomic annotations. We built FT and FD alignments using *mafft* in e-ins-i mode with a gap penalty of 1.93. From these data, we reconstructed the protein relationships with RAxML (Stamatakis, 2014) with 200 bootstrap replicates, using the WAG model of amino acid substitution with a γ rate distribution.

Transposable element analysis. To check whether transposable element insertion may have rendered FT or FD genes nonfunctional, we used domain-based annotation of transposable elements (DANTE; https://github.com/kavonrtep/dante). We extracted the extended gene regions (–20 kbp and +20 kbp of the entire genes of both species), allowing us to retrieve full-length mobile DNA elements, if any. DANTE was run with Viridiplantae 3.0 as the taxon and protein database and a BLOSUM80 scoring matrix. Single hits were retained if they scored at least 80% similarity to database records. DANTE's built-in *Protein Domain Filter* tool was then used on the full DANTE annotation and only domains with at least 35% protein sequence identity between input and mapped protein from the database, 45% minimum similarity, and 80% minimum alignment length were considered. We filtered hits with more than three interruptions, such as frameshifts and/or stop codons and insertions resulting in a domain length extension of 1.2 between the new and the reference domain from the database. The resulting filtered and unfiltered evidence for transposable elements was extracted from GFF3 files.

RNA-Seq gene expression analysis. We extracted the full coding sequence models of our candidate FD and FT sequences (and for comparison, those of the FT-interacting genes SOC1, CO, LFY, TFL1, and GI) from the combined genomic annotations of both *Cuscuta* species. We assessed gene expression of FT, FD and our selected interacting proteins by mapping all available RNA-Seq data

(Supplemental Table 2) using *bowtie2* in local mode (Langmead and Salzberg, 2012). The unsorted read alignments were parsed to calculate per-reference base coverage using the *pileup* function of the BBMap program suite (Bushnell, 2014; https://github.com/BioInfoTools/BBMap). RNA-Seq expression levels were extremely low for FT (Supplemental Table 1), so its mapping results were double-checked and confirmed by qRT-PCR using highly discriminating primers (Supplemental Table 5).

Statistics. All boxplots in the figures were prepared in OriginPro2022 (OriginLab Corporation, Northampton, MA, USA) using default settings (center line = median; square = mean, box limits = upper and lower quartiles; whiskers = 1.5× interquartile range; diamonds = outliers). Statistical analysis was carried out using OriginPro2022. Equality of variances was determined by one-way analysis of variance (ANOVA), and pairwise comparisons were assessed using Tukey's *post hoc* test for multiple comparisons and Student's *t*-test for single pairwise comparisons, unless stated otherwise. If ANOVA revealed significant differences in variances, pairwise comparisons were assessed using ANOVA with Welch´s correction and Games-Howell *post hoc* test for multiple comparisons or Student's *t*-test with Welch´s correction for single pairwise comparisons. Spearman rank correlation tests were conducted in *R* as one-sided tests with alpha (significance level) set to 0.05 (5%), highlighting the strong expectation that higher FT-expression is associated with earlier flowering, therefore expecting a negative correlation with true *r*s being less than 0.

Supplemental references

Beinecke, F. A., Grundmann, L., Wiedmann, D. R., Schmidt, F. J., Caesar, A. S., Zimmermann, M., Lahme, M., Twyman, R. M., Prüfer, D., Noll, G. A. (2018) The FT/FD-dependent initiation of flowering under long-day conditions in the day-neutral species *Nicotiana tabacum* originates from the facultative short-day ancestor *Nicotiana tomentosiformis*. *The Plant Journal* **96**(2):329-342. https://doi.org/10.1111/tpj.14033

Bouché, F., Lobet, G., Tocquin, P., Périlleux, C. (2016) FLOR-ID: an interactive database of flowering-time gene networks in *Arabidopsis thaliana*. *Nucleic Acids Res* **44**:D1167-1171. https://doi.org/10.1093/nar/gkv1054

Bushnell, B. (2014) BBMap: A Fast, Accurate, Splice-Aware Aligner. https://www.osti.gov/servlets/purl/1241166.

Chia, T.Y.P., Müller, A., Jung, C., and Mutasa-Göttgens, E.S. (2008) Sugar beet contains a large CONSTANS-LIKE gene family including a CO homologue that is independent of the early-bolting (B) gene locus. *J. Exp. Bot.* **59**(10): 2735–2748. https://doi.org/10.1093/jxb/ern129

Cho L. H., Pasriga R., Yoon J., Jeon J. S., An G. (2018) Roles of sugars in controlling flowering time. *J. Plant Biol.* 2018; **61**: 121-130. https://doi.org/10.1007/s12374-018-0081-z

Clough S.J., Bent A.F. (1998) Floral dip: a simplified method for *Agrobacterium*-mediated transformation of *Arabidopsis thaliana*. *The Plant Journal* **16**(6):735-743. https://doi.org/10.1046/j.1365-313x.1998.00343.x

Dally, N., Eckel, M., Batschauer, A., Höft, N., and Jung, C. (2018) Two CONSTANS-LIKE genes jointly control flowering time in beet. *Sci. Rep.* **8**(1): 16120. https://doi.org/10.1038/s41598-018-34328-4

Doerr, D., Moret, B. M. E. (2018) Sequence-based synteny analysis of multiple large genomes. In *Comparative Genomics, Methods in Molecular Biology*., Setubal, J. C., Stoye, J., Stadler, P. F., Eds. (Springer New York), pp. 317–329.

Haas, B. J., Papanicolaou, A., Yassour, M., Grabherr, M., Blood, P. D., Bowden, J., ... & Regev, A. (2013). De novo transcript sequence reconstruction from RNA-seq using the Trinity platform for reference generation and analysis. *Nat. Protoc.* **8**(8):1494-1512. https://doi.org/10.1038/nprot.2013.084

Horsch, B., Fry, J. E., Hoffmann, N. L., Wallroth, M., Eichholtz, D., Rogers, S. G., Fraley, R.T. (1985) A simple and general method for transferring genes into plants. *Science* **227**:1229–1231. https://doi.org/doi.org/10.1126/science.227.4691.1229

Katoh, K., and Standley, D. M. (2013) MAFFT multiple sequence alignment software version 7: improvements in performance and usability. *Mol. Biol. Evol.* **30**:772–780. https://doi.org/10.1093/molbev/mst010

Langmead, B., and Salzberg, S. L. (2012) Fast gapped-read alignment with Bowtie 2. *Nat. Methods* **9:**357–359. https://doi.org/10.1038/nmeth.1923

Libault, M., Thibivilliers, S., Bilgin, D. D., Radwan, O., Benitez, M., Clough, S. J., Stacey, G. (2008) Identification of four soybean reference genes for gene expression normalization. *The Plant Genome*, *1*(1). https://doi.org/10.3835/plantgenome2008.02.0091

Loo S. W. (1946) Cultivation of excised stem tips of dodder in vitro. *Am. J. Bot.* **33**(4): 295–300. https://doi.org/10.2307/2437436

Lyko, P., Wicke, S. (2021) Genomic reconfiguration in parasitic plants involves considerable gene losses alongside global genome size inflation and gene births. *Plant Physiol.* **186:**1412–1423. https://doi.org/10.1093/plphys/kiab192

Navarro, C., Cruz-Oró, E., and Prat, S. (2015) Conserved function of FLOWERING LOCUS T (FT) homologues as signals for storage organ differentiation. *Curr. Opin. Plant Biol.* **23**: 45–53. https://doi.org/10.1016/j.pbi.2014.10.008

Pin, P.A., Benlloch, R., Bonnet, D., Wremerth-Weich, E., Kraft, T., Gielen, J.J.L., and Nilsson, O. (2010) An antagonistic pair of FT homologs mediates the control of flowering time in sugar beet. *Science* **330** (6009): 1397–1400. https://doi.org/10.1126/science.1197004

Pin, P.A., Zhang, W., Vogt, S.H., Dally, N., Büttner, B., Schulze-Buxloh, G., Jelly, N.S., Chia, T.Y.P., Mutasa-Göttgens, E.S., and Dohm, J.C., et al. (2012) The role of a pseudo-response regulator gene in life cycle adaptation and domestication of beet. *Curr. Biol.* CB **22** (12): 1095–1101. https://doi.org/10.1016/j.cub.2012.04.007

Post, J.J., Eisenreich, W., Huber, C., Twyman, R.M., Prüfer, D. and Schulze Gronover, C. (2013) Establishment of an ex vivo laticifer cell suspension culture from *Taraxacum brevicorniculatum* as a production system for cis-isoprene. *J. Mol. Catal. B Enzym* **103**:85-93. https://doi.org/doi.org/10.1016/j.molcatb.2013.07.013

Ream T. S., Woods D. P., Amasino R. M. (2012) The molecular basis of vernalization in different plant groups. *Cold Spring Harb. Symp. Quant. Biol.* **77**: 105-15. https://doi.org/10.1101/sqb.2013.77.014449

Rezaei, H., Alamisaeed, K., Moslemkhani, C. (2017) Overexpression of stress-related genes in *Cuscuta campestris* in response to host defense reactions. *BioTechnologia* **98**(2):131-139 https://doi.org/10.5114/bta.2017.68312

Schmidt, F. J., Zimmermann, M. M., Wiedmann, D. R., Lichtenauer, S., Grundmann, L., Muth, J., Twyman, R. M., Prüfer, D., Noll, G. A. (2020) The major floral promoter NtFT5 in tobacco (*Nicotiana tabacum*) is a promising target for crop improvement. *Front. Plant Sci.* 1666. https://doi.org/10.3389/fpls.2019.01666

Shen G., Liu N., Zhang J., Xu Y., Baldwin I. T., Wu, J. (2020) *Cuscuta australis* (dodder) parasite eavesdrops on the host plants' FT signals to flower. Proc. Natl. Acad. Sci. U S A. 117(37):23125-23130. https://doi.org/10.1073/pnas.2009445117

Stamatakis, A. (2014) RAxML version 8: a tool for phylogenetic analysis and post-analysis of large phylogenies. *Bioinformatics* **30:**1312–1313. https://doi.org/10.1093/bioinformatics/btu033

Sun, G., Xu, Y., Liu, H., Sun, T., Zhang, J., Hettenhausen, C., Shen, G., Qi, J., Li, J., Wang, L., Chang, W., Guo, Z., Baldwin, I.T., Wu, J. (2018) Large-scale gene losses underlie the genome evolution of parasitic plant *Cuscuta australis*. *Nat. Commun.* **9**(1):2683. https://doi.org/10.1038/s41467-018-04721-8

Tada, Y., Sugai, M., Furuhashi, K. (1996) Haustoria of *Cuscuta japonica*, a holoparasitic flowering plant, are induced by the cooperative effects of far-red light and tactile stimuli. *Plant Cell Physiol.* **37**:1049–1053. https://doi.org/10.1093/oxfordjournals.pcp.a029052

Vogel, A., Schwacke, R., Denton, A. K., Usadel, B., Hollmann, J., Fischer, K., Bolger, A., Schmidt, M.H.-W., Bolger, M.E., Gundlach, H., Mayer, K.F.X., Weiss-Schneeweiss, H., Temsch, E.M, Krause, K. (2018) Footprints of parasitism in the genome of the parasitic flowering plant *Cuscuta campestris*. *Nat. Commun.* **9**(1):2515. https://doi.org/10.1038/s41467-018-04344-z

Vogt, S.H., Weyens, G., Lefèbvre, M., Bork, B., Schechert, A., and Müller, A.E. (2014) The FLC-like gene BvFL1 is not a major regulator of vernalization response in biennial beets. *Front. Plant Sci.* **5**: 146. https://doi.org/10.3389/fpls.2014.00146

Walter, M., Chaban, C., Schütze, K., Batistic, O., Weckermann, K., Näke, C., Blazevic, D., Grefen, C., Schumacher, K., Oecking, C., Harter, K., Kudla, J. (2004) Visualization of protein interactions in living plant cells using bimolecular fluorescence complementation. *The Plant Journal* **40**(3):428-438. https://doi.org/10.1111/j.1365-313X.2004.02219.x

Xing, S., van Deenen, N., Magliano, P., Frahm, L., Forestier, E., Nawrath, C., Schaller, H., Schulze Gronover, C., Prüfer, D., Poirier, Y. (2014) ATP citrate lyase activity is post-translationally regulated by sink strength and impacts the wax, cutin and rubber biosynthetic pathways. *The Plant Journal* **79**(2):270-284. https://doi.org/10.1111/tpj.12559

Zhang, D., Qi, J., Yue, J., Huang, J., Sun, T., Li, S., Wen, J.-F., Hettenhausen, C., Wu, J., Wang, L., Zhuang, H., Wu, J., Sun, G. (2014) Root parasitic plant Orobanche aegyptiaca and shoot parasitic plant *Cuscuta australis* obtained Brassicaceae-specific strictosidine synthase-like genes by horizontal gene transfer. *BMC plant biology*, **14**(1):1-14. https://doi.org/10.1186/1471-2229-14-19

Zimmermann, M.M., Grundmann, L., Känel, A., Schwarze, A., Wiedmann, D.R., Muth, J., Twyman, R.M., Prüfer, D., and Noll, G.A. (2022) Unraveling the mystery behind the short-day-specific flowering of tobacco cultivar Maryland Mammoth. *bioRxiv*. https://doi.org/10.1101/2022.06.27.497884

Supplementary Figures

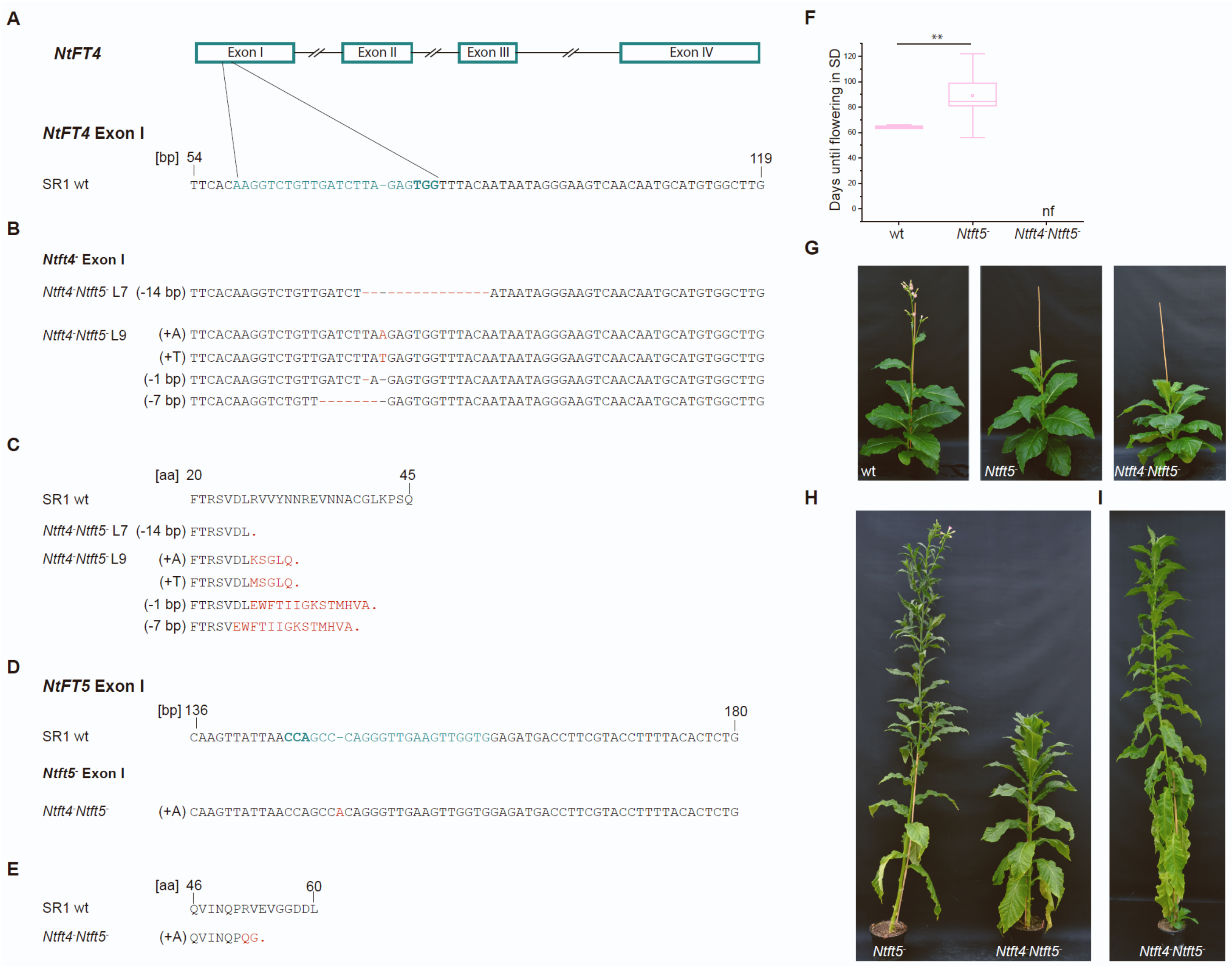

Supplemental Figure 1. Molecular and phenotypic analysis of *Ntft4*$^{-}$*Ntft5*$^{-}$ double knockout plants. A) Location of the *NtFT4*-specific protospacer (teal) and the protospacer adjacent motif (PAM; bold) in the 59–82 bp region of exon I of wild-type SR1 plants (SR1 WT). Exons are shown as boxes, introns as lines. **B)** PCR-based screening of two independent transgenic lines (L7, L9) for mutated allelic variants of *NtFT4*. Amplicons were subcloned and sequenced. Insertions and deletions are highlighted in red letters. L7 progeny plants possess a homozygous 14-bp deletion, whereas in L9 progeny plants the two *NtFT4* alleles each harbor one of four different mutations. **C)** Sequence alignment of native NtFT4 and the protein variants encoded by the mutated alleles. Amino acid substitutions and premature stop codons are highlighted in red letters. **D)** Knockout of NtFT5 (Schmidt et al., 2020) by a single base pair insertion at position 153 in exon I, leading to a frameshift and a premature stop codon, as validated by PCR and direct sequencing of the PCR product. **E)** Sequence alignment of native NtFT5 and the protein variant encoded by the mutated allele. Amino acid substitutions and premature stop codon are highlighted in red letters. **F)** Flowering time of SR1 wild-type plants (WT), *Ntft5*$^{-}$ plants and *Ntft4*$^{-}$*Ntft5*$^{-}$ plants. Tobacco is a day-neutral plant that produces two FT floral inducers (NtFT4 and NtFT5). NtFT5 is predominantly responsible for floral induction under LD conditions, whereas both NtFT4 and NtFT5 induce flowering under short-day (SD) conditions. Under SD conditions, SR1 wild-type plants flowered on average after 64 days, whereas *Ntft5*$^{-}$ plants, which carry homozygous knockout mutations in *NtFT5* but still express *NtFT4* under SD conditions, flowered on average after 89 days. *Ntft4*$^{-}$*Ntft5*$^{-}$ plants, lacking both floral activators, did not flower at all under SD conditions up to the end of the experiment (122 days after sowing). Days until flowering were determined when the first bud had fully opened. ** Statistically significant difference as determined using Student's *t*-test with Welch's correction, $p < 0.01$ ($n = 10$). In the boxplots, center line = median, square = mean, box limits = upper and lower quartiles, and whiskers = 1.5× interquartile range. **G)** Phenotypes of WT, *Ntft5*$^{-}$, and *Ntft4*$^{-}$*Ntft5*$^{-}$ plants cultivated under SD conditions 65 days after sowing. **H)** Phenotypes of *Ntft5*$^{-}$ and *Ntft4*$^{-}$*Ntft5*$^{-}$ plants cultivated under SD conditions 120 days after sowing. **I)** Phenotype of *Ntft4*$^{-}$*Ntft5*$^{-}$ plant cultivated under LD conditions 139 days after sowing.

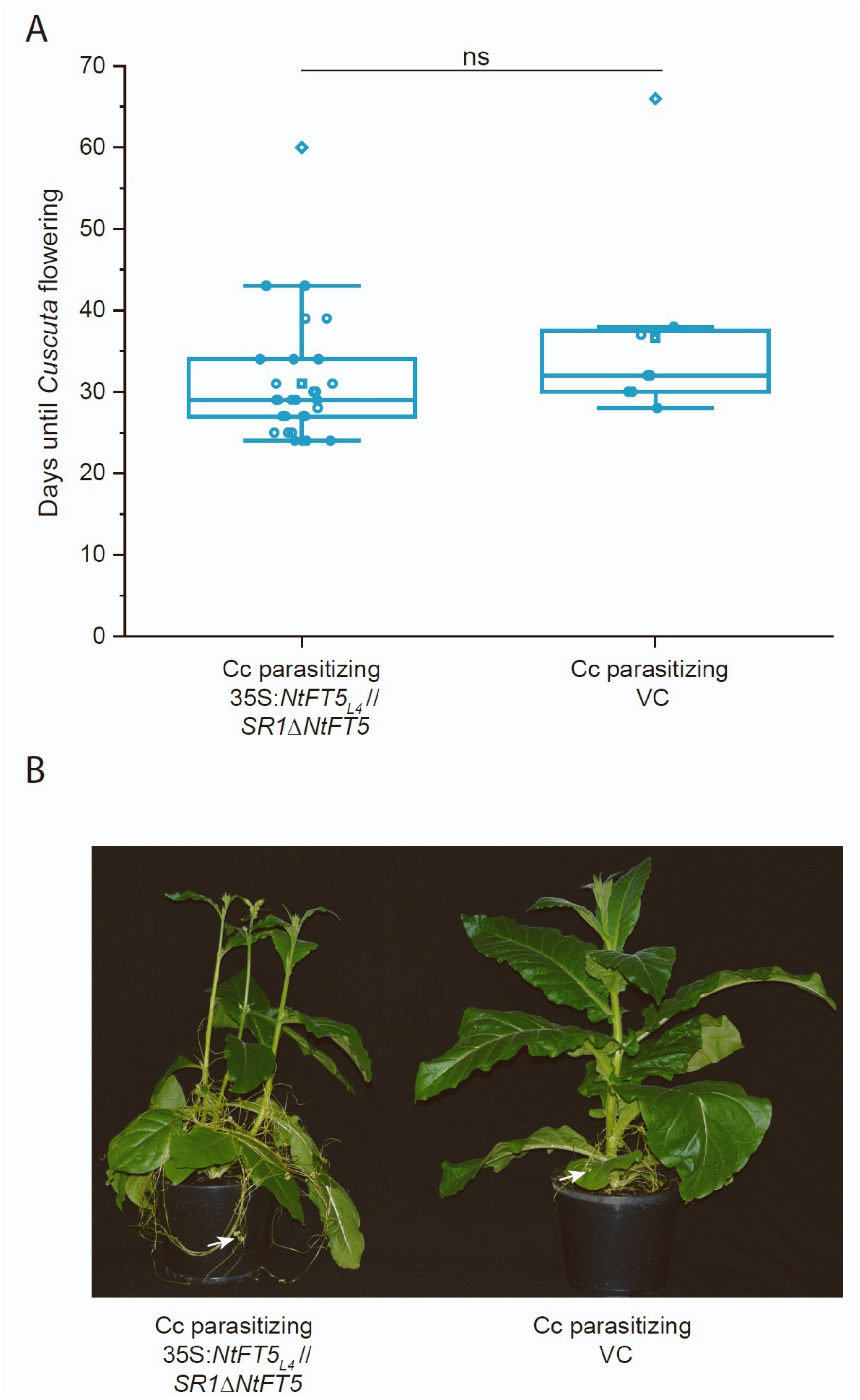

Supplemental Figure 2. Impact of NtFT5 overexpression in the tobacco host on flowering time of *C. campestris*. *C. campestris* (Cc) was allowed to parasitize 35S:*NtFT5*$_{L4}$//SR1Δ*NtFT5* plants, whose flowering time is reduced by approx. 20% compared to vector controls (VC) (Zimmermann et al., 2022). **A)** Elevated *NtFT5* expression levels in the host did not influence *Cuscuta* flowering time as determined by Student´s *t*-test (ns = non-significant), *n* = 29 (35S:*NtFT5*$_{L4}$//SR1Δ*NtFT5*); *n* = 8 (VC). In the boxplots, center line = median, square = mean, box limits = upper and lower quartiles, whiskers = 1.5× interquartile range, and diamonds = outliers. **B)** Flowering phenotype of *C. campestris* parasitizing 35S:*NtFT5*$_{L4}$//SR1Δ*NtFT5* or VC. *Cuscuta* flowers are indicated exemplarily by white arrows. The photograph was taken 79 days after tobacco seed sowing.

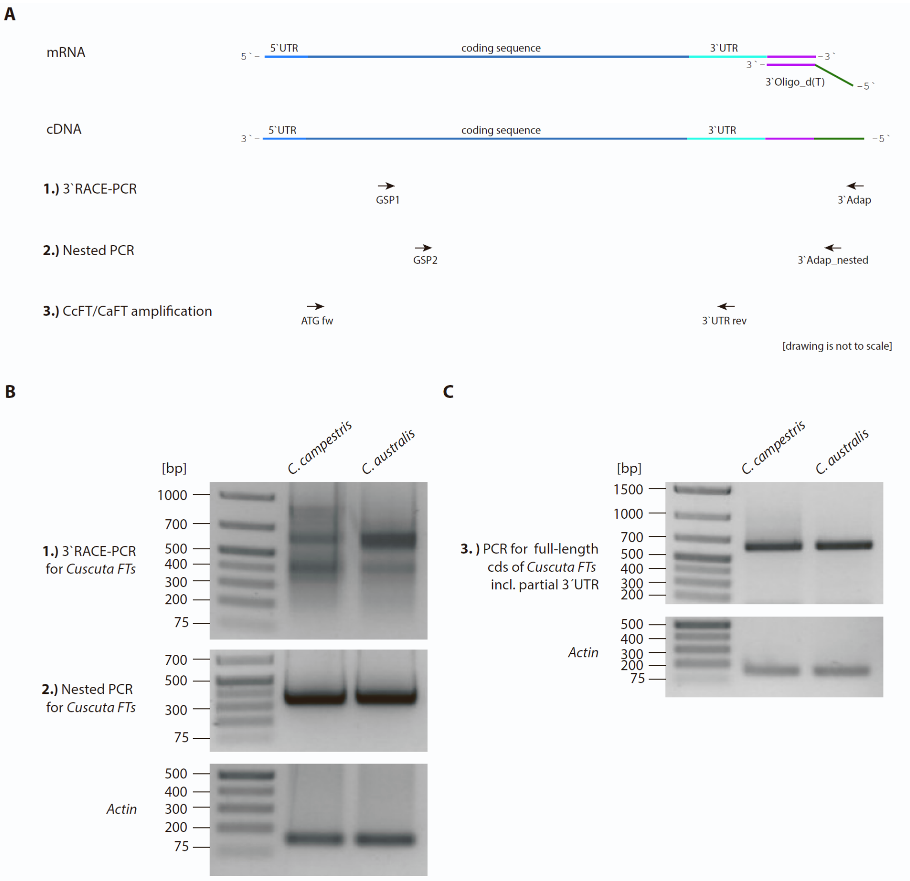

D

CcFT1

ATGGCGGCGAGGCGGCAGAGAGACGCTCTGGCTGTTGGGCGCGTGATCGGAGAGGTGCTTGAGCCTTTTACGGGCTCCGTAAACCTCAGGGTGGCTT
ACGGCGACCGCACCGTCACCAACGGCTGCGAGCTCCGGCCATCTCTCGTGCTCAACCCGCCGAGGGTCGACATCGGCGGCCATGATCTACGCACTTT
CTATACTCTCGTGATGGTGGATCCTGATGCTCCAAGTCCAAGTGATCCAAACTTAAGAGAATACTTGCACTGGTTGGTGACTGACATTCCAGCGAGC
ACCGGAGTTAATTTTGGGCATGAAATGGTATGCTATGAAAGCCCGCAACCAATCGTCGGAATCCACCGCTTCGTATTCGTACTGTTCCAGCAGATGG
GCCGACAGACCGTTTTCGCCCCGGGTTGGCGCCAAAATTTCAGCACTCGAGAATTCGCCGAGGTTCACAACCTCGGTCCACCGGTCGCGGCGATCTT
CTTTAACGGTCAACGAGAGGCGGGCACCGGCGGCAGGCGCCGTCAATAG*GAAATTCTTTGACTCCGACGTAGTCGTAGTTTATGTATGTACCGATGT*
*ACGTGTTGAGAACAATAATCCACCGTGCGCGACGGCTAGTTTCCACAATAAATATAATAATAATTCCAATTAGTG***AAAAAAAAAAAAAAAAAAAACA**
CTGTCATGCCGTTACGTAGCG

CaFT1

ATGGCGGCGAGGCGGCAGAGAGACGCTCTGGCTGTTGGGCGCGTGATCGGAGAGGTGCTTGAGCCTTTTACGGGCTCCGTAAACCTCAGGGTGGCTT
ACGGCGACCGCACCGTCACCAACGGCTGCGAGCTCCGGCCATCTCTCGTGCTCAACCCGCCGAGGGTCGACATCGGCGGCCATGATCTACGCACTTT
CTATACTCTCGTGATGGTGGATCCTGATGCTCCAAGTCCAAGTGATCCAAACTTAAGAGAATACTTGCACTGGTTGGTGACTGACATTCCAGCGAGC
ACCGGAGTTAATTTTGGGCATGAAATGGTATGCTATGAAAGCCCGCAACCAATCGTCGGAATCCACCGCTTCGTATTCGTACTGTTCCAGCAGATGG
GCCGACAGACCGTTTTCGCCCCGGGTTGGCGCCAAAATTTCAGCACTCGAGAATTCGCCGAGGTTCACAACCTCGGTCCACCGGTCGCGGCGATCTT
CTTTAACGGTCAACGAGAGGCGGGCACCGGCGGCAGGCGCCGTCAATAG*GAAATTCTTTGACTCCGACGTAGTCGTAGTTTATGTATGTACCGATGT*
*ACGTGTTGAGAACAATAATCCACCGTGCGCGGCGGCTAGTTTCCACAATAAATATAATAATAATTCCAATTAGTG***AAAAAAAAAAAAAAAAAAAAAA**
AAACACTGTCATGCCGTTACGTAGCG

CcFT2

ATGGCGGCGAGGCGGCAGAGAGACGCTCTGGCAGTTGGGCGCGTGATCGGAGAGGTGCTGGAGCCATTTACGGGCTCCATAAACCTCAGGGTGGCTT
ACGGCGATCGCACCGTCACCAACGGCTGCGAGCTCCGGCCCTCTCTCGTGCTCCACCCGCCGAGGGTCGACATCGGCGGCCATGATCTACGCACTTT
CTATACTCTCGTGATGGTGGATCCTGATGCTCCAAGTCCAAGTGATCCAAACTTAAGAGAATACTTGCACTGGTTGGTGAGTGACATTCCAGCGAGC
ACCGGAGTTAATTTTGGGCATGAGATTGTATGTTATGAAAGCCCACAGCCAATCGTCGGAATCCACCGGTTCGTATTTGTACTGTTCCAGCAGATGG
GCCGACAGACCGTTTTCGCCCCGGGTTGGCGCCAAAATTTCAGCACTCGAGAATTCGCCGAGGTTCACAACCTCGGTCCACCGGTGGCGGCGATCTT
CTTTAACGGTCAACGAGAGGCGGGCACCGGAGGCAGGCGCCGTTAA*TAGGAAATTCTTTGACTCCGACGTAGTCGTAGTTTATGTATGTACCGATGT*
ACGTACGTATTGAGAACAATAATCCACCGTGCGC

Supplemental Figure 3. Identification of full-length coding sequences and 3`UTR of *Cuscuta FT* homologs.** **A)** Schematic drawing of the applied RACE-PCR strategy. GSP=gene specific primer; 3´Adap=adaptor primer; 3´Adap_nested = nested adaptor primer. The potential 5`UTR is shown in blue, coding sequence is shown in teal, 3`UTR is shown in turquoise, Poly(A)-tail and complementary Oligo d(T) are shown in purple and the adaptor sequence is shown in dark green. **B)** After cDNA synthesis using the 3`Oligo_d(T) primer with an overhanging adaptor sequence, the 3`RACE-PCR was carried out using a *CcFT1/CaFT*-specific primer (GSP1) and a primer specifically binding to the adaptor sequence (3´Adap), resulting in several unspecific bands. The PCR product was diluted 1:20 and used as a template for the second PCR using a nested *CcFT1*/*CaFT*-specific primer (GSP2) and a nested adaptor primer (3´Adap_nested), resulting in specific bands, which were purified, subcloned and sequenced. **C) Amplification of the full-length sequences of *CcFT1*/*CaFT* and *CcFT2* was carried out using cDNA as template and primers spanning the start codon and parts of the 3`UTR. The PCR products were purified, subcloned and sequenced. **D)** Sequence of the full-length *CcFT1, CaFT* and *CcFT2* coding sequences and their 3´UTRs (partial 3´UTR in case of *CcFT2*). The coding sequences are shown in regular letters and the (partial) 3`UTR sequences are shown in italic letters. The Poly(A) tails of *CcFT1* and *CaFT* are shown in bold letters. The 3´Adap_nested primer is indicated by underlined bold letters, while the 3´UTR primer is indicated by underlined italic letters.

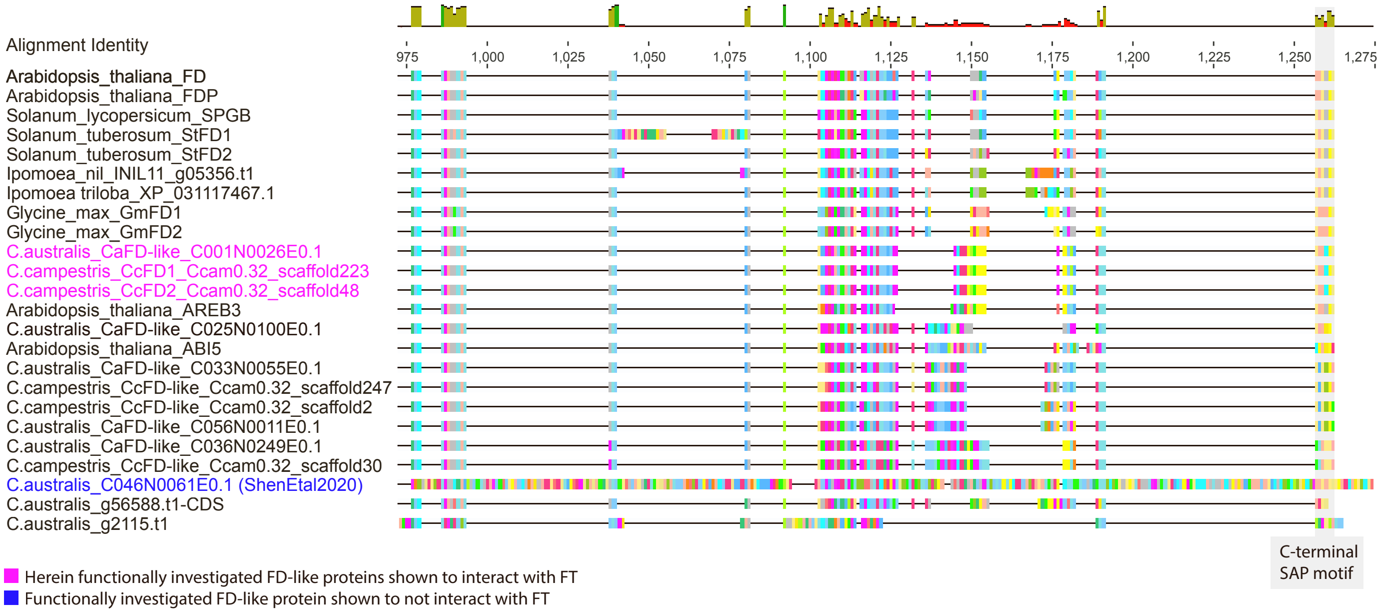

Supplemental Figure 4. SAP-motif region of FD-like proteins. Visualization of aligned sequences at the C-terminus of FD-like proteins, showing the presence of a SAP-like motif in the newly identified and functionally tested CcFD1, CcFD2 and CaFD-like genes (magenta), for which we showed an interaction with endogenous *Cuscuta* FT proteins. Within the alignment block, same-colored boxes indicate the same amino acids. For comparison, functionally validated FD and FD-related proteins from *Arabidopsis thaliana* and other angiosperms are included. Highlighted in blue is the FD protein sequence reported not to interact with endogenous *Cuscuta* FT (Shen et al., 2020). The complete alignment is published in reusable Nexus format in the DRYAD Data Repository, https://datadryad.org/stash/share/DK8Olh2VqFwbGNL0GtkGt24dD0GhWhJn82oLBC1XK70

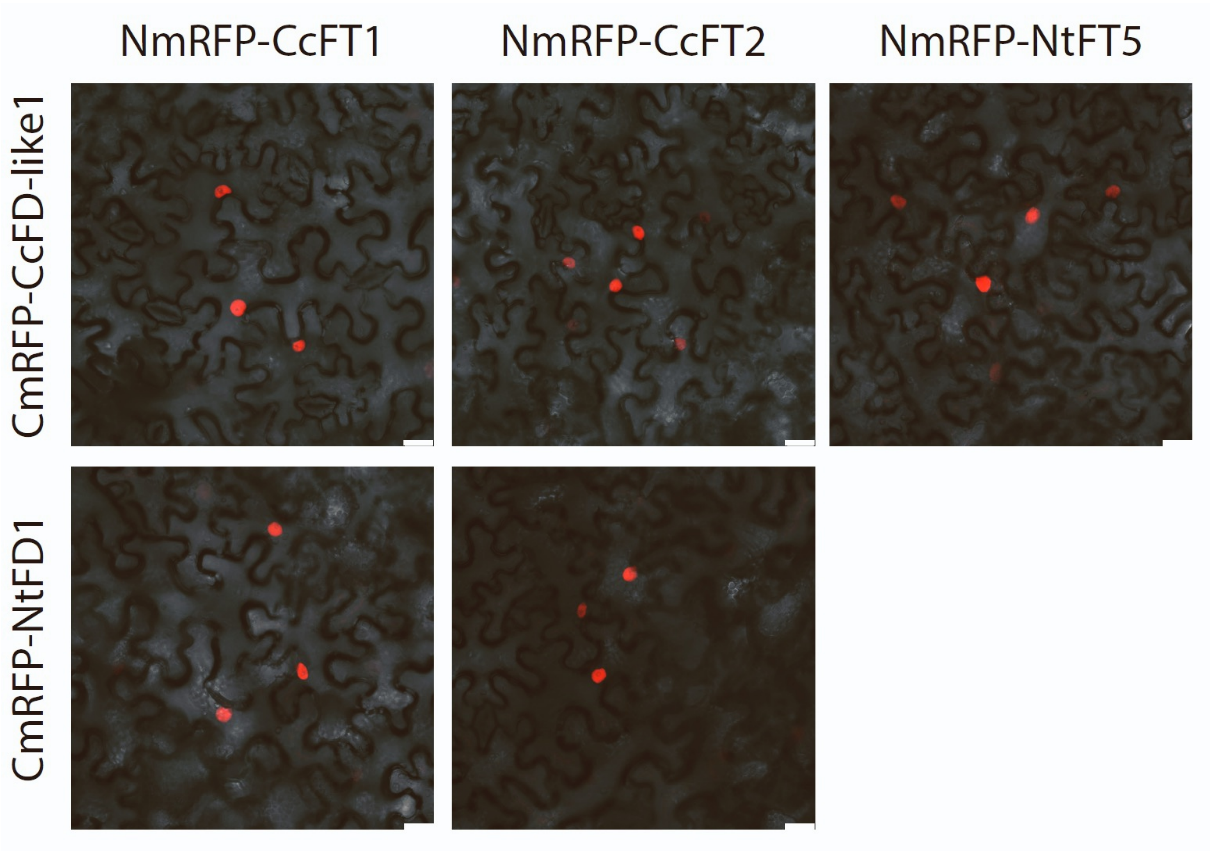

Supplemental Figure 5. Bimolecular fluorescence complementation assay in *Nicotiana benthamiana* epidermal cells with *Cuscuta* and tobacco FD/FT homologs. The FD homologs were C-terminally fused to the C-terminal mRFP1 fragment, and the FT homologs were C-terminally fused to the N-terminal mRFP1 fragment. Interaction was confirmed by reconstituted mRFP fluorescence visible in the nuclei. N-terminal or C-terminal mRFP1 fragments served as negative controls and were co-expressed with the corresponding FD or FT fusion proteins. We did not observe any nonspecific interactions in any combination. Scale bar = 20 µm.

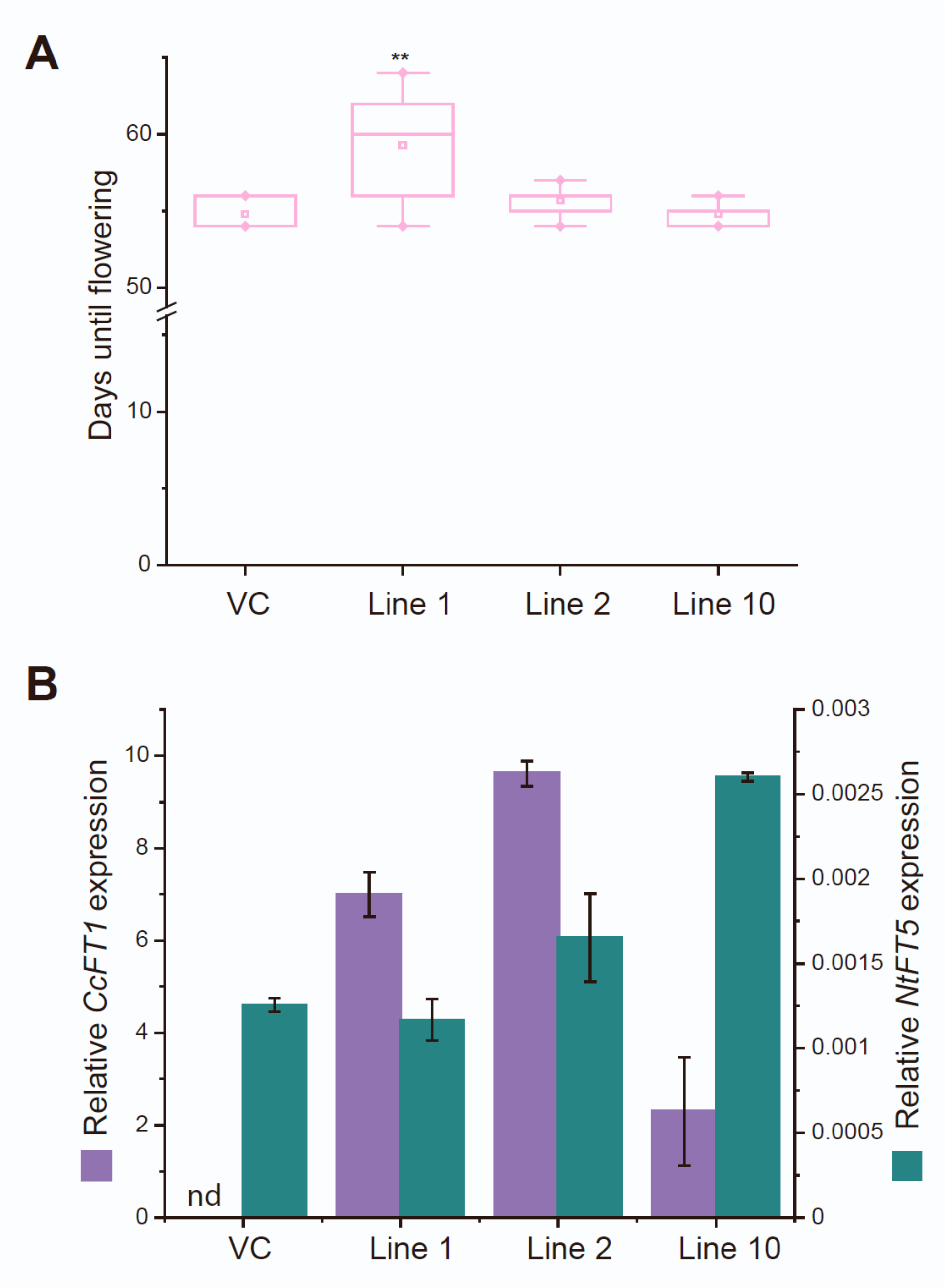

Supplemental Figure 6. Flowering time analysis of tobacco plants overexpressing *CcFT1*. A) Flowering phenotype of tobacco lines 1, 2, and 10 overexpressing *CcFT1* compared to vector controls (VC). Days until flowering were determined when the first bud had fully opened. **Statistically significant difference as determined by ANOVA and Tukey's *post hoc* test, p <0.01; *n* = 10 (lines 1, 2 and 10), *n* = 5 (VC). In the boxplots, center line = median, square = mean, box limits = upper and lower quartiles, whiskers = 1.5× interquartile range, and diamonds = outliers. **B)** Overexpression levels of *CcFT1* and endogenous expression levels of *NtFT5* in transgenic plants were determined when plants entered the reproductive growth phase. No *CcFT1* expression was detected in vector controls (nd). Data are means ± SEM, *n* = 3 biological replicates.

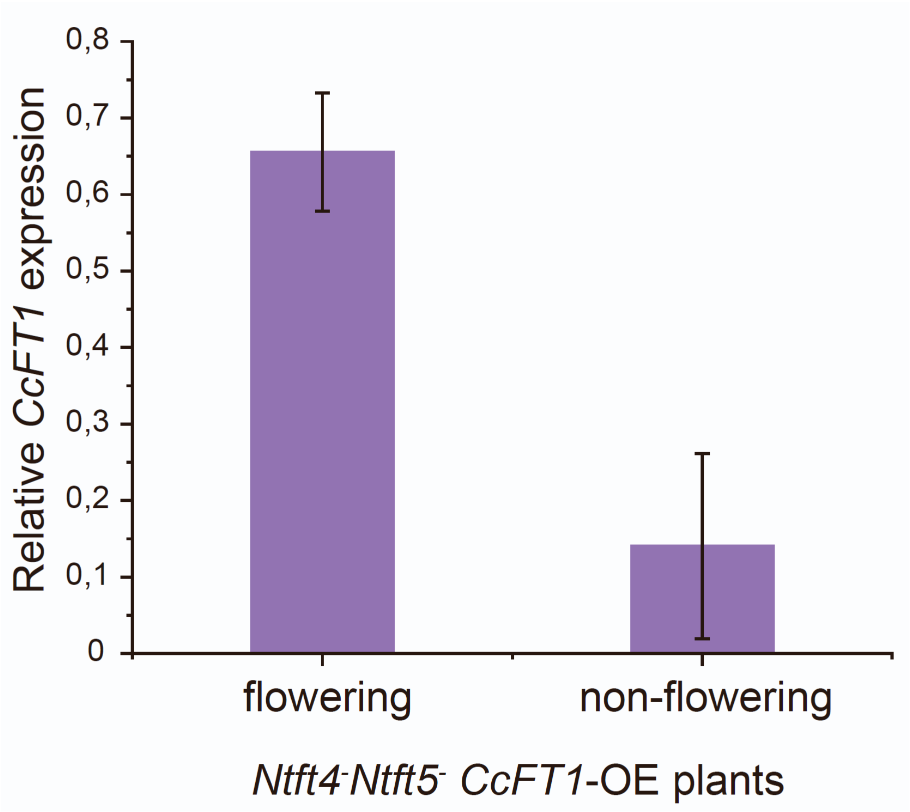

Supplemental Figure 7. Relative expression levels of *CcFT1* in flowering versus non-flowering *Ntft4*$^{-}$*Ntft5*$^{-}$ plants overexpressing *CcFT1*. Data are means ± SEM. *n* = 46 (flowering), *n* = 2 (non-flowering).

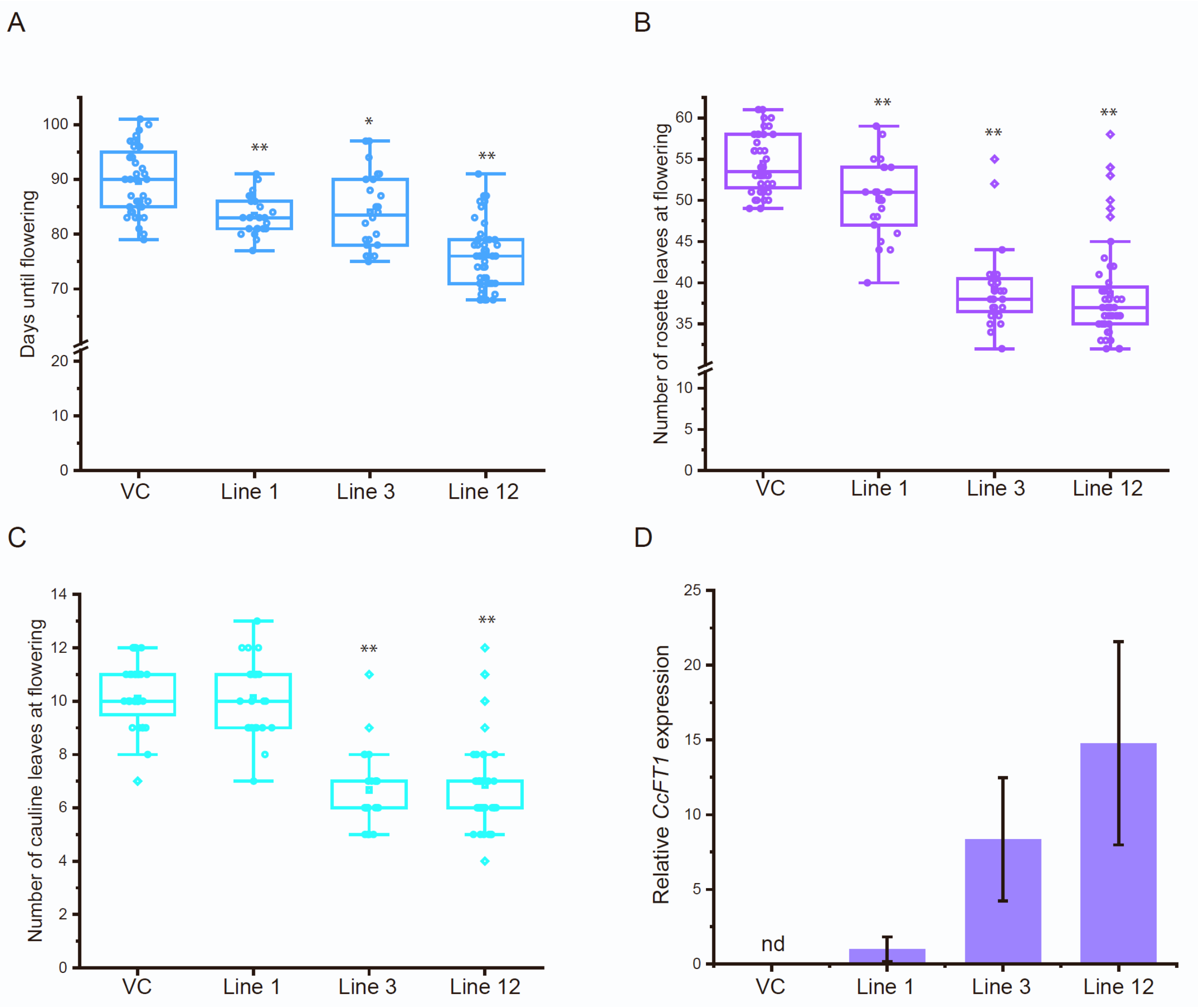

Supplemental Figure 8 Phenotypic analysis of *CcFT1* overexpressing *A. thaliana* Col-0 plants grown under SD conditions. A) Flowering phenotype of overexpression lines 1, 3, and 12 compared to vector controls (VC). Days until flowering were determined when the first bud had fully opened. **B)** Numbers of rosette leaves and **C)** cauline leaves were counted when the first bud had fully opened. *n* = 23 (line 1), *n* = 24 (line 3) and *n* = 48 (line 12), *n* = 40 (VC). In the boxplots, center line = median, square = mean, box limits = upper and lower quartiles, whiskers = 1.5× interquartile range, and diamonds = outliers. **D)** Overexpression levels of *CcFT1* in transgenic plants and vector controls were determined in seedlings. No *CcFT1* expression was detected in vector controls (nd). Data are means ± SEM, n ≥ 8 biological replicates. Statistically significant differences compared to vector controls was determined by Welch´s ANOVA and Games-Howell *post hoc* test for (A) and ANOVA and Tuckey´s *post hoc* test for (B-D), with *p = <0.05, **p <0.01.

Supplementary Tables

Supplemental Table 1. RNA Seq transcript levels.

		Cuscuta campestris total reads mapped: 1 990 329 102 (after trim)		*Cuscuta australis* total reads mapped: 908 853 342 (after trim)	
	**Gene length**	**Coverage**	**RPKM**	**Coverage**	**RPKM**
FT1	534 bp	4.56	57.01	12.98	633.80
FT2[1]	531 bp	4.68	64.81	--	--
FD-like	918 bp	1 025.49	11 596.03	398.02	16 046.96
SOC1	714 bp	43 406.26	498 893.10	6 731.04	297 048.13
TFL1	594 bp	1 724.57	22 286.12	5 020.35	226 723.48
LFY	1263 bp	236.22	2 592.57	313.55	13 852.78
CO-like	1047 bp	3 986.97	50 309.32	2 676.82	123 982.11
GI-like	3453 bp	8 752.11	97 400.03	1 981.41	79 456.96

Supplemental Table 2. RNA-Seq data for *Cuscuta campestris* and *C. australis*.

Organism	Tissue	SRA accession	Illumina read length, mode	Bases (Gbp)
C. australis	Stem	SRR19610423	151 bp, PE	6,445
C. australis	Stem	SRR19611357	151 bp, PE	6,505
C. australis	Stem	SRR19611520	151 bp, PE	6,386
C. australis	Stem	SRR19611521	151 bp, PE	5,863
C. australis	Stem	SRR19612874	151 bp, PE	5,699
C. australis	Stem	SRR19612875	151 bp, PE	6,616
C. australis	Stem	SRR19612878	151 bp, PE	6,220
C. australis	Stem	SRR19627926	151 bp, PE	6,231
C. australis	Stem	SRR19627939	151 bp, PE	6,261
C. australis	Stem	SRR20661662	151 bp, PE	6,186
C. australis	Stem	SRR20662442	151 bp, PE	6,547
C. australis	Stem	SRR20662443	151 bp, PE	5,744
C. australis	Germinating seedling	SRR6664647	151 bp, PE	6,087
C. australis	Haustoria on tomato	SRR6664648	151 bp, PE	7,236
C. australis	Bud	SRR6664649	151 bp, PE	8,374
C. australis	Ovary	SRR6664650	151 bp, PE	8,936
C. australis	Stem tip on tomato	SRR6664651	151 bp, PE	7,697
C. australis	Seeds	SRR6664652	151 bp, PE	7,329
C. australis	Pre-haustoria on tomato	SRR6664653	151 bp, PE	8,503
C. australis	Stems on tomato	SRR6664654	151 bp, PE	7,452
				**136,328**

C. campestris	Not specified*	ERR1916345	125 bp, PE	5,062
C. campestris	Not specified*	ERR1916346	125 bp, PE	1,832
C. campestris	Not specified*	ERR1916347	125 bp, PE	8,524
C. campestris	Not specified*	ERR1916348	125 bp, PE	20,608
C. campestris	Not specified*	ERR1916349	125 bp, PE	17,925
C. campestris	Not specified*	ERR1916350	125 bp, PE	7,865
C. campestris	Not specified*	ERR1916351	125 bp, PE	8,375
C. campestris	Not specified*	ERR1916352	125 bp, PE	8,777
C. campestris	Not specified*	ERR1916353	125 bp, PE	5,125
C. campestris	Not specified*	ERR1916354	125 bp, PE	2,518
C. campestris	Not specified*	ERR1916355	125 bp, PE	14,034
C. campestris	Not specified*	ERR1916356	125 bp, PE	6,807
C. campestris	Not specified*	ERR1916357	125 bp, PE	0.541
C. campestris	Not specified*	ERR1916358	125 bp, PE	24,504
C. campestris	Not specified*	ERR1916359	125 bp, PE	6,649
C. campestris	Not specified*	ERR1916360	125 bp, PE	1,202
C. campestris	Not specified*	ERR1916361	125 bp, PE	4,443
C. campestris	Not specified*	ERR1916362	125 bp, PE	13,772
C. campestris	Not specified*	ERR1916363	125 bp, PE	5,153
C. campestris	Not specified*	ERR1916364	125 bp, PE	3,823
C. campestris	Penetrating haustorium	SRR12763776	101 bp, PE	3,121
C. campestris	Attaching haustorium	SRR12763777	101 bp, PE	3,094
C. campestris	Attaching haustorium	SRR12763778	101 bp, PE	3,175
C. campestris	Attaching haustorium	SRR12763779	101 bp, PE	3,168
C. campestris	Swelling haustorium	SRR12763780	101 bp, PE	3,181
C. campestris	Swelling haustorium	SRR12763781	101 bp, PE	3,194
C. campestris	Swelling haustorium	SRR12763782	101 bp, PE	3,187
C. campestris	Vegetative stem	SRR12763783	101 bp, PE	3,176
C. campestris	Penetrating haustorium	SRR12763787	101 bp, PE	3,130
C. campestris	Penetrating haustorium	SRR12763788	101 bp, PE	3,204
C. campestris	Vegetative stem	SRR12763789	101 bp, PE	3,119
C. campestris	Vegetative stem	SRR12763790	101 bp, PE	3,095
C. campestris	Haustorial region	SRR13299802	51 bp, SE	0.387
C. campestris	Haustorial region	SRR13299801	51 bp, SE	0.396
C. campestris	Haustorial region	SRR13299800	51 bp, SE	0.618

C. campestris	Haustorial region	SRR13299799	51 bp, SE	0.714
C. campestris	Haustorial region	SRR13299798	51 bp, SE	0.172
C. campestris	Haustorial region	SRR13299797	51 bp, SE	0.301
C. campestris	Haustorial region	SRR13299796	51 bp, SE	0.212
C. campestris	Haustorial region	SRR13299795	51 bp, SE	0.308
C. campestris	Haustorial region	SRR13299794	51 bp, SE	0.225
C. campestris	Haustorial region	SRR13299793	51 bp, SE	0.283
C. campestris	Haustorial region	SRR14853881	151 bp, PE	8,619
C. campestris	Haustorial region	SRR14853883	151 bp, PE	8,954
C. campestris	Haustorial region	SRR14853885	151 bp, PE	11,372
C. campestris	Haustorial region	SRR14853887	151 bp, PE	9,394
				**247,357**

* Vogel et al. (2018) used “non-infectious feeding stems, detached starving stems and different infection stages”, p.8; PE – paired-end mode; SE – single-end mode.

Supplemental Table 3. Taxon list and accession numbers for FD-like proteins used in gene family analysis.

Species	Gene denomination (if any)	Locus tag, accession number
Arabidopsis thaliana	ABI5	AT2G36270
Arabidopsis thaliana	AREB3	AT3G56850
Arabidopsis thaliana	FD	AT4G35900
Arabidopsis thaliana	FDP (BZIP27)	AT2G17770
Brachypodium distachyon	BdFD1	Bradi3g60870
Brachypodium distachyon	BdFD2	Bradi4g36587
Brachypodium distachyon	BdFD3	Bradi1g29920
Cuscuta australis	BZIP-family gene	g21250.t1
Cuscuta australis	BZIP-family gene	g56588.t1-CDS
Cuscuta australis	BZIP-family gene	g2115.t1
Cuscuta australis	BZIP-family gene	g29401.t1
Cuscuta australis	*CaFD	*C046N0061E0.1
Cuscuta australis	CaFD-like	C001N0026E0.1;
Cuscuta australis	CaFD-like	C025N0100E0.1
Cuscuta australis	CaFD-like	C033N0055E0.1
Cuscuta australis	CaFD-like	C056N0011E0.1
Cuscuta australis	CaFD-like	C036N0249E0.1
Cuscuta campestris	CcFD-like	Ccam0.32, scaffold247, Cc032062
Cuscuta campestris	CcFD-like	Ccam0.32, scaffold2, Cc010070
Cuscuta campestris	CcFD-like	Ccam0.32, scaffold30, Cc002951
Cuscuta campestris	FD-like	Ccam0.32, scaffold218, Cc030291
Cuscuta campestris	CcFD1	Ccam0.32, scaffold223, Cc030608
Cuscuta campestris	CcFD2	Ccam0.32, scaffold48, OOIL02004257.1
Fragaria vesca	FvFD1	mrna14556.1-v1.0-hybrid
Fragaria vesca	FvFD2	mrna08556.1-v1.0-hybrid
Glycine max	GmFD1	Glyma04g02420
Glycine max	GmFD2	Glyma06g02470
Hordeum vulgare	HvFD1	BAJ91167
Hordeum vulgare	HvFD2	BAK04622
Hordeum vulgare	HvFD3	AK249012
Ipomoea nil	INIL11	g05356.t1
Ipomoea triloba	ItFD	XP_31117467.1
Malus domestica	MdFD1	MDP0000169473
Malus domestica	MdFD2	MDP0000636541
Musa acuminata	MaFD1	GSMUA_Achr1T02630
Musa acuminata	MaFD2	GSMUA_Achr5T11470_001
Musa acuminata	MaFD3	GSMUA_chr9G24090_001
Oryza sativa	n.a.	Os01g0813100
Oryza sativa	OsFD1	Os09g0540800
Oryza sativa	OsFD2	Os06g0720900
Oryza sativa	OsFD3	Os02g0833600

Oryza sativa	OsFD4	Os08g0549600
Oryza sativa	OsFD5	Os06g0724000
Oryza sativa	OsFD6	Os06g0719500
Oryza sativa	TRAB1	Os08g0472000
Phoenix dactylifera	PdFD1	PDK30s1175071g003
Piecea glauca	PgABI5-1	BT102312.1
Piecea glauca	PgABI5-2	BT110053.1
Pinus taeda	PtABI5	AEK86263.1
Popolus trichocarpa	PtFD1	POPTR_0005s11140.1
Popolus trichocarpa	PtFD2	POPTR_0005s26480
Setaria italica	SiFD1	Si031077m
Setaria italica	SiFD2	Si007412m
Setaria italica	SiFD3	Si023448m
Setaria italica	SiFD4	Si014546m
Solanum lycopersicum	SlFD2	Solyc02g061990.2.1
Solanum lycopersicum	SPGB	Solyc02g083520
Solanum tuberosum	StFD1	Sotub02g026810
Solanum tuberosum	StFD2	Sotub02g009830
Sorghum bicolor	SbFD1	Sb02g031340
Sorghum bicolor	SbFD2	Sb10g030400
Sorghum bicolor	SbFD3	Sb04g038600
Triticum aestivum	TaFD1	CK206464
Triticum aestivum	TaFDL13	ABZ91908
Triticum aestivum	TaFDL15	ABZ91909
Triticum aestivum	TaFDL2	ABZ91911
Triticum aestivum	TaFDL3	ABZ91912
Vitis vinifera	VvFD1	GSVIVT01009970001
Vitis vinifera	VvFD2	GSVIVT01006332001
Zea mays	DLF1	GRMZM2G067921
Zea mays	ZmFD2	GRMZM2G402862
Zea mays	ZmFD3	GRMZM2G073892

*CaFD sequence identified by Shen et al. (2020).

Supplemental Table 4. Taxon list and accession numbers of PEBP proteins for phylogenetic inference.

Species	Gene denomination (if any)	Locus tag, accession number
Aegilops tauschii	FT	ABI34864.1
Antirrhinum majus	CENTRORADIALIS-like	CAC21563.1
Antirrhinum majus	CENTRORADIALIS-like	CAC21564.1
Aquilegia formosa	TERMINAL FLOWER 0	ABB90591.1
Arabidopsis lyrata subsp. *lyrata*	CENTRORADIALIS-like	XP_2876387.1
Arabidopsis lyrata subsp. *lyrata*	TERMINAL FLOWER 0	AAM27957.1
Arabidopsis lyrata subsp. *lyrata*	TERMINAL FLOWER 1	XP_2873117.1
Arabidopsis lyrata subsp. *lyrata*	BROTHER of FT and TFL 1	XP_2864769.1
Arabidopsis thaliana	PEBP family protein	NP_176726.1
Arabidopsis thaliana	PEBP family protein	NP_193770.1
Arabidopsis thaliana	CENTRORADIALIS-like	NP_180324.1
Arabidopsis thaliana	PEBP family protein	NP_196004.1
Arabidopsis thaliana	PEBP family protein	NP_201010.1
Arabidopsis thaliana	PEBP family protein	NP_173250.1
Beta vulgaris	flowering locus T-like protein FT2	ADM92610.1
Beta vulgaris	flowering locus T-like protein FT1	ADM92608.1
Beta vulgaris	centroradialis-like protein CEN1	ADM92611.1
Brassica napus	BNTFL1-1	BAA33415.1
Brassica napus	BNTFL1-3	BAA33417.1
Brassica napus	BNTFL1-2	BAA33416.1
Brassica rapa	BRTFL1-1	BAA33418.1
Brassica rapa	BRTFL1-2	BAA33419.1
Capsicum annuum	self-pruning-like protein	AAZ66798.1
Capsicum frutescens	fasciculate	ACL27223.1
Carica papaya	flowering locus T	ACX85427.1
Chaenomeles sinensis	TFL1-like protein	BAD10965.1
Chaenomeles sinensis	TFL1-like protein	BAD10971.1
Chrysanthemum lavandulifolium	flowering locus T-like protein	ACY82397.2
Chrysanthemum x *morifolium*	flowering locus T-like protein	ACX48949.1
Chrysanthemum x *morifolium*	flowering locus T-Like protein	BAJ14266.2
Citrus sinensis	terminal flower	AAR04683.1
Citrus sinensis	terminal flower	AAR04684.1
Citrus trifoliata	CTRSFT1-like protein	ABY91244.1
Citrus trifoliata	CTRSTFL-like protein	ABY91242.1
Citrus trifoliata	CTRSTFL-like protein	ABY91243.1
Citrus unshiu	CiFT	BAA77836.1
Citrus unshiu	flowering locus T	BAF96644.1
Citrus unshiu	flowering locus T	BAF96645.1
Citrus unshiu	MOTHER of FT, TFL1-like protein	BAF93494.1
Crocus sativus	terminal flower 1-like protein	ACX53295.1
Cucumis sativus	FT-like protein	BAH28253.1
Cucumis sativus	TFL1-like protein	BAH28255.1
Cucurbita maxima	flowering locus T-like 2	ABI94606.1
Cucurbita maxima	flowering locus T-like 1	ABI94605.1
Cucurbita moschata	FTL2	ABR20499.1
Cucurbita moschata	FTL1	ABR20498.1
Cuscuta australis	FT, partial	Data Dryad
Cuscuta australis	C020N0378E0.1	CaFT
Cuscuta campestris	CcFT1	Data Dryad
Cuscuta campestris	CcFT2	Data Dryad

Cuscuta campestris	TL1-like partial	Data Dryad
Cydonia oblonga	TFL1-like protein	BAD10964.1
Cydonia oblonga	TFL1-like protein	BAD10970.1
Erythrante guttata	FT, partial	Data Dryad
Ficus carica	flowering locus T-like protein	BAI60052.1
Glycine max	Dt1	ADF30893.1
Glycine max	brother of FT and TFL1 protein	ABS57463.1
Glycine max	CETS1	ABF65987.1
Glycine max	mother of FT-like protein	ACA24491.1
Glycine max	unknown	ACU14433.1
Gossypium arboreum	terminal flower 1a	ABW24961.1
Gossypium arboreum	terminal flower 1b	ABW24962.1
Gossypium arboreum	terminal flower 1b	ABW24969.1
Gossypium hirsutum	terminal flower 1a	ABW24963.1
Gossypium hirsutum	terminal flower 1a	ABW24970.1
Gossypium hirsutum	terminal flower 1a	ABW24967.1
Gossypium hirsutum	terminal flower 1b	ABY62770.1
Gossypium hirsutum	terminal flower 1b	ABW24964.1
Gossypium raimondii	terminal flower 1a	ABW24965.1
Gossypium raimondii	terminal flower 1b	ABW24966.1
Helianthus annuus	flowering locus T4	ADF32945.1
Helianthus annuus	flowering locus T2	ADF32947.1
Helianthus annuus	terminal flower 1	ADO61015.1
Hordeum vulgare	FT	ABI55201.1
Hordeum vulgare	FT	ABI55202.1
Hordeum vulgare	FT	ABJ97441.1
Hordeum vulgare subsp. *vulgare*	vrn-H3 late flowering allele	ABK91684.1
Hordeum vulgare subsp. *vulgare*	FT-like protein	ABB99414.1
Hordeum vulgare subsp. *vulgare*	terminal flower 1-like protein	ABF85670.1
Hordeum vulgare subsp. *vulgare*	homologous protein to TFL1	BAH24197.1
Impatiens balsamina	TERMINAL FLOWER 1 protein	CAI61980.1
Impatiens balsamina	TERMINAL FLOWER 1 protein	CAI61982.1
Ipomoea nil	FT-like protein	ABW73563.1
Ipomoea nil	FT-like protein	ABW73562.1
Ipomoea nil	CENTRORADIALIS homolog	BAE44112.1
Ipomoea triloba	FT, partial	Data Dryad
Lactuca sativa	FT, partial	Data Dryad
Leavenworthia crassa	terminal flower 1-like protein	ADC32542.1
Lolium perenne	FT3	ABC33722.1
Lolium perenne	terminal flower 1-like protein	AAG31808.1
Lotus japonicus	CEN/TFL1-like protein	AAQ93599.1
Malus domestica	flowering locus T	ACL98164.1
Malus domestica	flowering locus T like protein	BAD08340.1
Malus domestica	CENTRORADIALIS like protein	BAG31957.1
Malus domestica	CENTRORADIALIS like protein	BAG31958.1
Malus domestica	TFL1-like protein	BAD06418.1
Malus domestica	TFL1-like protein	BAD10961.1
Malus domestica	TFL1-like protein	BAD10967.1
Malus domestica	TFL1 like protein	BAG31959.1
Medicago truncatula	PEBP	ABE80135.1
Misopates orontium	CEN/TFL1-like protein	CAJ44126.1
Nicotiana tabacum	NtFT5	Data Dryad
Nicotiana tabacum	NtFT4	Data Dryad

Nicotiana tabacum	CEN-like protein 2	AAD43529.1
Nicotiana tabacum	CEN-like protein 4	AAD43530.1
Nicotiana tabacum	CEN-like protein 1	AAD43528.1
Olea europaea	FT, partial	Data Dryad
Oncidium hybrid cultivar	flowering locus T	ACC59806.1
Oryza glumipatula	FT-like protein	BAH56285.1
Oryza longistaminata	FT-like protein	BAH30248.1
Oryza rufipogon	Hd3a	BAG72302.1
Oryza rufipogon	FT-like protein	BAH30250.1
Oxybasis rubra	Flowering locus T-like 1 protein	ABV56568.1
Oxybasis rubra	flowering locus T-like 2	ABP02016.1
Phyllostachys meyeri	FT-like protein	BAI49899.1
Phyllostachys meyeri	FT-like protein	BAI49900.1
Phyllostachys meyeri	FT-like protein	BAI49901.1
Picea abies	FT-like/TFL1-like protein	ABQ85553.1
Picea sitchensis	unknown	ABK25607.1
Picea sitchensis	unknown	ACN40175.1
Pisum sativum	TFL1a	AAR03725.1
Populus deltoides	flowering locus T-like protein FT1	AAS00056.1
Populus nigra	flowering locus T	BAD01561.1
Populus nigra	flowering locus T	BAD01612.1
Populus nigra	flowering locus T	BAD02371.1
Populus nigra	FLOWERING LOCUS T	BAG12904.1
Populus nigra	terminal flower 1	BAD22599.1
Populus nigra	flowering locus T like protein	BAD22601.1
Populus nigra	flowering locus T like protein	BAD27481.1
Populus nigra	flowering locus T like protein	BAD22677.1
Populus tremula	flowering locus T-like protein FT1	ABD52003.1
Populus trichocarpa	protein HEADING DATE 3A	XP_2316173.1
Populus trichocarpa	protein HEADING DATE 3A	XP_2311264.1
Populus trichocarpa	CEN-like protein 2	XP_2312811.1
Populus trichocarpa	CEN-like protein 1	XP_2321903.2
Populus trichocarpa	MFT-like protein	ABC26020.1
Prunus mume	flowering locus T like protein	CAQ16124.1
Prunus mume	TFL1-like protein	BAJ14521.1
Prunus persica	flower locus T	ACH73165.1
Pyrus communis	TFL1-like protein	BAD10963.1
Pyrus communis	TFL1-like protein	BAD10969.1
Pyrus pyrifolia	TFL1-like protein	BAD10962.1
Pyrus pyrifolia	TFL1-like protein	BAD10968.1
Raphanus sativus	terminal flower1-like protein	BAH37010.1
Rhaphiolepis bibas	TFL1-like protein	BAD10966.1
Rhaphiolepis bibas	TFL1-like protein	BAD10972.1
Sinapis alba	flowering locus T	ACM69283.1
Solanum lycopersicum	self-pruning protein	AAC26161.1
Solanum tuberosum	terminal flower 1 protein	ABC24691.1
Sorghum bicolor	protein HEADING DATE 3A	XP_2436509.1
Sorghum bicolor	protein TWIN SISTER of FT	XP_2443085.1
Sorghum bicolor	protein FLOWERING LOCUS T	XP_2454134.1
Sorghum bicolor	CEN-like protein 2	XP_2442808.1
Sorghum bicolor	CEN-like protein 2	XP_2450283.1
Sorghum bicolor	SELF-PRUNING	XP_2453931.1
Sorghum bicolor	FLOWERING LOCUS T	XP_2438551.1

Sorghum bicolor	FLOWERING LOCUS T	XP_2446272.2
Sorghum bicolor	FLOWERING LOCUS T	XP_2451827.2
Sorghum bicolor	HEADING DATE 3A	XP_2456354.1
Sorghum bicolor	MOTHER of FT/TFL1 homolog 2	XP_2457494.1
Spinacia oleraceae	FT, partial	Data Dryad
Triticum aestivum	putative kinase inhibitor	BAG12867.1
Triticum aestivum	flowering locus T	AAW23034.1
Triticum aestivum	flowering locus T	ACA25439.1
Triticum aestivum	flowering locus T, partial	ACA25438.1
Triticum monococcum	VRN3	ABK32206.1
Triticum turgidum	VRN3	ABK32207.1
Vaccinum darrowii	FT, partial	Data Dryad
Vicia faba	TFL1, partial	ABP73380.1
Vigna unguiculata	terminal flower 1a	BAJ22384.1
Vigna unguiculata	terminal flower 1b	BAJ22383.1
Vitis mustangensis	terminal flower 1	ADF80900.1
Vitis vinifera	flowering locus T-like protein	ABF56526.1
Vitis vinifera	FT-like protein	ABI99465.1
Vitis vinifera	flowering locus T	ABL98120.1
Vitis vinifera	TFL1A protein	ABI99466.1
Vitis vinifera	TFL1B protein	ABI99467.1
Vitis vinifera	TFL1C protein	ABI99468.1
Vitis vinifera	MFT-like protein	ABI99469.1

Supplemental Table 5. List of primers used in this study. Restriction sites in 5′-overhangs are underlined. Annealing temperatures (T) are given only for PCR primers.

Primer name	Sequence (5´to 3´)	T [°C]	purpose
Q35S-P SmaI fw	AGACCCGGGTCCAATCCCACCAAAAC	58	Generation of pLab12.10Q35S-P
Q35S-P SOE rev	GCTCTTATACTTGAGCGTGTCCT		
TMV Ω TL SOE fw	AGGACACGCTCAAGTATAAGAGC	58	
TMV Ω TL XhoI rev	AGACTCGAGATGGATAATTGTAAATGTAATTGTAATG		
CcFT1-like NcoI fw	AGACCATGGCGGCGAGGCGGCAGA	68	Cloning of pLab12.10Q35S-P::*CcFT1*
CcFT1-like XbaI rev	AGATCTAGACTATTGACGGCGCCTGCCGC		
CcFT2 PspXI fw	AGACTCGAGCA ACAATGGCGGCGAGGCGGCAGA	68	Cloning of pLab12.1Q35S-P::*CcFT2*
CcFT2-like XbaI rev	AGATCTAGACTATTAACGGCGCCTGCCTCCG		
CAS9 fw	ACGTGACCGAGGGAATGAGG	62	Analysis of transgene integration into the genome of *Ntft5*$^{-}$ plants
CAS9 rev	TTGCAGGAGATCCAGCGAGG		
NtFT4 ExI CRISPR T3 f	ATTGAAGGTCTGTTGATCTTAGAG	-	*NtFT4* protospacer sequence
NtFT4 ExI CRISPR T3 r	AAACCTCTAAGATCAACAGACCTT	-	
NtFT4 5'UTRf	GAAAAGTAAAGTATTTAGTGTATAG	48	Amplification of *NtFT4* (exon I) for sequencing (Schmidt et al., 2020)
NtFT4 Intr1 rev	ATGCGAATCAATTATAAATGG		
NtFT5 5'UTR for2	CCCTTAGACTTGTAAAAACATGC	53	Amplification of *NtFT5* (exon I) for sequencing (Schmidt et al., 2020)
NtFT5 Intr2 rev3	ATTGTAGATAGAGTTCCTAGCG		
CcFT-like NcoI fw	AGACCATGGCGGCGAGGCGGCAGA	68	Cloning of BIFC constructs
CcFT1-like XbaI rev	AGATCTAGACTATTGACGGCGCCTGCCGC		
CcFT-like NcoI fw	AGACCATGGCGGCGAGGCGGCAGA	68	
CcFT2-like XbaI rev	AGATCTAGACTATTAACGGCGCCTGCCTCCG		
CcFD-like NcoI fw	AGACCATGGGATCTCAGGGCGGTGGT	61	
CcFD-like NheI rev	AGAGCTAGCTCAAAAAGGATTCGAGCTTGTTC		
CcFT ATG fw	ATGGCGGCGAGGCGGCAGA	68	Identification of *CcFT1* genomic sequence
CcFT ExonII rev	CACTTGGACTTGGAGCATCAGGATC		
CcFT ExonII fw	GTGATGGTGGATCCTGATGCTC	63	
CcFT1 gDNA1 II rev	TTTAAGTGGGCATTGATTGTTGGTGTCA		
CcFT1 gDNA2 fw	TGACACCAACAATCAATGCC	61	
CcFT1 gDNA2 rev	TCGTCTGGAAGTCCATACCCAG		
CcFT1 gDNA3 fw	TCCAGACGAACAATCGTGTTA	59	
CcFT1 gDNA3 rev	AAATTAGGAGTGACGTATTCTTAT		
CcFT1 gDNA4 fw	TGGATAATCGATCTTTTAGAGTCA	59	
CcFT1 gDNA4 rev	TCTGTGATACTGTGATGCA		
CcFT1 gDNA5 fw	AAAAGTTAGGAGAACACCAAAT	60	
CcFT1 gDNA5 rev	TATACCTTTCCACTTGATGACA		
CcFT1 gDNA6 fw	AGTAGCACTTTTAGTTGAGTCA	59	
CcFT1 gDNA6 rev	AAGTCAAACTGTGACTATCTT		
CcFT1 g_gap fw	GTGTCCAAGTCATTCTCGGATC	62	
CcFT1 g_gap rev	CACCTTTGCCGCAAGTAGTTG		
CcFT1 gDNA8 fw	GAAGTTCTCTGAACTCACACAAG	59	
CcFT1 gDNA8 rev	GATAGAAATTAGTTGATAACATTAGGT		
CcFT1 gDNA9 fw	ACCTAATGTTATCAACTAATTTCTATC	59	
CcFT1 gDNA9 rev	TTTAGTTTTGAACTCTTAAACCTTAAA		
CcFT1 gDNA10 fw	TTTAAGGTTTAAGAGTTCAAAACTAAA	61	
CcFT1 gDNA10 rev	AGACCCGGAGCAAATAGAT		

CcFT1 gDNA11 fw	GGTCTGGACCTATGGTCTG	61	
CcFT1 gDNA11 rev	AGTTCACATTGACTCTATGAGTT		
CcFT1 gDNA12 fw	AACTCATAGAGTCAATGTGAACT	59	
CcFT1 gDNA12 rev	CCTCACACTAAGACATTATTAAG		
CcFT ATG fw	ATGGCGGCGAGGCGGCAGA	68	Identification of *CcFT2* genomic sequence
CcFT ExonII rev	CACTTGGACTTGGAGCATCAGGATC		
CcFT ExonII fw	GTGATGGTGGATCCTGATGCTC	64	
CcFT2-IntII_rev	GGAGAGGGATTTAAGGAGAGTGTA		
CcFT2 gDNA1 fw	TACCCATCACATCAAAGGAC	59	
CcFT2 gDNA1 rev	CATAACTCATTTCACACTTGTC		
CcFT2 gDNA2 fw	GGCATAATGAACGAATTCGAC	59	
CcFT2 gDNA2 rev	GTCTTAGTGGAAGGAATAATGC		
CcFT2 gDNA3 fw	CCCGTTCCGAATTAATCCTA	58	
CcFT2 gDNA3 rev	ACCTTTGCCGCAAGTAGTTC		
CcFT2 gDNA4 fw	AGTCAAGCGTGCTTGACTTGAAG	64	
CcFT2 gDNA4 rev	TGCTATGAGAATCCTAGAACTAGCCA		
CcFT2 gDNA5 fw	CACGTGAGAAGGGACATG	59	
CcFT2 gDNA5 rev	TCTGCTCGTGTACGATTACAG		
CcFT2 gDNA6 fw	CATACAGAGTATATCTCAATCCTAGA	59	
CcFT2 gDNA6 rev	CCAAAATTAACTCCGGTGCTCG		
CcFT2 g_gap1 fw	CTTTCTGACCAAGTACGCCAC	62	
CcFT2 g_gap1 rev	CGTTCAGCCACAGACTACCTACAG		
CcFT2 g_gap2 fw	CACCCAGGAAGACACAGTAACC	62	
CcFT2 g_gap2 rev	GTCAGTTGGGAAAGCATGTGC		
CcFT2 g_gap3 fw	GGATTGCCATAAGTCTGTATGC	62	
CcFT2 g_gap3 rev	GCTGGAACAGTACAAATACGAACC		
CcFT2 gap2 seq fw	CTGCATCAGAATAAGCCTGTAG	-	
CcFT2 gap2 seq rev	GTAGTTTTCGACGAGCACAC	-	
M13 fw	GTAAAACGACGGCCAG	-	Sequencing of subcloned PCR products
M13 bw	CAGGAAACAGCTATGAC	-	
3´Oligo_d(T)	GCTGTCAACGATACGCTACGTAACGGCATGACAGTGT TTTTTTTTTTTTTTTTTTTTTTTT	-	cDNA synthesis for 3´-RACE
GSP1 fw	TGCTATGAAAGCCCGCAACC	64	3′-RACE of *CcFT1* and *CaFT* (1. PCR)
3´Adap	GCTGTCAACGATACGCTACGTAACG		
GSP2 fw	TCGTACTGTTCCAGCAGATG	60	3′-RACE of *CcFT1* and *CaFT* (2. PCR)
3´Adap_ nested	CGCTACGTAACGGCATGACAGTG		
CcFT ATG fw	ATGGCGGCGAGGCGGCAGA	64	Amplification of *CcFT1*/*CcFT2*/*CaFT* cDNA
3′-UTR CcFT rev	GCGCACGGTGGATTATTGTTCT		
qPCR CcActin fw	ATGGAAGCTGCTGGAATCCAC	62	Expression analysis of *Cuscuta* spp. *Actin* by qRT-PCR (Rezaei et al., 2017)
qPCR CcActin rev	TTGCTCATACGGTCAGCGATG		
qPCR CaEF1α fw	TCAGACTGTTGCTGTGGGTG	62	Expression analysis of *Cuscuta* spp. *EF1a* by qRT-PCR (Zhang et al., 2014)
qPCR CaEF1α rev	CCCTTGTGCGGTTCACTTCT		
qPCR CcFT1 disc fw	GGAGAGGTGCTTGAGCCT	62	Expression analysis of *CcFT1* and *CaFT* by qRT-PCR
qPCR CcFT1 disc rev	CGGGCTTTCATAGCATACC		
qPCR CcFT2 disc fw	GTGATCGGAGAGGTGCTG	62	Expression analysis of *CcFT2* by qRT-PCR
qPCR CcFT2 disc rev	CTGTGGGCTTTCATAACATACA		
qPCR CcFD fw	ACCGATGACCATGTCTCCAT	62	

qPCR CcFD rev	CTTGCTCTCAAGCTCCTGTG		Expression analysis of *CcFD*-like and *CaFD* by qRT-PCR
qRTNt/Ntom EF-1α for	AAGCTGACTGTGCTGTCCTGA	67	Expression analysis of *NtEF1α* by qRT-PCR (Beinecke et al., 2018)
qRTNt/Ntom EF-1α rev	GGTGGTAGCATCCATCTTGTTG		
qRTNtFT5/NtomFTy for discr	TCCCAAGTTATTAACCAGCCC	67	Expression analysis of *NtFT5* by qRT-PCR (Beinecke et al., 2018)
qRTNtFT5/NtomFTy rev discr	CATAGCACACAATTTCCTGGC		
qPCR GmF-Box fw	AGATAGGGAAATGGTGCAGGT	62	Expression analysis of *GmF-Box* by qRT-PCR (Libault et al., 2008)
qPCR GmF-Box rev	CTAATGGCAATTGCAGCTCTC		
qRT Ubi_HKG1 fw	CCAAGCCGAAGAAGATCAAG	62	Expression analysis of *AtUbi* by qRT-PCR
qRT Ubi_HKG1 rev	ATGACTCGCCATGAAAGTCC		
qRT PP2_HKG2 fw	ACAGCTACGGTCTGGTGTTTTC	62	Expression analysis of *AtPP2* by qRT-PCR
qRT PP2_HKG2 rev	TCGTGTCGAATTTTCCCGTGATG		

Supplemental Table 6. Data for CcFT1 and/or CcFt2 expression, time until flowering (d) and number of leaves at the time of flowering on tobacco and Arabidopsis.

Plant ID	Relative Expression	Days until flowering	No. of leaves at onset of flowering
**CcFT1 - overexpression on tobacco**			
1#1	0.2067730661	90	26
1#2	0.1728595650	91	27
1#4	0.1802287676	89	25
1#5	0.3753132132	76	21
1#6	0.9017726117	74	22
1#7	0.5033205683	90	26
1#8	1.3516376759	68	17
1#10	0.3751998785	69	25
1#12	0.5654169017	67	18
1#13	0.2841872852	74	22
1#14	0.2954170555	74	24
1#15	1.3288333524	74	22
1#16	0.5889524353	83	23
1#17	0.5001842534	92	23
1#18	0.8493423675	71	20
1#19	0.4829208173	77	23
1#20	3.3859477121	82	17
6#1	0.6343422469	129	72
6#2	0.4548091970	160	81
6#5	0.4061261982	75	24
6#6	1.1224620483	81	26
6#7	0.3789291416	95	35
6#8	0.4590320100	207	38
6#10	0.5396141183	74	21
6#11	0.2952481654	109	34
6#12	0.4741230156	183	33
6#13	1.1566881839	74	20
6#14	0.9308797161	205	42
6#15	0.3314048604	131	75
6#16	0.2206757491	102	38
6#17	0.8467453124	94	40
6#18	0.6141515747	107	53
9#2	0.6282041082	74	22
9#3	0.4554078601	70	21
9#5	0.3913214324	74	21
9#6	1.4419544036	71	21
9#7	0.5131743556	71	20
9#8	0.5243743157	70	21
9#10	0.6221000786	88	24
9#11	0.6302383947	73	22
9#12	0.3106841572	75	21
9#13	1.0686859862	90	21
9#14	0.4932683898	88	22

9#15	0.8379214912	90	23
9#17	0.8774354893	70	22
9#18	0.1339198935	74	22
9#20*	0.0192464231	>183	>70
6#4*	0.2612192882	>183	>70
***CcFT2* - overexpression in tobacco**			
2#1	2.037591234	102	26
2#2	2.823738176	83	28
2#3	2.095012615	90	30
2#5	3.019717501	74	30
2#7	1.247004635	76	26
2#8	1.642040414	77	26
2#9	1.656559291	83	28
2#10	4.011194804	85	33
2#11	4.641326827	73	24
2#12	4.037542538	76	25
2#16	2.185010505	91	31
2#18	1.872439854	77	28
4#1	14.6249495	74	20
4#2	7.428662026	73	21
4#3	13.40485268	73	19
4#4	4.186376451	74	23
4#5	5.424698488	74	25
4#6	7.827817545	75	22
4#7	2.105576421	81	28
4#8	9.131467961	74	21
4#9	4.802371353	74	25
4#11	5.869765608	74	23
4#13	2.509631926	77	26
4#14	5.264760997	74	23
4#15	2.794895058	83	26
4#16	7.353500122	76	23
4#17	8.351269009	73	23
4#18	3.785101609	76	21
4#20	4.805185577	76	27
8#1	2.133443179	89	30
8#3	3.030158757	71	23
8#4	1.581568999	76	27
8#5	2.345474659	74	22
8#6	1.807947341	74	23
8#7	5.182002715	70	22
8#8	4.280542984	70	22
8#9	3.213035066	73	25
8#10	2.238721346	81	26
8#11	0.795006423	130	40
8#12	2.103198431	81	22
8#13	4.780948872	71	22

8#14	7.036158485	70	21	
8#16	1.813303493	112	32	
8#17	2.950274677	73	23	
8#18	2.579505831	74	21	
8#19	6.165470159	70	21	
8#20	6.546006148	70	22	

CcFt overexpression on *Arabidopsis*

Sample	Relative CcFT1 expression	Days until flowering	Rosette leaves	Cauline leaves
L01 #01	0.14396	88	51	10
L01 #08	0.32303	80	44	7
L01 #09	0.00019	90	58	13
L01 #10	7.62724	86	51	11
L01 #11	0.31998	84	46	9
L01 #13	0.26894	79	45	9
L01 #17	0.27342	77	48	9
L01 #21	0.00063	85	54	11
L01 #22	0.00054	91	55	11
L03 #01	0.00121	85	38	7
L03 #11	0.25602	76	34	6
L03 #12	33.98078	84	37	6
L03 #15	6.20036	83	41	7
L03 #17	6.7494	76	37	5
L03 #18	16.01514	97	41	7
L03 #20	0.11643	75	37	6
L03 #22	3.53241	94	38	5
L12 #08	51.22309	77	37	6
L12 #17	18.70006	77	38	6
L12 #18	0.68332	77	39	7
L12 #26	0.00111	91	48	6
L12 #31	0.05698	68	33	7
L12 #34	34.42638	68	38	6
L12 #38	0.00025	87	58	10
L12 #42	13.10201	68	34	6

* non-flowering individuals

Supplemental Datasets

Dataset S1 (*separate file*). Results of exonerate-based genic conservation analysis of 295 flowering time regulating genes in *Cuscuta* spp. and 13 related eudicots. Gene and reading frame conservation is provided as length identity score from exonerate-based *protein2genome* alignments, using validated reference proteins of *Arabidopsis thaliana* as queries. In addition to genomic data searches of *Cuscuta* spp., we also examined transcriptome assemblies, the results of which given in separate columns. Finally, we computed the divergence of *Cuscuta* flowering gene conservation relative to that of the mean length identity score of the 13 nonparasitic eudicots, whereby values >1 indicate stronger conservation and/or longer reading frames in *Cuscuta* spp. than the average, and values <1 suggest less conservation and/or shorter genes in the parasites. Datasets with recovered genic fragments are published as reusable fasta files in the Dryad Data Repository, https://datadryad.org/stash/share/DK8Olh2VqFwbGNL0GtkGt24dD0GhWhJn82oLBC1XK70.

Dataset S2 (*separate file*). Domain-based annotation of transposable elements (DANTE) and inferred fragments. Filtered and unfiltered database hits of DANTE searches within the extended *CaFT*, *CcFT1* and *CcFT2* gene regions are summarized by length, identity, similarity, and number of interruptions. The retrieved and reference transposable element domains are provided for every hit, indicating interruptions such as gaps and stop codons by backslashes and asterisks, respectively. Datasets with recovered genic fragments are published as reusable fasta files in the Dryad Data Repository, https://datadryad.org/stash/share/DK8Olh2VqFwbGNL0GtkGt24dD0GhWhJn82oLBC1XK70.
